# Impact of Ceftiofur Administration in Steers on the Prevalence and Antimicrobial Resistance of *Campylobacter* spp.

**DOI:** 10.3390/microorganisms9020318

**Published:** 2021-02-04

**Authors:** Sicun Fan, Derek Foster, William G. Miller, Jason Osborne, Sophia Kathariou

**Affiliations:** 1 Department of Food, Bioprocessing and Nutrition Sciences, North Carolina State University, Raleigh, NC 27695, USA; sfan3@ncsu.edu; 2 Department of Population Health and Pathobiology, College of Veterinary Medicine, North Carolina State University, Raleigh, NC 27695, USA; dmfoster@ncsu.edu; 3 Produce Safety and Microbiology Research Unit, Agricultural Research Service, U.S. Department of Agriculture, Albany, CA 94710, USA; william.miller@usda.gov; 4 Department of Statistics, College of Sciences, North Carolina State University, Raleigh, NC 27695, USA; jaosborn@ncsu.edu

**Keywords:** ceftiofur, CCFA, CHCL, cattle, *Campylobacter*, antibiotic resistance

## Abstract

Bacterial resistance to ceftiofur raises health concerns due to ceftiofur’s extensive veterinary usage and structural similarity with the human antibiotic ceftriaxone. Ceftiofur crystalline-free acid (CCFA) and ceftiofur hydrochloride (CHCL) are ceftiofur types used therapeutically in cattle, but their potential impacts on *Campylobacter* prevalence and antimicrobial resistance remain unclear. In this study two groups of steers were each treated with CCFA or CHCL. In vivo active drug concentrations were measured and fecal samples were analyzed for *Campylobacter* for up to 42 days post-treatment. Following administration, the colonic concentration of ceftiofur initially increased then dropped to pre-treatment levels by day 8. The estimated prevalence of *Campylobacter* spp. was significantly (*p* = 0.0009) higher during the first week after CCFA treatment than after CHCL treatment (81.3% vs. 45.2%). *Campylobacter jejuni* predominated overall, with other *Campylobacter* spp. mainly identified in the first week after CCFA treatment. No treatment impacts were noted on ceftiofur minimum inhibitory concentration (MIC) for *C. jejuni* (10–20 μg/mL). More *C. jejuni* genotypes were detected in CCFA-treated than CHCL-treated steers. These findings suggest that ceftiofur did not significantly impact *Campylobacter* prevalence or ceftiofur MIC. However, CHCL may be preferable due to the lower likelihood of temporary increases in *Campylobacter* prevalence.

## 1. Introduction

The use of antimicrobials in livestock can promote animal health and productivity [1], but also increases concerns related to emergence and persistence of antimicrobial resistance in zoonotic bacterial pathogens [2,3]. Among livestock, the highest domestic use of medically-important antimicrobials is in cattle (42%) [4]. These antimicrobials include the 3rd-generation cephalosporins (3GCs) [5], which are also identified as the most critically-important antimicrobials for human medicine [6].

Ceftiofur is a 3GC that is exclusively used in veterinary medicine. In the United States, ceftiofur is one of the leading antimicrobials for feedlot cattle and lactating dairy cows, and is frequently employed therapeutically to treat respiratory disease in feedlot cattle and postpartum metritis in cows [7,8,9]. In addition, ceftiofur has been employed extra-label for the treatment of enteric disease in cattle [10], even though most extra-label administrations of cephalosporins were banned by the United States Food and Drug Administration in 2012 [11].

Major reasons for the common use of ceftiofur in beef and dairy cattle include its high effectiveness [12,13], zero withdrawal time for milk [14,15,16], and short withdrawal periods for meat [15,17,18]. However, ceftiofur is similar in structure to the antibiotic ceftriaxone which is used extensively in human medicine, and the two drugs have the same mode of action [19,20,21]. Concerns arise, therefore, that the use of ceftiofur for cattle conditions such as respiratory disease, metritis, and enteric disease can lead to ceftriaxone resistance in bacterial foodborne pathogens such as *Salmonella enterica*, *Escherichia coli*, and *Campylobacter* spp. These foodborne bacterial pathogens can inhabit the bovine gastrointestinal tract, typically without symptoms, and become transmitted to humans via the food chain [21,22].

Injectable ceftiofur formulations in the market currently include ceftiofur crystalline-free acid (CCFA; Excede^®^), ceftiofur hydrochloride (CHCL; Excenel^®^), and ceftiofur sodium sterile powder (CSSP; Naxcel^®^) [23]. These formulations have varied indications, approved dosages, routes of administration, and meat withdrawal periods [24]. In addition, they vary in pharmacokinetic parameters, including peak concentrations and half-life [25]. There is no clear differentiation among these formulations regarding treatment efficacy, and the choice is mainly based on convenience [17]. The potential impacts of different formulations of ceftiofur on zoonotic foodborne pathogens and their antimicrobial resistance remain poorly understood and in need of further investigation. Currently, only a few reports are available on the impact of ceftiofur treatment of cattle on the gut microbiome and antimicrobial resistance genes, and on specific enteric bacteria such as *E. coli*, *Salmonella*, and *Enterococcus faecalis* in the feces of the animals [16,17,25,26].

*Campylobacter* spp. (family *Campylobacteraceae*) are Gram-negative, corkscrew-shaped bacteria that are highly motile and the only obligate microaerophiles among foodborne bacterial pathogens of humans [27]. The species most commonly implicated in human foodborne disease is *Campylobacter jejuni* followed by *C. coli*, and leading sources of transmission to humans include raw or undercooked poultry, raw milk, untreated water, and direct contact with animal feces [28]. *Campylobacter*, primarily *C. jejuni*, is a leading bacterial foodborne pathogen in the United States and other nations [29]. Common outcomes of campylobacteriosis include acute gastroenteritis characterized by diarrhea and severe abdominal pain, and campylobacteriosis is also the leading antecedent for the severe autoimmune disease Guillain-Barré syndrome [30]. Campylobacteriosis is typically a self-limited disease and treatment, when mandated, involves fluoroquinolones or macrolides, with the latter being increasingly preferred due to the high prevalence of fluoroquinolone resistance in *C. jejuni* and *C. coli* [31,32].

*Campylobacter* is routinely found in cattle and typically carried asymptomatically [27,33,34]. However, *Campylobacter* from bovine feces can contaminate milk, water or meat during processing [34]. Previous studies identified exposure to cattle and raw ground beef as risk factors for human campylobacteriosis [35,36]. In addition, numerous outbreaks of campylobacteriosis have been attributed to consumption of raw milk [37,38,39]. Furthermore, drug-resistant *Campylobacter* is listed among the “serious threats” in the most recent Antimicrobial Resistance Threats Report by the Centers for Disease Control and Prevention [32]. As discussed above, ceftiofur is frequently used therapeutically in dairy and beef cattle [7,8,9,10]. However, currently little is known regarding the potential impacts of ceftiofur treatment on the prevalence and antimicrobial resistance of *Campylobacter* in cattle.

In a previous study, we monitored the intestinal concentrations of CCFA and CHCL after treatment of steers with these drugs and investigated the impact of the treatment on microbiome composition as well as on the prevalence and minimum inhibitory concentration (MIC) of ceftiofur of *E. coli* in the feces of the animals [17]. We noted shifts in microbiome composition and decreased *E. coli* levels, especially in the CCFA-treated group, as well as temporary increases in the MIC of ceftiofur in both treatment groups with MIC values peaking at 96 and 48 h following the CCFA and CHCL treatment, respectively [17]. In the current study, our objective was to investigate the impact of CCFA (sustained but lower intestinal drug concentrations) and CHCL (shorter duration but higher intestinal drug concentrations) treatment of these steers on *Campylobacter* prevalence, tolerance to ceftiofur and other antimicrobials and multilocus sequence typing (MLST)-based genotypes. We hypothesized that the treatment impact may differ between cattle receiving CCFA and those receiving CHCL. Fecal samples were analyzed for *Campylobacter* before and after treatment in the two separate groups of steers that were administered CCFA and CHCL, respectively, and *Campylobacter* prevalence was assessed in the context of the drug concentration levels in the colon.

## 2. Materials and Methods

### 2.1. Steers and Treatment

The steers for this study and their treatment have been previously described [17]. They consisted of 12 healthy Holstein steers (six months old and weighing 186–288 kg) and were obtained from the Dairy Educational Unit at North Carolina State University (NCSU) in Raleigh, NC, USA. They were housed in pairs in stalls at the College of Veterinary Medicine (CVM), NCSU, from May 2016 to July 2016, as described in [17]. The steers for the CCFA treatment (*n* = 6) were here designated D1–D6 while those for CHCL (*n* = 6) were designated N1–N6. The CCFA treatment study took place from May 18 through 8 June 2016 for steers D1, D2, D3, and D4 and from 1 June through 13 July 2016 for steers D5 and D6. The CHCL treatment was from 24 June through 12 July 2016 for steers N1, N2, N3, and N4 and from June 29 through 12 July 2016 for steers N5 and N6. As previously described [17], the sample size was determined based on previous pharmacokinetic-pharmacodynamic studies in cattle [25,40,41]. All steers received lidocaine and flunixin meglumine for disbudding within the first month of life, while steer D6 received flunixin and CHCL for four days due to diarrhea and fever, several months prior to the CCFA treatment.

The ceftiofur treatments of the steers were previously described [17]. Briefly, ultrafiltration probes for drug level determination were placed in the ileum and spiral colon as described [17]. Within 24–48 h after placement of the probes, the steers were administered either CCFA (6.6 mg/kg, one dose; Zoetis, Parsippany, NJ, USA) or CHCL (2.2 mg/kg, three daily doses; Zoetis). Both drugs were injected subcutaneously, either at the base of the ear (CCFA) or in the neck (CHCL) [17]. This study was conducted under the approval of the NCSU Institutional Animal Care and Use Committee (protocol # 18-020A).

### 2.2. Gastrointestinal (GI) Fluid Collection, Active Drug Concentration Determination, and Pharmacokinetic Analysis

The drug concentration levels in the ileum and spiral colon were determined as described before, and have been previously reported [17]. Briefly, the active drug concentration in the intestinal fluid was measured using reverse-phase high pressure liquid chromatography with ultraviolet detection [17,25,42]. The pharmacokinetic analysis was performed using standard methods and the pharmacokinetic parameters were calculated by a pharmacokinetic computer program (Phoenix, V. 8.0; Pharsight Corporation, Certara, St. Louis, MO, USA) as described [17]. As previously indicated, for CCFA the fluid samples were collected immediately prior to treatment (time 0) and at 2, 4, 8, 12, 24, 32, 48, 72, 96, 120, 144, 168, and 192 h after treatment; for CHCL the collecting points were immediately prior to treatment (time 0) and at 2, 4, 6, 8, 12, 24, 26, 28, 30, 32, 36, 48, 50, 52, 54, 56, 60, 72, 74, 78, and 96 h after the first dose [17].

### 2.3. Sample Collection and Processing for Campylobacter

Fecal samples (hereafter frequently referred to as “samples”) were collected using sterile lubricant and a clean rectal sleeve as described [17,25,41]. The sampling points were largely similar for both treatment groups: 0 h (i.e., immediately before treatment), every 12 h until 72 h post-treatment, and daily thereafter until day 8, with samples also collected at less regular intervals thereafter up until day 35–42 for CCFA steers, and day 13–18 for the CHCL group (Table S1). The collected samples were packed individually, delivered to the laboratory overnight on ice and processed upon arrival.

To isolate *Campylobacter*, fecal samples were processed as described [43], unless stated otherwise. Specifically, 100 mg of each sample was added to 2 mL of Bolton broth (Oxoid Ltd., Basingstoke, Hampshire, UK), containing the corresponding supplement (SR183E, Oxoid) and laked horse blood (SR048C, Oxoid), in sterile 15-mL polypropylene Corning tubes (Fisher Scientific, Pittsburgh, PA, USA). The tubes were vortexed gently, covered loosely with caps, and incubated microaerobically at 37 °C overnight in anaerobic jars with GasPak EZ CampyPak container system sachets (Becton Dickinson, Sparks, MD, USA). Then, 100 µL of the overnight enrichment was serially diluted with sterile Mueller–Hinton broth (MHB; Difco, Becton Dickinson), plated (100 µL) onto modified charcoal-cefoperazone-deoxycholate agar (mCCDA; Oxoid) containing the corresponding supplement (SR0155E, Oxoid), and incubated microaerobically for 48 h at 37 °C. For a set of samples from the CCFA group (steers D1, D2, D3, and D4) the mCCDA plates were also incubated microaerobically at 42 °C for 48 h. Under the assumption that even one CFU in the original sample (100 mg) would yield a positive result after enrichment and plating, the limit of detection for *Campylobacter* was 10 CFU/g of feces.

Up to seven colonies per positive sample were chosen randomly from the mCCDA plates at 37 °C and purified under microaerobic conditions on Mueller–Hinton agar incubated at 37 °C for 48 h (MHA; Difco, Becton Dickinson; Table S1). Colonies from those mCCDA plates that had been incubated at 42 °C were purified on MHA following incubation at 42 °C for 48 h. A culture derived from a single colony is hereafter frequently referred to as a *Campylobacter* “isolate”. Pure cultures were preserved at −80 °C in brain heart infusion broth (BHI; Becton Dickinson) with 20% sterile glycerol. *Campylobacter* recovery and isolates from each animal and time point are listed in Table S1.

### 2.4. Campylobacter Species Determination, Multilocus Sequence Typing (MLST), and Minimum Spanning Tree (MST)

Genomic DNA was extracted from pure *Campylobacter* cultures using the DNeasy Blood and Tissue Kit (Qiagen, Valencia, CA, USA) following the manufacturer’s protocol. Multiplex PCR was performed using the *hipO* and *ceuE* primers to identify *C. jejuni* and *C. coli*, respectively, as described [44]. *Campylobacter* isolates that were negative for *C. jejuni* or *C. coli* were tested via multiplex PCR using *sapB2* and 23S rRNA primers specific for *C. fetus* and *Campylobacter* spp., respectively, as described [45], with the minor modification of extension at 72 °C for 1 min. *Campylobacter* isolates that were PCR-negative for *C. jejuni*, *C. coli* or *C. fetus* were analyzed by MLST as described below and found to be *C. hyointestinalis* based on the *C. fetus/C. hyointestinalis* MLST scheme [46].

MLST analysis was performed for 67 isolates as described [46,47]. For each steer, isolates were chosen to represent the first and last instance of encountering a specific species-antimicrobial resistance (AMR) profile combination, except for combinations which were encountered only once and were therefore represented by a single isolate (Table S2). The sequence type (ST) of *C. jejuni* D3-5d-42-A was determined in silico upon analysis of whole genome sequence data that were available for this strain through another study, via Sequence Query, PubMLST (https://pubmlst.org/bigsdb?db=pubmlst_campylobacter_seqdef) (Table S2). Novel STs were submitted to the *C. jejuni/C. coli* MLST database (http://pubmlst.org/campylobacter/) for ST assignment. The relationships among the STs are displayed by a minimum spanning tree (MST) constructed using BioNumerics (version 7.6.3) as described [47].

### 2.5. Campylobacter Antimicrobial Susceptibility and MIC Determinations

All isolates were routinely tested for resistance to a panel of antimicrobial agents (tetracycline, streptomycin, nalidixic acid, ciprofloxacin, erythromycin, kanamycin, and gentamicin) as described [48,49], using the pan-sensitive (PS) *C. jejuni* ATCC 33560 as a quality assurance control. To assess potential changes of the minimum inhibitory concentration (MIC) of ceftiofur over time, 25 *C. jejuni* isolates were selected from the CCFA (*n* = 19) and CHCL (*n* = 6) groups (Table S3) based on the following criteria: they originated from the same steer at the same incubation temperature, had the same species and AMR combination and the same or closely-related ST, and were isolated at different times. In addition to ceftiofur (1, 10, 20, and 30 μg/mL), this panel was tested for MICs of tetracycline (1 to 128 μg/mL), kanamycin (1 to 256 μg/mL), nalidixic acid (1 to 128 μg/mL), ciprofloxacin (1 to 32 μg/mL), ampicillin (1 to 32 μg/mL), and enrofloxacin (1 to 32 μg/mL).

### 2.6. Statistical Analysis

To estimate the effect of incubation temperature on the prevalence of *C. jejuni* from cattle in the CCFA treatment group, a generalized linear mixed model was fit using the GLIMMIX procedure of SAS version 9.4 (SAS Institute, Cary, NC, USA). The binary response variable was the presence (yes or no) of *C. jejuni* at each sampling time from 0 h (i.e., before treatment) to 8 days post-treatment from CCFA steers D1, D2, D3, and D4 with the explanatory variable being the incubation temperature (37 °C or 42 °C). Individual cattle and sampling time were considered as random effects to account for the interventions. The estimated prevalence of *C. jejuni* was derived from this model using SAS 9.4. The same statistical analysis was also performed for the non-*C. jejuni*, non-*C. coli* isolates.

To visualize the prevalence of *C. jejuni* per sampling point in the CCFA (steers D1, D2, D3, and D4) and CHCL treatment groups (steers N1, N2, N3, N4, N5, and N6) at 37 °C, the statements PROC MEANS and PROC GPLOT (SAS 9.4) were used. By visually checking the graphical distribution, the sampling time points were grouped into week 0 (immediately before treatment, 0 h), week 1 (12 h to day 7), and week 2 (day 8). To estimate the effect of the treatment type (CCFA or CHCL) on the prevalence of *C. jejuni*, i.e., the estimated prevalence at different weeks, a generalized linear mixed model with binomial response was fit by the GLIMMIX procedure in SAS 9.4. The response variable was the clustered prevalence of *C. jejuni* at each week from week 0 to week 2; the explanatory variables were the treatment type, week, and their interaction. Variability among individual cattle was modeled by including random effects for cattle. The estimated prevalence of *C. jejuni* and all detected *Campylobacter* spp., i.e., combining *C. jejuni*, *C. fetus*, and *C. hyointestinalis*, was derived from this model using SAS 9.4. Statistical significance was set at *p*-value 0.05.

## 3. Results

### 3.1. Impacts of CCFA and CHCL on Prevalence of Campylobacter spp.

**Figure 1:**
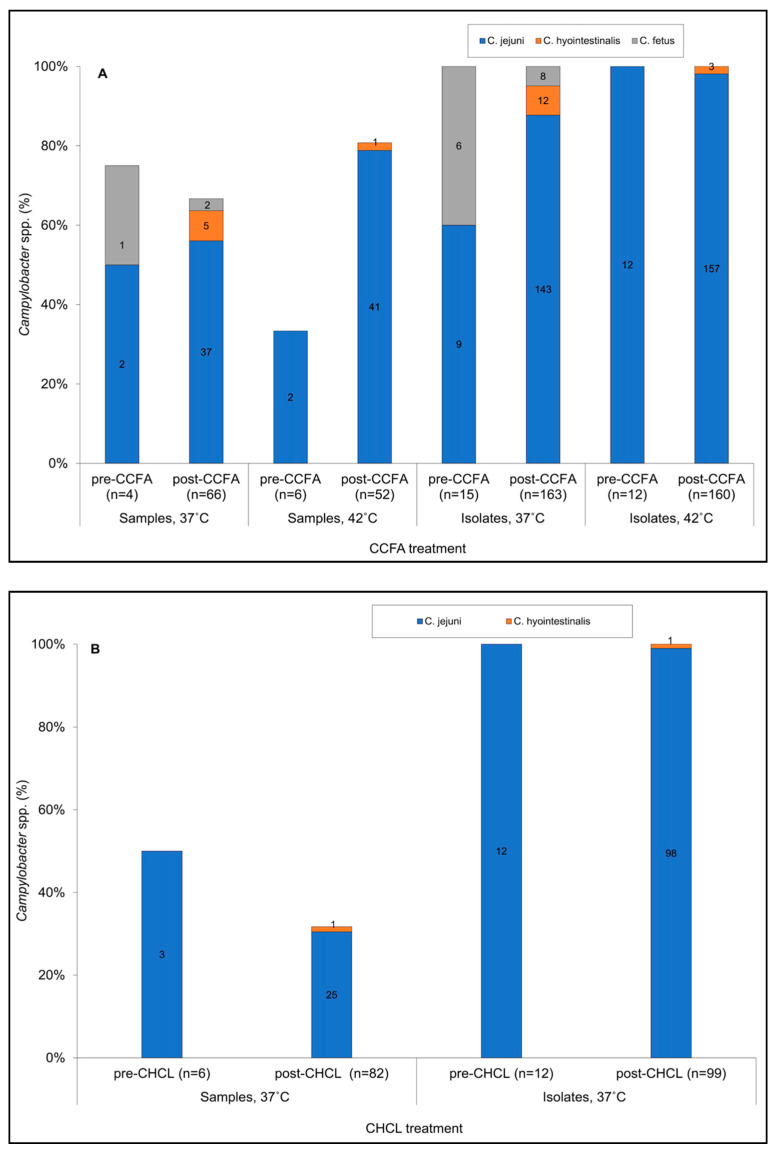
Prevalence of the different *Campylobacter* species in fecal samples from (**A**) ceftiofur crystalline-free acid (CCFA) and (**B**) ceftiofur hydrochloride (CHCL) groups. *Campylobacter* species designations are as in the inset, with blue for *C. jejuni*, orange for *C. hyointestinalis*, and gray for *C. fetus*. Pre-CCFA or pre-CHCL correspond to data pre-treatment (time 0), while post-CCFA and post-CHCL correspond to the compilation of all data post-treatment. “Samples” refers to fecal samples collected at different time points from the steers, while “Isolates” refers to *Campylobacter* cultures that were recovered from the fecal samples following enrichment and single colony purification. The four panels shown for the CCFA group in (**A**) correspond to, from left to right, (**1**), data from all fecal samples following incubation at 37 °C; (**2**), data from all fecal samples following incubation at 42 °C; (**3**), data from all *Campylobacter* isolates obtained following incubation at 37 °C; and (**4**), data from all *Campylobacter* isolates obtained following incubation at 42 °C. The two groups shown for the CHCL group in (**B**) correspond to data from all fecal samples (**left**) and data from all *Campylobacter* isolates (**right**). CHCL samples were only incubated at 37 °C. *Campylobacter* detection and species determinations were as described in Materials and Methods.

A total of 168 fecal samples, 80 and 88 from the CCFA and the CHCL group, respectively, were analyzed for *Campylobacter* spp. At pre-treatment (time 0), three of the six steers in each treatment group were found to be *Campylobacter*-positive. For the CCFA samples, two fecal samples (one/steer; steers D2 and D3) were *Campylobacter*-positive at both 37 °C and 42 °C and yielded *C. jejuni,* while the third (steer D4) was only positive at 37 °C and yielded *C. fetus* (Table S1). Similar findings were obtained for the samples from the CHCL group, which were processed only at 37 °C. Specifically, of the six steers in this group three (N4, N5, N6) were *Campylobacter*-positive and yielded *C. jejuni* (Table S1). However, all 12 steers were positive for *Campylobacter* post-treatment (Table S1). The prevalence of *Campylobacter*-positive samples post-treatment varied by temperature in the CCFA group, being higher at 42 °C (42/52, 80.8%) than at 37 °C (44/66, 66.7%) (Figure 1A). As indicated above, the CHCL samples were only examined at 37 °C, and 26 of the 82 post-treatment samples (31.7%) were *Campylobacter*-positive (Figure 1B). Due to the apparent impact of temperature on prevalence that was observed with the CCFA samples, the comparative assessment of the impact of CCFA vs. CHCL treatment on *Campylobacter* was assessed at the common temperature of 37 °C.

**Figure 2:**
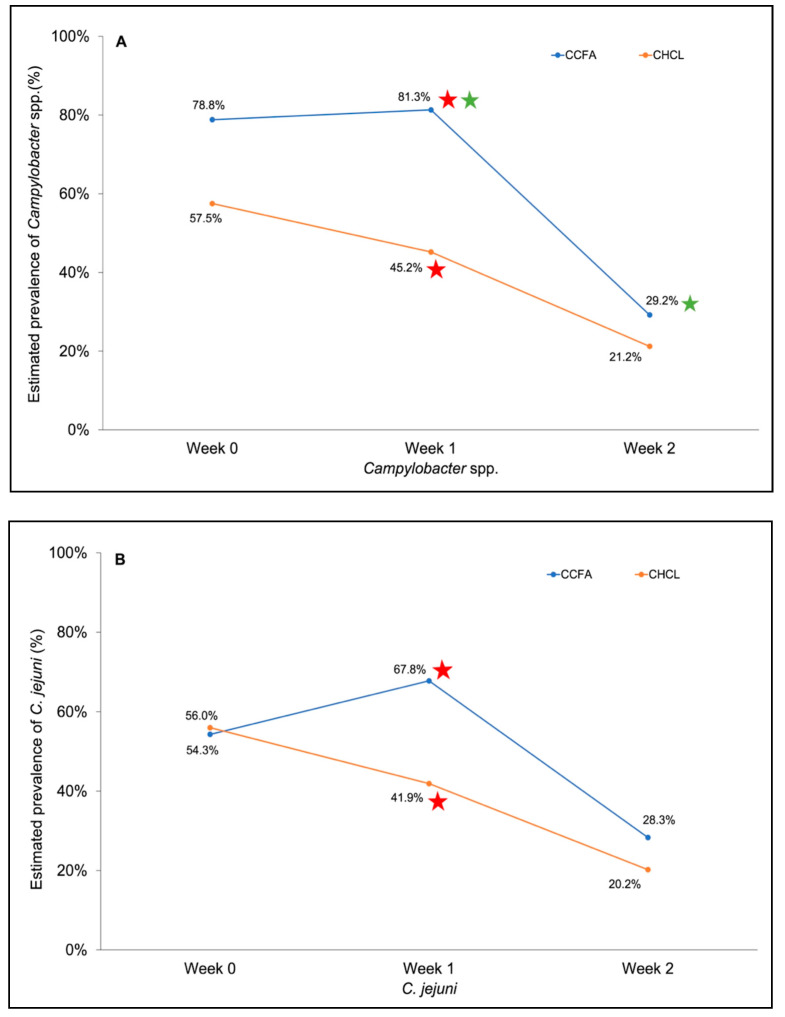
Estimated prevalence of (**A**) total *Campylobacter* spp. and (**B**) *C. jejuni* from the CCFA and CHCL groups. Red stars indicate a significant difference of estimated prevalence of *Campylobacter* spp. (*p* = 0.0009) or *C. jejuni* (*p* = 0.0184) between the CCFA and CHCL groups. Green stars indicate a significant difference (*p* = 0.0108) of the estimated prevalence of *Campylobacter* spp. between week 1 and 2 under CCFA treatment. Weeks 0, 1 and 2 correspond to pre-treatment (0 h), 12 h to day 7, and day 8, respectively. Data are based on CCFA and CHCL samples processed at 37 °C. Estimated prevalence determinations were performed as described in Materials and Methods.

In the CHCL group the estimated prevalence of *Campylobacter*-positive samples was higher pre-treatment than one week post-treatment (57.5% vs. 45.2%: Figure 2A), but significant immediate impacts of treatment were not noted in either group. On the other hand, cattle treated with CCFA had a significantly higher estimated prevalence of *Campylobacter* at week 1 vs. week 2 (81.3% vs. 29.2%; *p* = 0.0108), while the differences did not achieve similar significance for the CHCL group (*p* = 0.0689) (Figure 2A). Furthermore, even though the estimated prevalence of *Campylobacter* did not differ significantly between the two groups pre-treatment, at week 1 post-treatment it was significantly higher in the CCFA group (*p* = 0.0009) (Figure 2A).

Regardless of treatment group, the majority of the *Campylobacter*-positive samples yielded *C. jejuni* (Figure 1), and the estimated prevalence of *C. jejuni* largely reflected the trends described above for total *Campylobacter*-positive samples. It was similar between CCFA and CHCL samples prior to treatment (54.3% vs. 56.0%), but in week 1 after treatment it was significantly higher in CCFA than CHCL (67.8% vs. 41.9%; *p* = 0.0184; Figure 2B). CCFA samples also had a nearly-significant (*p* = 0.062) higher estimated prevalence of *C. jejuni* at week 1 vs. week 2 (67.8% vs. 28.3%) while for CHCL the estimated prevalence was more stable (Figure 2B).

### 3.2. Incubation Temperature Impacted the Estimated Prevalence of C. jejuni and Other Campylobacter spp.

As indicated above, the majority of the *Campylobacter*-positive samples from either CCFA or CHCL steers yielded *C. jejuni* (Figure 1). For those CCFA samples that were processed at both 37 °C and 42 °C, the estimated prevalence of *C. jejuni* was significantly higher at 42 °C (*p* = 0.0435; 79.7% vs. 59.5%). *C. coli* was not isolated from any of the samples, while other *Campylobacter* spp. were encountered only occasionally, and mostly in the CCFA samples at 37 °C (Figure 1). Interestingly, *C. hyointestinalis* was detected in the CCFA samples only post-treatment and was recovered from 7.6% and 1.9% of the post-treatment samples at 37 °C and 42 °C, respectively (Figure 1A), while in the CHCL group *C. hyointestinalis* was detected only once, again post-treatment (Figure 1B). *C. fetus* was identified only in the CCFA samples at 37 °C, with higher prevalence pre-treatment than post-treatment (25% vs. 3.0%; Figure 1A). Surprisingly, *C. fetus* was not detected in the CHCL group at any time, even though 37 °C was employed for *Campylobacter* isolation and, as mentioned above, *C. hyointestinalis* was only encountered once in this group. Even though the infrequent recovery of non-*C. jejuni Campylobacter* spp. prevented significance assessments, the collective data suggest that their recovery was favored at 37 °C post-treatment.

### 3.3. The Prevalence of Campylobacter-Positive Samples Reflected the Drug Concentration in the Colon, But C. jejuni Ceftiofur MIC Was Not Impacted by Either Treatment

**Figure 3:**
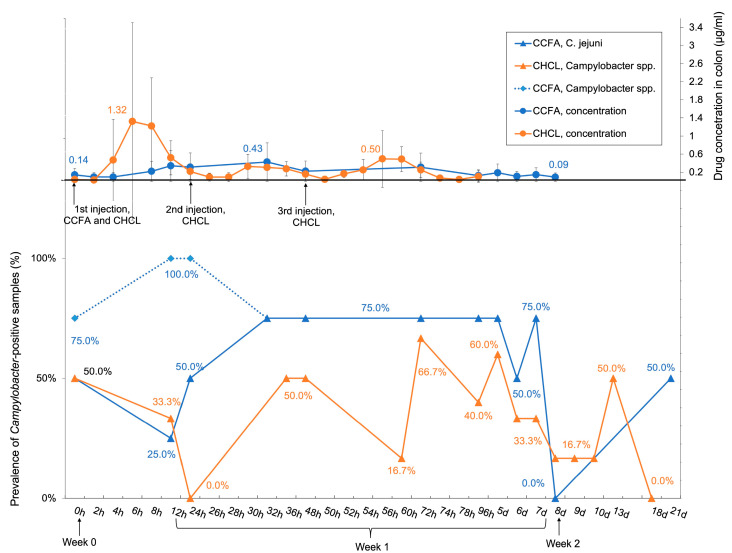
Drug concentrations in the colon and prevalence of *Campylobacter*-positive samples from the CCFA and CHCL groups. **Upper panel**, concentrations of CCFA (blue) and CHCL (orange). These drug concentration data were also presented earlier [17]. **Lower panel**, prevalence of *Campylobacter* spp. in CCFA (blue dashed lines) and CHCL (solid orange lines). Solid blue lines indicate prevalence of *C. jejuni* in the CCFA samples. *C. jejuni* prevalence in CHCL is not shown, as it was almost identical to total *Campylobacter* spp. Prevalence data are based on CCFA and CHCL samples processed at 37 °C. The concentrations of the drugs in the colon and prevalence of *Campylobacter* spp. and *C. jejuni* were determined as described in Materials and Methods.

Drug concentrations in the colon were monitored until day 8 post-treatment. The prevalence of *C. jejuni* during this time largely followed the trends in drug concentration. In the CCFA group, the drug concentration increased following administration and eventually dropped to pre-treatment levels by day 8, while in the CHCL group the levels reached three peaks at 6, 30, and 56 h, corresponding to the repeated administration of the drug, and then dropped to the pre-treatment levels by day 8 (Figure 3). The half-life of CCFA in the colon was approximately 2-fold longer (94.85 h) than CHCL (39.45 h) [17]. The prevalence of total *Campylobacter* spp. was similar to *C. jejuni* except at 12 and 24 h post-treatment in the CCFA group, when most samples positive for *C. fetus* and *C. hyointestinalis* were identified in the CCFA-treated animals (Figure 3).

A panel of *C. jejuni* representing different time points prior to and subsequent to CCFA and CHCL treatment were examined for their tolerance to ceftiofur. Isolates were chosen from those steers that had yielded *C. jejuni* prior to treatment, i.e., at time 0 (Table S1). No impact of treatment on ceftiofur MIC could be noted among any of the isolates (Tables S1 and S3). MIC varied somewhat by animal (10 µg/mL for isolates from D2 vs. 20 µg/mL for those from D3, N5, and N6), but the levels were the same regardless of time before and after treatment. In addition, for the tested isolates from the CCFA group, the MIC was the same for isolates from the same animal regardless of whether they were obtained at 37 °C or 42 °C (Tables S1 and S3). Similar to ceftiofur, examination of a panel of other antimicrobials (e.g., nalidixic acid, kanamycin, ciprofloxacin, and enrofloxacin) also failed to reveal consistent impacts of treatment on MICs (Table S3).

### 3.4. Isolates Resistant to Both Tetracycline and Kanamycin Predominated Pre- and Post-Treatment in BOTH Treatment Groups

**Figure 4:**
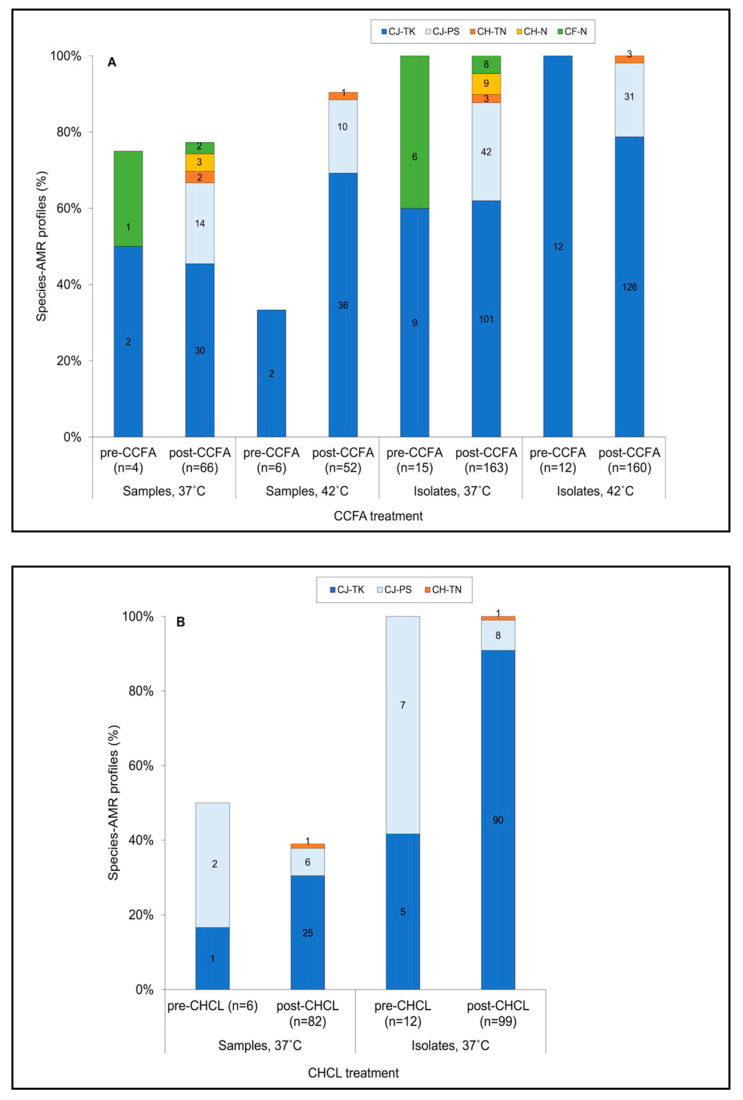
Prevalence of different species and antimicrobial resistance (AMR) profiles in *Campylobacter* samples and isolates from (**A**) CCFA and (**B**) CHCL groups. Species-AMR combinations are as in inset where CJ, CH, and CF indicate *C. jejuni*, *C. hyointestinalis*, and *C. fetus*, respectively. AMR abbreviations: TK, resistance only to tetracycline and kanamycin; PS, pan-susceptible; TN, resistance only to tetracycline and nalidixic acid; N, resistance only to nalidixic acid. Pre-CHCL or -CCFA referred to 0 h, while post-CHCL or -CCFA was a compilation of all sampling points after 0 h. A total of 12 samples from CCFA (seven at 37 °C and five at 42 °C, all post-treatment) and six from CHCL (post-treatment) yielded both *C. jejuni* TK and *C. jejuni* PS. “Samples” and “Isolates” are defined as described in the legend of Figure 1. *Campylobacter* detection from the fecal samples and determination of species and AMR profiles were as described in the Materials and Methods.

Testing the *C. jejuni* isolates with a panel of seven antimicrobials other than ceftiofur, specifically tetracycline, streptomycin, nalidixic acid, ciprofloxacin, erythromycin, kanamycin, and gentamicin, revealed just two AMR profiles: pan-susceptible (PS) and with resistance to only tetracycline and kanamycin (TK) (Table S1 and Figure 4). Ceftiofur MICs did not appear to vary between *C. jejuni* PS and *C. jejuni* TK isolates, with an MIC of 20 µg/mL being determined for both AMR profiles (Tables S1 and S3). *C. jejuni* TK isolates were encountered in each treatment group, both pre- and post-treatment. In the CCFA group, for which isolates were obtained at both 37 °C and 42 °C, the use of the higher temperature (42 °C) appeared to favor the detection of samples positive for *C. jejuni* TK (Figure 4A). Therefore, the potential impact of CCFA vs. CHCL treatment on recovery of *C. jejuni* TK was assessed at the common temperature of 37 °C. These comparisons suggested that the prevalence of *C. jejuni* TK-positive samples was similar before and after treatment in the CCFA group (Figure 4A), while it was higher post-treatment in the CHCL samples (Figure 4B). However, the pre- and post-treatment differences in the CHCL group were not statistically assessed due to a lack of power from limited samples and isolates pre-treatment.

It was noted that AMR profiles differed between *C. jejuni* and the other *Campylobacter* species. While *C. jejuni* isolates were either TK or PS, *C. hyointestinalis* were resistant either to tetracycline and nalidixic acid (TN) or only to nalidixic acid (N), and *C. fetus* were only resistant to nalidixic acid (N) (Figure 4). Samples positive for *C. hyointestinalis* TN were found only post-treatment, with similar prevalence (around 2.0%) in CCFA and CHCL samples, while *C. hyointestinalis* N was only detected in three post-treatment samples of the CCFA group (Figure 4).

### 3.5. Multilocus Sequence Typing (MLST) Analysis Suggests that CCFA Treatment May Favor the Diversity of C. jejuni

**Figure 5:**
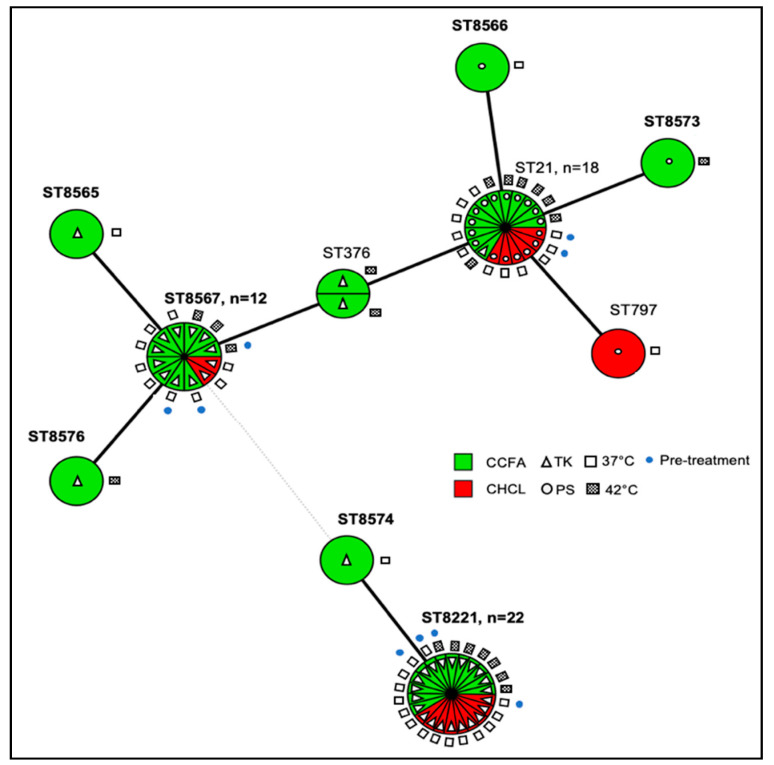
Minimum spanning tree (MST) demonstrating multilocus sequence typing (MLST)-based sequence types (STs) in *C. jejuni* from the CCFA and CHCL treatment groups. Each ST is represented by a circle, and partitions within each circle represent individual isolates with the respective ST. Circles without partitions (e.g., ST-797) indicate STs encountered only once. Green and red indicate the CCFA and CHCL group, respectively. AMR profile abbreviations: TK (rectangles), resistance only to tetracycline and kanamycin; PS (circles), pan-susceptible. Open and gray rectangles represent isolates obtained at 37 °C and 42 °C, respectively, and blue dots represent isolates recovered pre-treatment. Solid lines connect STs with a single-locus difference. The dashed line represents three or more allele differences, i.e., a different clonal complex (CC). STs above the dashed line belong to CC21, while the two STs below the dashed line belong to CC61. Novel STs are in bold. MLST analysis and MST construction were as described in Materials and Methods.

As indicated earlier, the sequence type (ST) of a panel of 68 isolates was determined with 50 from the CCFA group and 18 from the CHCL group. These isolates represented, for each treatment, the first as well as (when applicable) the last date of each species-AMR combination (Table S2). The panel included 60 *C. jejuni* with 10 MLST-based STs (Figure 5) as well as six *C. hyointestinalis* with two STs and two *C. fetus*, both of the same ST (Table S2). Of the 10 *C. jejuni* STs, 7 were novel (Table S2 and Figure 5).

Three STs, i.e., ST-21 (CC21) and the two novel STs 8567 (CC21) and 8221 (CC61), markedly predominated in both the CCFA and the CHCL isolates and were encountered both pre- and post-treatment in each group (Figure 5). In addition, they were detected in isolates obtained at both 37 °C and 42 °C (Figure 5). Overall, a greater number of STs were noted among the CCFA than the CHCL isolates (9 vs. 4, respectively). Of the six STs found only in the CCFA group, three, i.e., ST-376 and the novel STs 8573 and 8576, were only identified in isolates obtained at 42 °C (Figure 5). Nevertheless, even at 37 °C the CCFA group isolates (*n* = 21) exhibited three novel STs (STs 8565, 8566, and 8574) unique to this treatment group, all among the post-treatment isolates, while no novel STs unique to the CHCL group were identified among the 18 isolates from the CHCL treatment (Figure 5). The novel STs identified post-treatment in the CCFA group were closely related to other predominant STs recovered from the animals. Thus, STs 8573 and 8566 had single-allele differences from ST-21; ST-8574 also differed from ST-8221 by one allele; and STs 8565 and 8576 had one-allele differences from ST-8567 (Figure 5). Collectively, the findings suggest that CCFA treatment may be associated with higher genotypic diversity, but potential impacts of treatment on diversity cannot be statistically assessed due to a lack of power from the limited number of isolates.

Isolates with the same AMR profile tended to have the same ST, with the sole exception of one *C. jejuni* strain of ST-21 which was resistant to kanamycin and tetracycline, while all other ST-21 isolates were pan-susceptible (Figure 5). A similar correlation between STs and AMR profile was noted with *C. hyointestinalis*, for which all isolates of ST-6 were resistant to tetracycline and nalidixic acid, while those of ST-41 were resistant only to nalidixic acid (Table S2).

## 4. Discussion

The bacterial killing effect of ceftiofur depends upon the duration of the exposure at levels exceeding the MIC [50]. As described before, CCFA persisted approximately 2-fold longer than CHCL in the colon [17]. Nevertheless, the observed colonic concentrations of ceftiofur are unlikely to inhibit *C. jejuni,* as *Campylobacter* is intrinsically resistant to most cephalosporins [51], and the MIC of ceftiofur sodium (10–20 μg/mL) was at least 7.5-fold higher than the highest concentration of ceftiofur measured in the colon (1.32 μg/mL). Therefore, the stable prevalence of *C. jejuni*-positive samples in the CCFA group in week 1 (Figure 3) may be due to CCFA causing a steady inhibitory effect on other ceftiofur-susceptible microbes in the colon. On the other hand, the fluctuating prevalence of *C. jejuni*-positive samples in the CHCL group in week 1 (Figure 3) may be due to the three consecutive injections of this antibiotic, with concomitant changes in colonic CHCL concentrations and the associated effects on ceftiofur-susceptible microbes. The steady CCFA treatment effect was accompanied with a significantly higher estimated prevalence of *C. jejuni* and *Campylobacter* spp. in the CCFA group than in the CHCL group during week 1 (Figure 2).

Administration of CCFA resulted in a significant (*p* = 0.0108) decrease in the estimated prevalence of *Campylobacter* spp. from week 1 to week 2, while the estimated prevalence of *Campylobacter* spp. from steers receiving CHCL was also lower in week 2 than in week 1, though not significantly different. It is tempting to speculate that the reductions in the colonic drug concentrations led to *Campylobacter* spp. becoming outcompeted in the gut by the returning ceftiofur-susceptible bacteria. This is supported by previously-reported analyses of these samples which showed that in the CCFA group the mean concentration of *E. coli* decreased significantly within the first 48 h after treatment but increased slowly thereafter (over the subsequent 12 days of monitoring), while significant impacts on *E. coli* concentrations were not noted with CHCL treatment [17]. Metagenomic analysis has also revealed shifts in community composition of the dairy cow microbiome following ceftiofur treatment, specifically increases in *Bacterioidia* and decreases in *Actinobacteria* [16].

A previous study predicted that the intestinal ceftiofur equivalent concentration in 12-month steers after administration of CCFA would be approximately 5× higher than the detected peak colonic concentration of CCFA in this study (0.43 μg/mL) [52]. This deviation suggests the importance of determining the drug concentration at the site of action [17]. However, noticeable standard deviation may result from individual steer differences, as depicted in Figure 2.

The primary *Campylobacter* species isolated from cattle feces in this study was *C. jejuni.* The significantly higher estimated prevalence of *C. jejuni* at 42 °C may reflect the fact that this is the optimal incubation temperature for “thermophilic” campylobacters such as *C. jejuni*, while several other *Campylobacter* species, including *C. hyointestinalis* and *C. fetus*, prefer 37 °C [53,54]. *C. fetus* and *C. hyointestinalis* from the CCFA samples that were processed at both 37 °C and 42 °C were indeed primarily recovered from 37 °C, suggesting that including this temperature would be preferable to adequately capture *Campylobacter* species diversity.

*C. jejuni* is inherently resistant to cephalosporins, including ceftiofur [51,55,56,57], but the potential impact of ceftiofur treatment on MIC of this antimicrobial for *C. jejuni* has not been reported before. Our study suggested that the MIC of ceftiofur for *C. jejuni* was not impacted by treatment with either of the two ceftiofur formulations. In contrast, the ceftiofur MIC for *E. coli* from the steers in the CCFA and CHCL groups reached peak values at 96 h (37.16 and 51.05 μg/mL, respectively) and then returned to baseline at 14 days [17]. Singer et al. also found ceftiofur-resistant *E. coli* only during and immediately following ceftiofur treatment of dairy cattle [58], and a transient increase in ceftiofur-resistant *E. coli* was also noted upon treatment of feedlot cattle with CCFA [59].

The extent to which ceftiofur treatment may favor *C. jejuni* with specific AMR profiles, such as the TK profile observed here, remains poorly understood. In our study, animals in both trials appeared to be already colonized with *C. jejuni* TK before treatment but the prevalence of *C. jejuni* TK was higher post-CHCL treatment than post-CCFA treatment. Further studies with larger numbers of samples pre- and post-treatment will be needed to elucidate the potential impacts of ceftiofur treatment on specific genotypes and AMR profiles of *Campylobacter* spp. in cattle.

Among the ten *C. jejuni* STs that were identified in this study, two (STs 21 and 376) were reported previously. ST-21 has been commonly recovered from human samples [60,61,62,63], and in the PubMLST *Campylobacter* database ST-21 has been mainly reported in the UK (58.1%, 2367/4073), with 13.0% (531/4073) identified in the US. ST-376 isolates were mainly from the UK (*n* = 4) and have not been previously reported in the US. Our MLST analysis suggested that CCFA treatment may favor genotypic diversity. Several of the novel STs identified post-treatment in the CCFA animals were closely related to predominant STs identified in the study, suggesting that the diversification may have taken place within the animals during the study period. Factors that may favor such genetic diversification remain to be elucidated. Previous studies suggest that CCFA exerts greater impact on other microbiota in the gut than CHCL [17], which may also facilitate the propagation of *Campylobacter* and generation of diverse STs.

## 5. Conclusions

Studies investigating the impact of ceftiofur on *Campylobacter* in cattle have been lacking. Here, we found a transient but significant increase in the estimated prevalence of *Campylobacter* within one week after CCFA treatment of steers, as well as a trend for higher genotypic diversity of *C*. *jejuni* than observed upon CHCL treatment. Neither treatment was found to impact the MIC of ceftiofur and other tested antimicrobials for *C. jejuni* from the feces of the steers. Considering the lower likelihood of a temporary increase in *Campylobacter* prevalence and lower genotypic diversity, CHCL may be preferable. However, the higher prevalence of *C. jejuni* with concurrent resistance to tetracycline and kanamycin post-CHCL treatment than post-CCFA treatment merits further investigation. Considering the dynamic occurrence of *Campylobacter* in the individual steer, and the diverse *Campylobacter* species, STs, and AMR profiles, larger sample sizes and more pre-treatment sampling points would be needed for further assessments of the impacts of ceftiofur treatment of cattle on *Campylobacter* spp. prevalence, diversity, and AMR profiles. To enhance recovery of the diverse *Campylobacter* species and genotypes, such future studies may benefit from applying both 37 °C and 42 °C to isolate *Campylobacter* spp. from the bovine samples.

## Data Availability

All the data in this manuscript were in the Supplementary Tables.

## Supplementary Materials

The following are available online at https://www.mdpi.com/2076-2607/9/2/318/s1, Table S1: Isolate data, Table S2: *Campylobacter* spp. analyzed by multilocus sequence typing in this study, Table S3: Antimicrobial minimum inhibitory concentration (MIC) test of representative isolates.
