# Supplementary material for: Impact of Ceftiofur Administration in Steers on the Prevalence and Antimicrobial Resistance of Campylobacter spp
Source: Microorganisms. 2021 Feb 4;9(2):318. doi: 10.3390/microorganisms9020318 (PMC7913856; doi:10.3390/microorganisms9020318)
Supplement: Supplementary file 1 [file microorganisms-09-00318-s001.zip › microorganisms-1058745-S/Supplementary tables.microorganisms-1058745/Table S1.pdf]

Table S1: Isolate data

									CEF MIC		TET MIC					AMP MIC					NAL MIC		CIP MIC		ENR MIC		KAN MIC	
									Trial			Trial					Trial				Trial		Trial		Trial		Trial	
Isolate	Steer	Sampling point	Incubation temp. (°C)	Treatment	Sampling date	Species	AMR	ST; CC	1	2	Level	1	2	3	4	Level	1	2	3	4	1	2	1	2	1	2	1	2
Campylobacter negative	D1	0h	37	CCFA	5/18/16	Cneg	N.A	ND	ND	ND	ND	ND	ND	ND	ND	ND	ND	ND	ND	ND	ND	ND	ND	ND	ND	ND	ND	ND
D1-12h-37-A	D1	12h	37	CCFA	5/19/16	*Ch*	N	41 (Chy)	ND	ND	ND	ND	ND	ND	ND	ND	ND	ND	ND	ND	ND	ND	ND	ND	ND	ND	ND	ND
D1-12h-37-B	D1	12h	37	CCFA	5/19/16	*Ch* †	N	ND	ND	ND	ND	ND	ND	ND	ND	ND	ND	ND	ND	ND	ND	ND	ND	ND	ND	ND	ND	ND
D1-12h-37-C	D1	12h	37	CCFA	5/19/16	*Ch* †	N	ND	ND	ND	ND	ND	ND	ND	ND	ND	ND	ND	ND	ND	ND	ND	ND	ND	ND	ND	ND	ND
D1-12h-37-D	D1	12h	37	CCFA	5/19/16	*Ch* †	N	ND	ND	ND	ND	ND	ND	ND	ND	ND	ND	ND	ND	ND	ND	ND	ND	ND	ND	ND	ND	ND
D1-12h-37-E	D1	12h	37	CCFA	5/19/16	*Ch* †	N	ND	ND	ND	ND	ND	ND	ND	ND	ND	ND	ND	ND	ND	ND	ND	ND	ND	ND	ND	ND	ND
D1-12h-37-F	D1	12h	37	CCFA	5/19/16	*Ch* †	N	ND	ND	ND	ND	ND	ND	ND	ND	ND	ND	ND	ND	ND	ND	ND	ND	ND	ND	ND	ND	ND
D1-24h-37-A	D1	24h	37	CCFA	5/19/16	*Cf*	N	6 (Cfe)	ND	ND	ND	ND	ND	ND	ND	ND	ND	ND	ND	ND	ND	ND	ND	ND	ND	ND	ND	ND
D1-24h-37-E	D1	24h	37	CCFA	5/19/16	*Cf*	N	ND	ND	ND	ND	ND	ND	ND	ND	ND	ND	ND	ND	ND	ND	ND	ND	ND	ND	ND	ND	ND
D1-32h-37-A	D1	32h	37	CCFA	5/20/16	*Cj*	TK	**8221; 61**	ND	ND	ND	ND	ND	ND	ND	ND	ND	ND	ND	ND	ND	ND	ND	ND	ND	ND	ND	ND
D1-32h-37-C	D1	32h	37	CCFA	5/20/16	*Cj*	TK	ND	ND	ND	ND	ND	ND	ND	ND	ND	ND	ND	ND	ND	ND	ND	ND	ND	ND	ND	ND	ND
D1-48h-37-A	D1	48h	37	CCFA	5/20/16	*Cj*	TK	ND	ND	ND	ND	ND	ND	ND	ND	ND	ND	ND	ND	ND	ND	ND	ND	ND	ND	ND	ND	ND
D1-48h-37-B	D1	48h	37	CCFA	5/20/16	*Cj*	TK	ND	ND	ND	ND	ND	ND	ND	ND	ND	ND	ND	ND	ND	ND	ND	ND	ND	ND	ND	ND	ND
D1-48h-37-C	D1	48h	37	CCFA	5/20/16	*Cj*	TK	ND	ND	ND	ND	ND	ND	ND	ND	ND	ND	ND	ND	ND	ND	ND	ND	ND	ND	ND	ND	ND
D1-72h-37-A	D1	72h	37	CCFA	5/21/16	*Cj*	TK	ND	ND	ND	ND	ND	ND	ND	ND	ND	ND	ND	ND	ND	ND	ND	ND	ND	ND	ND	ND	ND
D1-72h-37-B	D1	72h	37	CCFA	5/21/16	*Cj*	TK	ND	ND	ND	ND	ND	ND	ND	ND	ND	ND	ND	ND	ND	ND	ND	ND	ND	ND	ND	ND	ND
D1-72h-37-C	D1	72h	37	CCFA	5/21/16	*Cj*	TK	ND	ND	ND	ND	ND	ND	ND	ND	ND	ND	ND	ND	ND	ND	ND	ND	ND	ND	ND	ND	ND
D1-72h-37-D	D1	72h	37	CCFA	5/21/16	*Cj*	TK	ND	ND	ND	ND	ND	ND	ND	ND	ND	ND	ND	ND	ND	ND	ND	ND	ND	ND	ND	ND	ND
D1-96h-37-A	D1	96h	37	CCFA	5/22/16	*Cj*	TK	ND	ND	ND	ND	ND	ND	ND	ND	ND	ND	ND	ND	ND	ND	ND	ND	ND	ND	ND	ND	ND
D1-96h-37-B	D1	96h	37	CCFA	5/22/16	*Cj*	TK	ND	ND	ND	ND	ND	ND	ND	ND	ND	ND	ND	ND	ND	ND	ND	ND	ND	ND	ND	ND	ND
D1-96h-37-C	D1	96h	37	CCFA	5/22/16	*Cj*	TK	ND	ND	ND	ND	ND	ND	ND	ND	ND	ND	ND	ND	ND	ND	ND	ND	ND	ND	ND	ND	ND
D1-96h-37-D	D1	96h	37	CCFA	5/22/16	*Cj*	TK	ND	ND	ND	ND	ND	ND	ND	ND	ND	ND	ND	ND	ND	ND	ND	ND	ND	ND	ND	ND	ND
D1-5d-37-A	D1	5d	37	CCFA	5/23/16	*Cj*	TK	ND	ND	ND	ND	ND	ND	ND	ND	ND	ND	ND	ND	ND	ND	ND	ND	ND	ND	ND	ND	ND
D1-5d-37-B	D1	5d	37	CCFA	5/23/16	*Cj*	TK	ND	ND	ND	ND	ND	ND	ND	ND	ND	ND	ND	ND	ND	ND	ND	ND	ND	ND	ND	ND	ND
D1-5d-37-C	D1	5d	37	CCFA	5/23/16	*Cj*	TK	ND	ND	ND	ND	ND	ND	ND	ND	ND	ND	ND	ND	ND	ND	ND	ND	ND	ND	ND	ND	ND
D1-5d-37-D	D1	5d	37	CCFA	5/23/16	*Cj*	TK	ND	ND	ND	ND	ND	ND	ND	ND	ND	ND	ND	ND	ND	ND	ND	ND	ND	ND	ND	ND	ND
D1-6d-37-A	D1	6d	37	CCFA	5/24/16	*Cj*	TK	ND	ND	ND	ND	ND	ND	ND	ND	ND	ND	ND	ND	ND	ND	ND	ND	ND	ND	ND	ND	ND
D1-6d-37-B	D1	6d	37	CCFA	5/24/16	*Cj*	TK	ND	ND	ND	ND	ND	ND	ND	ND	ND	ND	ND	ND	ND	ND	ND	ND	ND	ND	ND	ND	ND
D1-6d-37-D	D1	6d	37	CCFA	5/24/16	*Cj*	TK	ND	ND	ND	ND	ND	ND	ND	ND	ND	ND	ND	ND	ND	ND	ND	ND	ND	ND	ND	ND	ND
D1-7d-37-A	D1	7d	37	CCFA	5/25/16	*Cj*	TK	ND	ND	ND	ND	ND	ND	ND	ND	ND	ND	ND	ND	ND	ND	ND	ND	ND	ND	ND	ND	ND
D1-7d-37-B	D1	7d	37	CCFA	5/25/16	*Cj*	TK	ND	ND	ND	ND	ND	ND	ND	ND	ND	ND	ND	ND	ND	ND	ND	ND	ND	ND	ND	ND	ND
D1-7d-37-C	D1	7d	37	CCFA	5/25/16	*Cj*	TK	ND	ND	ND	ND	ND	ND	ND	ND	ND	ND	ND	ND	ND	ND	ND	ND	ND	ND	ND	ND	ND
D1-7d-37-D	D1	7d	37	CCFA	5/25/16	*Cj*	TK	ND	ND	ND	ND	ND	ND	ND	ND	ND	ND	ND	ND	ND	ND	ND	ND	ND	ND	ND	ND	ND
Campylobacter negative	D1	8d	37	CCFA	5/26/16	Cneg	N.A	ND	ND	ND	ND	ND	ND	ND	ND	ND	ND	ND	ND	ND	ND	ND	ND	ND	ND	ND	ND	ND
D1-21d-37-A	D1	21d	37	CCFA	6/8/16	*Cj*	PS	**8566; 21**	ND	ND	ND	ND	ND	ND	ND	ND	ND	ND	ND	ND	ND	ND	ND	ND	ND	ND	ND	ND
D1-21d-37-B	D1	21d	37	CCFA	6/8/16	*Cj*	TK	**8565; 21**	ND	ND	ND	ND	ND	ND	ND	ND	ND	ND	ND	ND	ND	ND	ND	ND	ND	ND	ND	ND
D1-21d-37-C	D1	21d	37	CCFA	6/8/16	*Cj*	PS	ND	ND	ND	ND	ND	ND	ND	ND	ND	ND	ND	ND	ND	ND	ND	ND	ND	ND	ND	ND	ND
D1-21d-37-D	D1	21d	37	CCFA	6/8/16	*Cj*	PS	ND	ND	ND	ND	ND	ND	ND	ND	ND	ND	ND	ND	ND	ND	ND	ND	ND	ND	ND	ND	ND
Campylobacter negative	D1	0h	42	CCFA	5/18/16	Cneg	N.A	ND	ND	ND	ND	ND	ND	ND	ND	ND	ND	ND	ND	ND	ND	ND	ND	ND	ND	ND	ND	ND
Campylobacter negative	D1	12h	42	CCFA	5/19/16	Cneg	N.A	ND	ND	ND	ND	ND	ND	ND	ND	ND	ND	ND	ND	ND	ND	ND	ND	ND	ND	ND	ND	ND
D1-24h-42-A	D1	24h	42	CCFA	5/19/16	*Cj*	TK	**8221; 61**	ND	ND	ND	ND	ND	ND	ND	ND	ND	ND	ND	ND	ND	ND	ND	ND	ND	ND	ND	ND
D1-24h-42-C	D1	24h	42	CCFA	5/19/16	*Cj*	TK	ND	ND	ND	ND	ND	ND	ND	ND	ND	ND	ND	ND	ND	ND	ND	ND	ND	ND	ND	ND	ND
D1-24h-42-D	D1	24h	42	CCFA	5/19/16	*Cj*	TK	ND	ND	ND	ND	ND	ND	ND	ND	ND	ND	ND	ND	ND	ND	ND	ND	ND	ND	ND	ND	ND
D1-24h-42-E	D1	24h	42	CCFA	5/19/16	*Cj*	TK	ND	ND	ND	ND	ND	ND	ND	ND	ND	ND	ND	ND	ND	ND	ND	ND	ND	ND	ND	ND	ND

Table S1 continued

									CEF MIC		TET					AMP					NAL MIC		CIP MIC		ENR MIC		KAN MIC	
									Trial			MIC					MIC				Trial		Trial		Trial		Trial	
												Trial					Trial											
Isolate	Steer	Sampling time point	Incubation temp. (°C)	Treatment	Sampling date	Species	AMR	ST; CC	1	2	Level	1	2	3	4	Level	1	2	3	4	1	2	1	2	1	2	1	2
D1-32h-42-B	D1	32h	42	CCFA	5/20/16	*Cj*	TK	ND	ND	ND	ND	ND	ND	ND	ND	ND	ND	ND	ND	ND	ND	ND	ND	ND	ND	ND	ND	ND
D1-32h-42-C	D1	32h	42	CCFA	5/20/16	*Cj*	TK	ND	ND	ND	ND	ND	ND	ND	ND	ND	ND	ND	ND	ND	ND	ND	ND	ND	ND	ND	ND	ND
D1-32h-42-D	D1	32h	42	CCFA	5/20/16	*Cj*	TK	ND	ND	ND	ND	ND	ND	ND	ND	ND	ND	ND	ND	ND	ND	ND	ND	ND	ND	ND	ND	ND
D1-48h-42-A	D1	48h	42	CCFA	5/20/16	*Cj*	TK	ND	ND	ND	ND	ND	ND	ND	ND	ND	ND	ND	ND	ND	ND	ND	ND	ND	ND	ND	ND	ND
D1-48h-42-B	D1	48h	42	CCFA	5/20/16	*Cj*	TK	ND	ND	ND	ND	ND	ND	ND	ND	ND	ND	ND	ND	ND	ND	ND	ND	ND	ND	ND	ND	ND
D1-48h-42-C	D1	48h	42	CCFA	5/20/16	*Cj*	TK	ND	ND	ND	ND	ND	ND	ND	ND	ND	ND	ND	ND	ND	ND	ND	ND	ND	ND	ND	ND	ND
D1-48h-42-D	D1	48h	42	CCFA	5/20/16	*Cj*	TK	ND	ND	ND	ND	ND	ND	ND	ND	ND	ND	ND	ND	ND	ND	ND	ND	ND	ND	ND	ND	ND
D1-72h-42-A	D1	72h	42	CCFA	5/21/16	*Cj*	TK	ND	ND	ND	ND	ND	ND	ND	ND	ND	ND	ND	ND	ND	ND	ND	ND	ND	ND	ND	ND	ND
D1-72h-42-B	D1	72h	42	CCFA	5/21/16	*Cj*	TK	ND	ND	ND	ND	ND	ND	ND	ND	ND	ND	ND	ND	ND	ND	ND	ND	ND	ND	ND	ND	ND
D1-72h-42-C	D1	72h	42	CCFA	5/21/16	*Cj*	TK	ND	ND	ND	ND	ND	ND	ND	ND	ND	ND	ND	ND	ND	ND	ND	ND	ND	ND	ND	ND	ND
D1-72h-42-D	D1	72h	42	CCFA	5/21/16	*Cj*	TK	ND	ND	ND	ND	ND	ND	ND	ND	ND	ND	ND	ND	ND	ND	ND	ND	ND	ND	ND	ND	ND
D1-96h-42-A	D1	96h	42	CCFA	5/22/16	*Cj*	TK	ND	ND	ND	ND	ND	ND	ND	ND	ND	ND	ND	ND	ND	ND	ND	ND	ND	ND	ND	ND	ND
D1-96h-42-B	D1	96h	42	CCFA	5/22/16	*Cj*	TK	ND	ND	ND	ND	ND	ND	ND	ND	ND	ND	ND	ND	ND	ND	ND	ND	ND	ND	ND	ND	ND
D1-96h-42-C	D1	96h	42	CCFA	5/22/16	*Cj*	TK	ND	ND	ND	ND	ND	ND	ND	ND	ND	ND	ND	ND	ND	ND	ND	ND	ND	ND	ND	ND	ND
D1-96h-42-D	D1	96h	42	CCFA	5/22/16	*Cj*	TK	ND	ND	ND	ND	ND	ND	ND	ND	ND	ND	ND	ND	ND	ND	ND	ND	ND	ND	ND	ND	ND
D1-5d-42-A	D1	5d	42	CCFA	5/23/16	*Cj*	TK	ND	ND	ND	ND	ND	ND	ND	ND	ND	ND	ND	ND	ND	ND	ND	ND	ND	ND	ND	ND	ND
D1-5d-42-B	D1	5d	42	CCFA	5/23/16	*Cj*	TK	ND	ND	ND	ND	ND	ND	ND	ND	ND	ND	ND	ND	ND	ND	ND	ND	ND	ND	ND	ND	ND
D1-5d-42-C	D1	5d	42	CCFA	5/23/16	*Cj*	TK	ND	ND	ND	ND	ND	ND	ND	ND	ND	ND	ND	ND	ND	ND	ND	ND	ND	ND	ND	ND	ND
D1-5d-42-D	D1	5d	42	CCFA	5/23/16	*Cj*	TK	ND	ND	ND	ND	ND	ND	ND	ND	ND	ND	ND	ND	ND	ND	ND	ND	ND	ND	ND	ND	ND
D1-6d-42-B	D1	6d	42	CCFA	5/24/16	*Cj*	TK	ND	ND	ND	ND	ND	ND	ND	ND	ND	ND	ND	ND	ND	ND	ND	ND	ND	ND	ND	ND	ND
D1-6d-42-C	D1	6d	42	CCFA	5/24/16	*Cj*	TK	ND	ND	ND	ND	ND	ND	ND	ND	ND	ND	ND	ND	ND	ND	ND	ND	ND	ND	ND	ND	ND
D1-6d-42-D	D1	6d	42	CCFA	5/24/16	*Cj*	TK	ND	ND	ND	ND	ND	ND	ND	ND	ND	ND	ND	ND	ND	ND	ND	ND	ND	ND	ND	ND	ND
D1-7d-42-A	D1	7d	42	CCFA	5/25/16	*Cj*	TK	ND	ND	ND	ND	ND	ND	ND	ND	ND	ND	ND	ND	ND	ND	ND	ND	ND	ND	ND	ND	ND
D1-7d-42-B	D1	7d	42	CCFA	5/25/16	*Cj*	TK	ND	ND	ND	ND	ND	ND	ND	ND	ND	ND	ND	ND	ND	ND	ND	ND	ND	ND	ND	ND	ND
D1-8d-42-A	D1	8d	42	CCFA	5/26/16	*Cj*	TK	**8221; 61**	ND	ND	ND	ND	ND	ND	ND	ND	ND	ND	ND	ND	ND	ND	ND	ND	ND	ND	ND	ND
D1-8d-42-B	D1	8d	42	CCFA	5/26/16	*Cj*	TK	ND	ND	ND	ND	ND	ND	ND	ND	ND	ND	ND	ND	ND	ND	ND	ND	ND	ND	ND	ND	ND
D1-8d-42-D	D1	8d	42	CCFA	5/26/16	*Cj*	TK	ND	ND	ND	ND	ND	ND	ND	ND	ND	ND	ND	ND	ND	ND	ND	ND	ND	ND	ND	ND	ND
Campylobacter negative	D1	14d	42	CCFA	6/1/16	Cneg	N.A	ND	ND	ND	ND	ND	ND	ND	ND	ND	ND	ND	ND	ND	ND	ND	ND	ND	ND	ND	ND	ND
D2-0h-37-A	D2	0h	37	CCFA	5/18/16	*Cj*	TK	ND	ND	ND	ND	ND	ND	ND	ND	ND	ND	ND	ND	ND	ND	ND	ND	ND	ND	ND	ND	ND
D2-0h-37-B	D2	0h	37	CCFA	5/18/16	*Cj*	TK	ND	ND	ND	ND	ND	ND	ND	ND	ND	ND	ND	ND	ND	ND	ND	ND	ND	ND	ND	ND	ND
D2-0h-37-C	D2	0h	37	CCFA	5/18/16	*Cj*	TK	**8567; 21**	ND	ND	ND	ND	ND	ND	ND	ND	ND	ND	ND	ND	ND	ND	ND	ND	ND	ND	ND	ND
D2-0h-37-D	D2	0h	37	CCFA	5/18/16	*Cj*	TK	**8567; 21**	10	10	B	128	128	64	64	B	10	10	32	32	4	4	1	1	1	1	>256	>256
D2-0h-37-E	D2	0h	37	CCFA	5/18/16	*Cj*	TK	ND	ND	ND	ND	ND	ND	ND	ND	ND	ND	ND	ND	ND	ND	ND	ND	ND	ND	ND	ND	ND
D2-0h-37-F	D2	0h	37	CCFA	5/18/16	*Cj*	TK	ND	ND	ND	ND	ND	ND	ND	ND	ND	ND	ND	ND	ND	ND	ND	ND	ND	ND	ND	ND	ND
D2-12h-37-C	D2	12h	37	CCFA	5/19/16	*Ch*	TN	6 (Chy)	ND	ND	ND	ND	ND	ND	ND	ND	ND	ND	ND	ND	ND	ND	ND	ND	ND	ND	ND	ND
D2-12h-37-E	D2	12h	37	CCFA	5/19/16	*Ch*†	TN	ND	ND	ND	ND	ND	ND	ND	ND	ND	ND	ND	ND	ND	ND	ND	ND	ND	ND	ND	ND	ND
D2-24h-37-D	D2	24h	37	CCFA	5/19/16	*Ch*	TN	6 (Chy)	ND	ND	ND	ND	ND	ND	ND	ND	ND	ND	ND	ND	ND	ND	ND	ND	ND	ND	ND	ND
D2-32h-37-A	D2	32h	37	CCFA	5/20/16	*Cj*	TK	ND	10	10	ND	128	128	ND	ND	ND	10	10	ND	ND	4	4	1	1	1	1	>256	>256
D2-32h-37-B	D2	32h	37	CCFA	5/20/16	*Cj*	TK	ND	ND	ND	ND	ND	ND	ND	ND	ND	ND	ND	ND	ND	ND	ND	ND	ND	ND	ND	ND	ND
D2-32h-37-C	D2	32h	37	CCFA	5/20/16	*Cj*	TK	ND	ND	ND	ND	ND	ND	ND	ND	ND	ND	ND	ND	ND	ND	ND	ND	ND	ND	ND	ND	ND
D2-32h-37-D	D2	32h	37	CCFA	5/20/16	*Cj*	TK	ND	ND	ND	ND	ND	ND	ND	ND	ND	ND	ND	ND	ND	ND	ND	ND	ND	ND	ND	ND	ND
D2-48h-37-A	D2	48h	37	CCFA	5/20/16	*Cj*	TK	ND	ND	ND	ND	ND	ND	ND	ND	ND	ND	ND	ND	ND	ND	ND	ND	ND	ND	ND	ND	ND
D2-48h-37-B	D2	48h	37	CCFA	5/20/16	*Cj*	TK	ND	ND	ND	ND	ND	ND	ND	ND	ND	ND	ND	ND	ND	ND	ND	ND	ND	ND	ND	ND	ND
D2-48h-37-C	D2	48h	37	CCFA	5/20/16	*Cj*	TK	ND	ND	ND	ND	ND	ND	ND	ND	ND	ND	ND	ND	ND	ND	ND	ND	ND	ND	ND	ND	ND

Table S1 continued

									CEF MIC		TET					AMP					NAL MIC		CIP MIC		ENR MIC		KAN MIC	
									Trial			MIC Trial					MIC Trial				Trial		Trial		Trial		Trial	
Isolate	Steer	Sampling time point	Incubation temp. (°C)	Treatment	Sampling date	Species	AMR	ST; CC	1	2	Level	1	2	3	4	Level	1	2	3	4	1	2	1	2	1	2	1	2
D2-48h-37-D	D2	48h	37	CCFA	5/20/16	*Cj*	TK	ND	ND	ND	ND	ND	ND	ND	ND	ND	ND	ND	ND	ND	ND	ND	ND	ND	ND	ND	ND	ND
D2-72h-37-A	D2	72h	37	CCFA	5/21/16	*Cj*	TK	**8567; 21**	10	10	B	128	128	64	64	B	10	10	32	32	4	4	1	1	1	1	>256	>256
D2-72h-37-B	D2	72h	37	CCFA	5/21/16	*Cj*	TK	ND	ND	ND	ND	ND	ND	ND	ND	ND	ND	ND	ND	ND	ND	ND	ND	ND	ND	ND	ND	ND
D2-72h-37-D	D2	72h	37	CCFA	5/21/16	*Cj*	TK	ND	ND	ND	ND	ND	ND	ND	ND	ND	ND	ND	ND	ND	ND	ND	ND	ND	ND	ND	ND	ND
D2-96h-37-A	D2	96h	37	CCFA	5/22/16	*Cj*	TK	ND	ND	ND	ND	ND	ND	ND	ND	ND	ND	ND	ND	ND	ND	ND	ND	ND	ND	ND	ND	ND
D2-96h-37-B	D2	96h	37	CCFA	5/22/16	*Cj*	TK	ND	ND	ND	ND	ND	ND	ND	ND	ND	ND	ND	ND	ND	ND	ND	ND	ND	ND	ND	ND	ND
D2-96h-37-C	D2	96h	37	CCFA	5/22/16	*Cj*	TK	ND	ND	ND	ND	ND	ND	ND	ND	ND	ND	ND	ND	ND	ND	ND	ND	ND	ND	ND	ND	ND
Campylobacter negative	D2	5d	37	CCFA	5/23/16	Cneg	N.A	ND	ND	ND	ND	ND	ND	ND	ND	ND	ND	ND	ND	ND	ND	ND	ND	ND	ND	ND	ND	ND
Campylobacter negative	D2	6d	37	CCFA	5/24/16	Cneg	N.A	ND	ND	ND	ND	ND	ND	ND	ND	ND	ND	ND	ND	ND	ND	ND	ND	ND	ND	ND	ND	ND
D2-7d-37-A	D2	7d	37	CCFA	5/25/16	*Cj*	TK	**8567; 21**	10	10	B	128	128	64	64	B	10	10	32	32	4	4	1	1	1	1	>256	>256
D2-7d-37-B	D2	7d	37	CCFA	5/25/16	*Cj*	TK	ND	ND	ND	ND	ND	ND	ND	ND	ND	ND	ND	ND	ND	ND	ND	ND	ND	ND	ND	ND	ND
D2-7d-37-C	D2	7d	37	CCFA	5/25/16	*Cj*	TK	ND	ND	ND	ND	ND	ND	ND	ND	ND	ND	ND	ND	ND	ND	ND	ND	ND	ND	ND	ND	ND
D2-7d-37-D	D2	7d	37	CCFA	5/25/16	*Cj*	TK	ND	ND	ND	ND	ND	ND	ND	ND	ND	ND	ND	ND	ND	ND	ND	ND	ND	ND	ND	ND	ND
Campylobacter negative	D2	8d	37	CCFA	5/26/16	Cneg	N.A	ND	ND	ND	ND	ND	ND	ND	ND	ND	ND	ND	ND	ND	ND	ND	ND	ND	ND	ND	ND	ND
Campylobacter negative	D2	21d	37	CCFA	6/8/16	Cneg	N.A	ND	ND	ND	ND	ND	ND	ND	ND	ND	ND	ND	ND	ND	ND	ND	ND	ND	ND	ND	ND	ND
D2-0h-42-A	D2	0h	42	CCFA	5/18/16	*Cj*	TK	**8567; 21**	10	10	B	128	128	64	64	B	16	16	32	32	4	4	1	1	1	1	>256	>256
D2-0h-42-B	D2	0h	42	CCFA	5/18/16	*Cj*	TK	ND	ND	ND	ND	ND	ND	ND	ND	ND	ND	ND	ND	ND	ND	ND	ND	ND	ND	ND	ND	ND
D2-0h-42-C	D2	0h	42	CCFA	5/18/16	*Cj*	TK	ND	ND	ND	ND	ND	ND	ND	ND	ND	ND	ND	ND	ND	ND	ND	ND	ND	ND	ND	ND	ND
D2-0h-42-D	D2	0h	42	CCFA	5/18/16	*Cj*	TK	ND	ND	ND	ND	ND	ND	ND	ND	ND	ND	ND	ND	ND	ND	ND	ND	ND	ND	ND	ND	ND
D2-0h-42-E	D2	0h	42	CCFA	5/18/16	*Cj*	TK	ND	ND	ND	ND	ND	ND	ND	ND	ND	ND	ND	ND	ND	ND	ND	ND	ND	ND	ND	ND	ND
D2-0h-42-F	D2	0h	42	CCFA	5/18/16	*Cj*	TK	ND	ND	ND	ND	ND	ND	ND	ND	ND	ND	ND	ND	ND	ND	ND	ND	ND	ND	ND	ND	ND
D2-12h-42-A	D2	12h	42	CCFA	5/19/16	*Cj*	TK	ND	ND	ND	ND	ND	ND	ND	ND	ND	ND	ND	ND	ND	ND	ND	ND	ND	ND	ND	ND	ND
D2-12h-42-B	D2	12h	42	CCFA	5/19/16	*Cj*	TK	ND	ND	ND	ND	ND	ND	ND	ND	ND	ND	ND	ND	ND	ND	ND	ND	ND	ND	ND	ND	ND
D2-12h-42-D	D2	12h	42	CCFA	5/19/16	*Cj*	TK	ND	ND	ND	ND	ND	ND	ND	ND	ND	ND	ND	ND	ND	ND	ND	ND	ND	ND	ND	ND	ND
D2-12h-42-E	D2	12h	42	CCFA	5/19/16	*Cj*	TK	ND	ND	ND	ND	ND	ND	ND	ND	ND	ND	ND	ND	ND	ND	ND	ND	ND	ND	ND	ND	ND
D2-12h-42-F	D2	12h	42	CCFA	5/19/16	*Cj*	TK	ND	ND	ND	ND	ND	ND	ND	ND	ND	ND	ND	ND	ND	ND	ND	ND	ND	ND	ND	ND	ND
Campylobacter negative	D2	24h	42	CCFA	5/19/16	Cneg	N.A	ND	ND	ND	ND	ND	ND	ND	ND	ND	ND	ND	ND	ND	ND	ND	ND	ND	ND	ND	ND	ND
D2-32h-42-A	D2	32h	42	CCFA	5/20/16	*Cj*	TK	ND	10	10	ND	128	128	ND	ND	ND	16	16	ND	ND	4	4	1	1	1	1	>256	>256
D2-32h-42-B	D2	32h	42	CCFA	5/20/16	*Cj*	TK	ND	ND	ND	ND	ND	ND	ND	ND	ND	ND	ND	ND	ND	ND	ND	ND	ND	ND	ND	ND	ND
D2-32h-42-D	D2	32h	42	CCFA	5/20/16	*Cj*	TK	ND	ND	ND	ND	ND	ND	ND	ND	ND	ND	ND	ND	ND	ND	ND	ND	ND	ND	ND	ND	ND
D2-48h-42-A	D2	48h	42	CCFA	5/20/16	*Cj*	TK	ND	ND	ND	ND	ND	ND	ND	ND	ND	ND	ND	ND	ND	ND	ND	ND	ND	ND	ND	ND	ND
D2-48h-42-B	D2	48h	42	CCFA	5/20/16	*Cj*	TK	ND	ND	ND	ND	ND	ND	ND	ND	ND	ND	ND	ND	ND	ND	ND	ND	ND	ND	ND	ND	ND
D2-48h-42-C	D2	48h	42	CCFA	5/20/16	*Cj*	TK	ND	ND	ND	ND	ND	ND	ND	ND	ND	ND	ND	ND	ND	ND	ND	ND	ND	ND	ND	ND	ND
D2-48h-42-D	D2	48h	42	CCFA	5/20/16	*Cj*	TK	ND	ND	ND	ND	ND	ND	ND	ND	ND	ND	ND	ND	ND	ND	ND	ND	ND	ND	ND	ND	ND
D2-72h-42-A	D2	72h	42	CCFA	5/21/16	*Cj*	TK	ND	ND	ND	ND	ND	ND	ND	ND	ND	ND	ND	ND	ND	ND	ND	ND	ND	ND	ND	ND	ND
D2-72h-42-C	D2	72h	42	CCFA	5/21/16	*Cj*	TK	**8567; 21**	10	10	B	128	128	64	64	B	16	16	32	32	4	4	1	1	1	1	>256	>256
D2-72h-42-D	D2	72h	42	CCFA	5/21/16	*Cj*	TK	ND	ND	ND	ND	ND	ND	ND	ND	ND	ND	ND	ND	ND	ND	ND	ND	ND	ND	ND	ND	ND
D2-96h-42-A	D2	96h	42	CCFA	5/22/16	*Cj*	TK	ND	ND	ND	ND	ND	ND	ND	ND	ND	ND	ND	ND	ND	ND	ND	ND	ND	ND	ND	ND	ND
D2-96h-42-B	D2	96h	42	CCFA	5/22/16	*Cj*	TK	ND	ND	ND	ND	ND	ND	ND	ND	ND	ND	ND	ND	ND	ND	ND	ND	ND	ND	ND	ND	ND
D2-96h-42-C	D2	96h	42	CCFA	5/22/16	*Cj*	TK	ND	ND	ND	ND	ND	ND	ND	ND	ND	ND	ND	ND	ND	ND	ND	ND	ND	ND	ND	ND	ND
D2-96h-42-D	D2	96h	42	CCFA	5/22/16	*Cj*	TK	ND	ND	ND	ND	ND	ND	ND	ND	ND	ND	ND	ND	ND	ND	ND	ND	ND	ND	ND	ND	ND
D2-5d-42-B	D2	5d	42	CCFA	5/23/16	*Cj*	TK	ND	ND	ND	ND	ND	ND	ND	ND	ND	ND	ND	ND	ND	ND	ND	ND	ND	ND	ND	ND	ND
D2-5d-42-C	D2	5d	42	CCFA	5/23/16	*Cj*	TK	ND	ND	ND	ND	ND	ND	ND	ND	ND	ND	ND	ND	ND	ND	ND	ND	ND	ND	ND	ND	ND
D2-5d-42-D	D2	5d	42	CCFA	5/23/16	*Cj*	TK	**8567; 21**	ND	ND	ND	ND	ND	ND	ND	ND	ND	ND	ND	ND	ND	ND	ND	ND	ND	ND	ND	ND

Table S1 continued

Isolate	Steer	Sampling time point	Incubation temp. (°C)	Treatment	Sampling date	Species	AMR	ST; CC	CEF MIC Trial		TET					AMP					NAL MIC Trial		CIP MIC Trial		ENR MIC Trial		KAN MIC Trial	
									1	2	Level	MIC Trial 1	2	3	4	Level	MIC Trial 1	2	3	4	1	2	1	2	1	2	1	2
Campylobacter negative	D2	6d	42	CCFA	5/24/16	Cneg	N.A	ND	ND	ND	ND	ND	ND	ND	ND	ND	ND	ND	ND	ND	ND	ND	ND	ND	ND	ND	ND	ND
D2-7d-42-A	D2	7d	42	CCFA	5/25/16	*Cj*	TK	376*; 21	10	10	B	128	128	64	64	B	8	8	32	32	4	4	1	1	1	1	>256	>256
D2-7d-42-B	D2	7d	42	CCFA	5/25/16	*Cj*	TK	ND	10	10	ND	128	128	ND	ND	ND	8	8	ND	ND	4	4	1	1	1	1	>256	>256
D2-7d-42-C	D2	7d	42	CCFA	5/25/16	*Cj*	TK	ND	ND	ND	ND	ND	ND	ND	ND	ND	ND	ND	ND	ND	ND	ND	ND	ND	ND	ND	ND	ND
D2-7d-42-D	D2	7d	42	CCFA	5/25/16	*Cj*	TK	ND	ND	ND	ND	ND	ND	ND	ND	ND	ND	ND	ND	ND	ND	ND	ND	ND	ND	ND	ND	ND
Campylobacter negative	D2	8d	42	CCFA	5/26/16	Cneg	N.A	ND	ND	ND	ND	ND	ND	ND	ND	ND	ND	ND	ND	ND	ND	ND	ND	ND	ND	ND	ND	ND
Campylobacter negative	D2	14d	42	CCFA	6/1/16	Cneg	N.A	ND	ND	ND	ND	ND	ND	ND	ND	ND	ND	ND	ND	ND	ND	ND	ND	ND	ND	ND	ND	ND
D3-0h-37-A	D3	0h	37	CCFA	5/18/16	*Cj*	TK	**8221; 61**	20	20	A	>128	>128	128	128	A	30	30	>32	>32	8	8	1	1	1	1	>256	>256
D3-0h-37-D	D3	0h	37	CCFA	5/18/16	*Cj*	TK	ND	ND	ND	ND	ND	ND	ND	ND	ND	ND	ND	ND	ND	ND	ND	ND	ND	ND	ND	ND	ND
D3-0h-37-F	D3	0h	37	CCFA	5/18/16	*Cj*	TK	ND	ND	ND	ND	ND	ND	ND	ND	ND	ND	ND	ND	ND	ND	ND	ND	ND	ND	ND	ND	ND
D3-12h-37-A	D3	12h	37	CCFA	5/19/16	*Cj*	PS	21; 21	ND	ND	ND	ND	ND	ND	ND	ND	ND	ND	ND	ND	ND	ND	ND	ND	ND	ND	ND	ND
D3-12h-37-B	D3	12h	37	CCFA	5/19/16	*Cj*	PS	ND	ND	ND	ND	ND	ND	ND	ND	ND	ND	ND	ND	ND	ND	ND	ND	ND	ND	ND	ND	ND
D3-12h-37-C	D3	12h	37	CCFA	5/19/16	*Cj*	PS	ND	ND	ND	ND	ND	ND	ND	ND	ND	ND	ND	ND	ND	ND	ND	ND	ND	ND	ND	ND	ND
D3-12h-37-D	D3	12h	37	CCFA	5/19/16	*Cj*	PS	ND	ND	ND	ND	ND	ND	ND	ND	ND	ND	ND	ND	ND	ND	ND	ND	ND	ND	ND	ND	ND
D3-12h-37-E	D3	12h	37	CCFA	5/19/16	*Cj*	PS	ND	ND	ND	ND	ND	ND	ND	ND	ND	ND	ND	ND	ND	ND	ND	ND	ND	ND	ND	ND	ND
D3-12h-37-F	D3	12h	37	CCFA	5/19/16	*Cj*	TK	ND	20	20	ND	>128	>128	ND	ND	ND	30	30	ND	ND	8	8	1	1	1	1	>256	>256
D3-24h-37-A	D3	24h	37	CCFA	5/19/16	*Cj*	TK	ND	20	20	ND	>128	>128	ND	ND	ND	30	30	ND	ND	8	8	1	1	1	1	>256	>256
D3-24h-37-B	D3	24h	37	CCFA	5/19/16	*Cj*	PS	21; 21	ND	ND	ND	ND	ND	ND	ND	ND	ND	ND	ND	ND	ND	ND	ND	ND	ND	ND	ND	ND
D3-24h-37-C	D3	24h	37	CCFA	5/19/16	*Cj*	TK	ND	ND	ND	ND	ND	ND	ND	ND	ND	ND	ND	ND	ND	ND	ND	ND	ND	ND	ND	ND	ND
D3-24h-37-D	D3	24h	37	CCFA	5/19/16	*Cj*	TK	ND	ND	ND	ND	ND	ND	ND	ND	ND	ND	ND	ND	ND	ND	ND	ND	ND	ND	ND	ND	ND
D3-24h-37-E	D3	24h	37	CCFA	5/19/16	*Cj*	TK	ND	ND	ND	ND	ND	ND	ND	ND	ND	ND	ND	ND	ND	ND	ND	ND	ND	ND	ND	ND	ND
D3-24h-37-F	D3	24h	37	CCFA	5/19/16	*Cj*	TK	ND	ND	ND	ND	ND	ND	ND	ND	ND	ND	ND	ND	ND	ND	ND	ND	ND	ND	ND	ND	ND
Campylobacter negative	D3	32h	37	CCFA	5/20/16	Cneg	N.A	ND	ND	ND	ND	ND	ND	ND	ND	ND	ND	ND	ND	ND	ND	ND	ND	ND	ND	ND	ND	ND
Campylobacter negative	D3	48h	37	CCFA	5/20/16	Cneg	N.A	ND	ND	ND	ND	ND	ND	ND	ND	ND	ND	ND	ND	ND	ND	ND	ND	ND	ND	ND	ND	ND
Campylobacter negative	D3	72h	37	CCFA	5/21/16	Cneg	N.A	ND	ND	ND	ND	ND	ND	ND	ND	ND	ND	ND	ND	ND	ND	ND	ND	ND	ND	ND	ND	ND
Campylobacter negative	D3	96h	37	CCFA	5/22/16	Cneg	N.A	ND	ND	ND	ND	ND	ND	ND	ND	ND	ND	ND	ND	ND	ND	ND	ND	ND	ND	ND	ND	ND
D3-5d-37-A	D3	5d	37	CCFA	5/23/16	*Cj*	TK	**8221; 61**	20	20	A	>128	>128	128	128	A	30	30	>32	>32	8	8	1	1	1	1	>256	>256
D3-5d-37-B	D3	5d	37	CCFA	5/23/16	*Cj*	TK	ND	ND	ND	ND	ND	ND	ND	ND	ND	ND	ND	ND	ND	ND	ND	ND	ND	ND	ND	ND	ND
D3-5d-37-C	D3	5d	37	CCFA	5/23/16	*Cj*	TK	ND	ND	ND	ND	ND	ND	ND	ND	ND	ND	ND	ND	ND	ND	ND	ND	ND	ND	ND	ND	ND
D3-5d-37-D	D3	5d	37	CCFA	5/23/16	*Cj*	TK	ND	ND	ND	ND	ND	ND	ND	ND	ND	ND	ND	ND	ND	ND	ND	ND	ND	ND	ND	ND	ND
D3-5d-37-E	D3	5d	37	CCFA	5/23/16	*Cj*	TK	ND	ND	ND	ND	ND	ND	ND	ND	ND	ND	ND	ND	ND	ND	ND	ND	ND	ND	ND	ND	ND
D3-5d-37-F	D3	5d	37	CCFA	5/23/16	*Cj*	TK	ND	ND	ND	ND	ND	ND	ND	ND	ND	ND	ND	ND	ND	ND	ND	ND	ND	ND	ND	ND	ND
Campylobacter negative	D3	6d	37	CCFA	5/24/16	Cneg	N.A	ND	ND	ND	ND	ND	ND	ND	ND	ND	ND	ND	ND	ND	ND	ND	ND	ND	ND	ND	ND	ND
Campylobacter negative	D3	7d	37	CCFA	5/25/16	Cneg	N.A	ND	ND	ND	ND	ND	ND	ND	ND	ND	ND	ND	ND	ND	ND	ND	ND	ND	ND	ND	ND	ND
Campylobacter negative	D3	8d	37	CCFA	5/26/16	Cneg	N.A	ND	ND	ND	ND	ND	ND	ND	ND	ND	ND	ND	ND	ND	ND	ND	ND	ND	ND	ND	ND	ND
Campylobacter negative	D3	21d	37	CCFA	6/8/16	Cneg	N.A	ND	ND	ND	ND	ND	ND	ND	ND	ND	ND	ND	ND	ND	ND	ND	ND	ND	ND	ND	ND	ND
D3-0h-42-A	D3	0h	42	CCFA	5/18/16	*Cj*	TK	**8221; 61**	20	20	A	>128	>128	128	128	A	16	16	>32	>32	8	8	1	1	1	1	>256	>256
D3-0h-42-B	D3	0h	42	CCFA	5/18/16	*Cj*	TK	ND	ND	ND	ND	ND	ND	ND	ND	ND	ND	ND	ND	ND	ND	ND	ND	ND	ND	ND	ND	ND
D3-0h-42-C	D3	0h	42	CCFA	5/18/16	*Cj*	TK	ND	ND	ND	ND	ND	ND	ND	ND	ND	ND	ND	ND	ND	ND	ND	ND	ND	ND	ND	ND	ND
D3-0h-42-D	D3	0h	42	CCFA	5/18/16	*Cj*	TK	ND	ND	ND	ND	ND	ND	ND	ND	ND	ND	ND	ND	ND	ND	ND	ND	ND	ND	ND	ND	ND
D3-0h-42-E	D3	0h	42	CCFA	5/18/16	*Cj*	TK	ND	ND	ND	ND	ND	ND	ND	ND	ND	ND	ND	ND	ND	ND	ND	ND	ND	ND	ND	ND	ND
D3-0h-42-F	D3	0h	42	CCFA	5/18/16	*Cj*	TK	ND	ND	ND	ND	ND	ND	ND	ND	ND	ND	ND	ND	ND	ND	ND	ND	ND	ND	ND	ND	ND
D3-12h-42-A	D3	12h	42	CCFA	5/19/16	*Cj*	TK	ND	20	20	ND	>128	>128	ND	ND	ND	16	16	ND	ND	8	8	1	1	1	1	>256	>256
D3-12h-42-B	D3	12h	42	CCFA	5/19/16	*Cj*	TK	ND	ND	ND	ND	ND	ND	ND	ND	ND	ND	ND	ND	ND	ND	ND	ND	ND	ND	ND	ND	ND

Table S1 continued

									CEF MIC		TET					AMP					NAL MIC		CIP MIC		ENR MIC		KAN MIC	
									Trial			MIC					MIC				Trial		Trial		Trial		Trial	
												Trial					Trial											
Isolate	Steer	Sampling time point	Incubation temp. (°C)	Treatment	Sampling date	Species	AMR	ST; CC	1	2	Level	1	2	3	4	Level	1	2	3	4	1	2	1	2	1	2	1	2
D3-12h-42-C	D3	12h	42	CCFA	5/19/16	*Cj*	TK	ND	ND	ND	ND	ND	ND	ND	ND	ND	ND	ND	ND	ND	ND	ND	ND	ND	ND	ND	ND	ND
D3-12h-42-D	D3	12h	42	CCFA	5/19/16	*Cj*	TK	ND	ND	ND	ND	ND	ND	ND	ND	ND	ND	ND	ND	ND	ND	ND	ND	ND	ND	ND	ND	ND
D3-12h-42-E	D3	12h	42	CCFA	5/19/16	*Cj*	TK	ND	ND	ND	ND	ND	ND	ND	ND	ND	ND	ND	ND	ND	ND	ND	ND	ND	ND	ND	ND	ND
D3-12h-42-F	D3	12h	42	CCFA	5/19/16	*Cj*	TK	ND	ND	ND	ND	ND	ND	ND	ND	ND	ND	ND	ND	ND	ND	ND	ND	ND	ND	ND	ND	ND
D3-24h-42-A	D3	24h	42	CCFA	5/19/16	*Cj*	TK	ND	20	20	ND	>128	>128	ND	ND	ND	16	16	ND	ND	8	8	1	1	1	1	>256	>256
D3-24h-42-B	D3	24h	42	CCFA	5/19/16	*Cj*	TK	ND	ND	ND	ND	ND	ND	ND	ND	ND	ND	ND	ND	ND	ND	ND	ND	ND	ND	ND	ND	ND
D3-24h-42-C	D3	24h	42	CCFA	5/19/16	*Cj*	TK	ND	ND	ND	ND	ND	ND	ND	ND	ND	ND	ND	ND	ND	ND	ND	ND	ND	ND	ND	ND	ND
D3-24h-42-D	D3	24h	42	CCFA	5/19/16	*Cj*	TK	ND	ND	ND	ND	ND	ND	ND	ND	ND	ND	ND	ND	ND	ND	ND	ND	ND	ND	ND	ND	ND
D3-24h-42-E	D3	24h	42	CCFA	5/19/16	*Cj*	TK	ND	ND	ND	ND	ND	ND	ND	ND	ND	ND	ND	ND	ND	ND	ND	ND	ND	ND	ND	ND	ND
D3-24h-42-F	D3	24h	42	CCFA	5/19/16	*Cj*	TK	ND	ND	ND	ND	ND	ND	ND	ND	ND	ND	ND	ND	ND	ND	ND	ND	ND	ND	ND	ND	ND
D3-32h-42-A	D3	32h	42	CCFA	5/20/16	*Cj*	TK	ND	ND	ND	ND	ND	ND	ND	ND	ND	ND	ND	ND	ND	ND	ND	ND	ND	ND	ND	ND	ND
D3-32h-42-B	D3	32h	42	CCFA	5/20/16	*Cj*	TK	ND	ND	ND	ND	ND	ND	ND	ND	ND	ND	ND	ND	ND	ND	ND	ND	ND	ND	ND	ND	ND
D3-32h-42-C	D3	32h	42	CCFA	5/20/16	*Cj*	TK	ND	ND	ND	ND	ND	ND	ND	ND	ND	ND	ND	ND	ND	ND	ND	ND	ND	ND	ND	ND	ND
D3-32h-42-D	D3	32h	42	CCFA	5/20/16	*Cj*	TK	ND	ND	ND	ND	ND	ND	ND	ND	ND	ND	ND	ND	ND	ND	ND	ND	ND	ND	ND	ND	ND
D3-48h-42-A	D3	48h	42	CCFA	5/20/16	*Cj*	TK	ND	ND	ND	ND	ND	ND	ND	ND	ND	ND	ND	ND	ND	ND	ND	ND	ND	ND	ND	ND	ND
D3-48h-42-B	D3	48h	42	CCFA	5/20/16	*Cj*	TK	ND	ND	ND	ND	ND	ND	ND	ND	ND	ND	ND	ND	ND	ND	ND	ND	ND	ND	ND	ND	ND
D3-48h-42-C	D3	48h	42	CCFA	5/20/16	*Cj*	TK	ND	ND	ND	ND	ND	ND	ND	ND	ND	ND	ND	ND	ND	ND	ND	ND	ND	ND	ND	ND	ND
D3-48h-42-D	D3	48h	42	CCFA	5/20/16	*Cj*	TK	ND	ND	ND	ND	ND	ND	ND	ND	ND	ND	ND	ND	ND	ND	ND	ND	ND	ND	ND	ND	ND
D3-72h-42-A	D3	72h	42	CCFA	5/21/16	*Cj*	TK	**8221; 61**	20	20	B	128	128	128	128	A	16	16	>32	>32	8	8	1	1	1	1	>256	>256
D3-72h-42-B	D3	72h	42	CCFA	5/21/16	*Cj*	TK	ND	ND	ND	ND	ND	ND	ND	ND	ND	ND	ND	ND	ND	ND	ND	ND	ND	ND	ND	ND	ND
D3-72h-42-C	D3	72h	42	CCFA	5/21/16	*Cj*	TK	ND	ND	ND	ND	ND	ND	ND	ND	ND	ND	ND	ND	ND	ND	ND	ND	ND	ND	ND	ND	ND
D3-72h-42-D	D3	72h	42	CCFA	5/21/16	*Cj*	TK	ND	ND	ND	ND	ND	ND	ND	ND	ND	ND	ND	ND	ND	ND	ND	ND	ND	ND	ND	ND	ND
D3-96h-42-A	D3	96h	42	CCFA	5/22/16	*Cj*	TK	ND	ND	ND	ND	ND	ND	ND	ND	ND	ND	ND	ND	ND	ND	ND	ND	ND	ND	ND	ND	ND
D3-96h-42-B	D3	96h	42	CCFA	5/22/16	*Cj*	TK	ND	ND	ND	ND	ND	ND	ND	ND	ND	ND	ND	ND	ND	ND	ND	ND	ND	ND	ND	ND	ND
D3-96h-42-D	D3	96h	42	CCFA	5/22/16	*Cj*	TK	ND	ND	ND	ND	ND	ND	ND	ND	ND	ND	ND	ND	ND	ND	ND	ND	ND	ND	ND	ND	ND
D3-5d-42-A	D3	5d	42	CCFA	5/23/16	*Cj*	TK	21; 21	20	20	A	>128	>128	128	128	B	16	16	32	32	8	8	1	1	1	1	>256	>256
D3-5d-42-B	D3	5d	42	CCFA	5/23/16	*Cj*	TK	ND	ND	ND	ND	ND	ND	ND	ND	ND	ND	ND	ND	ND	ND	ND	ND	ND	ND	ND	ND	ND
D3-5d-42-C	D3	5d	42	CCFA	5/23/16	*Cj*	TK	ND	ND	ND	ND	ND	ND	ND	ND	ND	ND	ND	ND	ND	ND	ND	ND	ND	ND	ND	ND	ND
D3-6d-42-A	D3	6d	42	CCFA	5/24/16	*Cj*	PS	21; 21	ND	ND	ND	ND	ND	ND	ND	ND	ND	ND	ND	ND	ND	ND	ND	ND	ND	ND	ND	ND
D3-6d-42-B	D3	6d	42	CCFA	5/24/16	*Cj*	PS	ND	ND	ND	ND	ND	ND	ND	ND	ND	ND	ND	ND	ND	ND	ND	ND	ND	ND	ND	ND	ND
D3-6d-42-C	D3	6d	42	CCFA	5/24/16	*Cj*	PS	ND	ND	ND	ND	ND	ND	ND	ND	ND	ND	ND	ND	ND	ND	ND	ND	ND	ND	ND	ND	ND
D3-6d-42-D	D3	6d	42	CCFA	5/24/16	*Cj*	PS	ND	ND	ND	ND	ND	ND	ND	ND	ND	ND	ND	ND	ND	ND	ND	ND	ND	ND	ND	ND	ND
D3-7d-42-A	D3	7d	42	CCFA	5/25/16	*Cj*	PS	21; 21	ND	ND	ND	ND	ND	ND	ND	ND	ND	ND	ND	ND	ND	ND	ND	ND	ND	ND	ND	ND
D3-7d-42-B	D3	7d	42	CCFA	5/25/16	*Cj*	TK	ND	ND	ND	ND	ND	ND	ND	ND	ND	ND	ND	ND	ND	ND	ND	ND	ND	ND	ND	ND	ND
D3-7d-42-C	D3	7d	42	CCFA	5/25/16	*Cj*	TK	ND	ND	ND	ND	ND	ND	ND	ND	ND	ND	ND	ND	ND	ND	ND	ND	ND	ND	ND	ND	ND
D3-7d-42-D	D3	7d	42	CCFA	5/25/16	*Cj*	TK	ND	ND	ND	ND	ND	ND	ND	ND	ND	ND	ND	ND	ND	ND	ND	ND	ND	ND	ND	ND	ND
D3-8d-42-A	D3	8d	42	CCFA	5/26/16	*Ch*†	TN	ND	ND	ND	ND	ND	ND	ND	ND	ND	ND	ND	ND	ND	ND	ND	ND	ND	ND	ND	ND	ND
D3-8d-42-B	D3	8d	42	CCFA	5/26/16	*Ch*†	TN	6 (Chy)	ND	ND	ND	ND	ND	ND	ND	ND	ND	ND	ND	ND	ND	ND	ND	ND	ND	ND	ND	ND
D3-8d-42-D	D3	8d	42	CCFA	5/26/16	*Ch*†	TN	ND	ND	ND	ND	ND	ND	ND	ND	ND	ND	ND	ND	ND	ND	ND	ND	ND	ND	ND	ND	ND
D3-14d-42-A	D3	14d	42	CCFA	6/1/16	*Cj*	TK	**8221; 61**	20	20	A	>128	>128	128	128	A	16	16	>32	>32	8	8	1	1	1	1	>256	>256
D3-14d-42-B	D3	14d	42	CCFA	6/1/16	*Cj*	TK	ND	ND	ND	ND	ND	ND	ND	ND	ND	ND	ND	ND	ND	ND	ND	ND	ND	ND	ND	ND	ND
D3-14d-42-C	D3	14d	42	CCFA	6/1/16	*Cj*	TK	ND	ND	ND	ND	ND	ND	ND	ND	ND	ND	ND	ND	ND	ND	ND	ND	ND	ND	ND	ND	ND
D4-0h-37-A	D4	0h	37	CCFA	5/18/16	*Cf*	N	**8221; 61**	ND	ND	ND	ND	ND	ND	ND	ND	ND	ND	ND	ND	ND	ND	ND	ND	ND	ND	ND	ND
D4-0h-37-B	D4	0h	37	CCFA	5/18/16	*Cf*	N	ND	ND	ND	ND	ND	ND	ND	ND	ND	ND	ND	ND	ND	ND	ND	ND	ND	ND	ND	ND	ND

Table S1 continued

											TET					AMP												
									CEF MIC			MIC					MIC				NAL MIC		CIP MIC		ENR MIC		KAN MIC	
									Trial			Trial					Trial				Trial		Trial		Trial		Trial	
Isolate	Steer	Sampling time point	Incubation temp. (°C)	Treatment	Sampling date	Species	AMR	ST; CC	1	2	Level	1	2	3	4	Level	1	2	3	4	1	2	1	2	1	2	1	2
D4-0h-37-C	D4	0h	37	CCFA	5/18/16	*Cf*	N	ND	ND	ND	ND	ND	ND	ND	ND	ND	ND	ND	ND	ND	ND	ND	ND	ND	ND	ND	ND	ND
D4-0h-37-D	D4	0h	37	CCFA	5/18/16	*Cf*	N	ND	ND	ND	ND	ND	ND	ND	ND	ND	ND	ND	ND	ND	ND	ND	ND	ND	ND	ND	ND	ND
D4-0h-37-E	D4	0h	37	CCFA	5/18/16	*Cf*	N	ND	ND	ND	ND	ND	ND	ND	ND	ND	ND	ND	ND	ND	ND	ND	ND	ND	ND	ND	ND	ND
D4-0h-37-F	D4	0h	37	CCFA	5/18/16	*Cf*	N	ND	ND	ND	ND	ND	ND	ND	ND	ND	ND	ND	ND	ND	ND	ND	ND	ND	ND	ND	ND	ND
D4-12h-37-A	D4	12h	37	CCFA	5/19/16	*Cf*	N	6 (Cfe)	ND	ND	ND	ND	ND	ND	ND	ND	ND	ND	ND	ND	ND	ND	ND	ND	ND	ND	ND	ND
D4-12h-37-B	D4	12h	37	CCFA	5/19/16	*Cf*	N	ND	ND	ND	ND	ND	ND	ND	ND	ND	ND	ND	ND	ND	ND	ND	ND	ND	ND	ND	ND	ND
D4-12h-37-C	D4	12h	37	CCFA	5/19/16	*Cf*	N	ND	ND	ND	ND	ND	ND	ND	ND	ND	ND	ND	ND	ND	ND	ND	ND	ND	ND	ND	ND	ND
D4-12h-37-D	D4	12h	37	CCFA	5/19/16	*Cf*	N	ND	ND	ND	ND	ND	ND	ND	ND	ND	ND	ND	ND	ND	ND	ND	ND	ND	ND	ND	ND	ND
D4-12h-37-E	D4	12h	37	CCFA	5/19/16	*Cf*	N	ND	ND	ND	ND	ND	ND	ND	ND	ND	ND	ND	ND	ND	ND	ND	ND	ND	ND	ND	ND	ND
D4-12h-37-F	D4	12h	37	CCFA	5/19/16	*Cf*	N	ND	ND	ND	ND	ND	ND	ND	ND	ND	ND	ND	ND	ND	ND	ND	ND	ND	ND	ND	ND	ND
D4-24h-37-A	D4	24h	37	CCFA	5/19/16	*Cj*	PS	ND	ND	ND	ND	ND	ND	ND	ND	ND	ND	ND	ND	ND	ND	ND	ND	ND	ND	ND	ND	ND
D4-24h-37-B	D4	24h	37	CCFA	5/19/16	*Cj*	PS	21; 21	ND	ND	ND	ND	ND	ND	ND	ND	ND	ND	ND	ND	ND	ND	ND	ND	ND	ND	ND	ND
D4-24h-37-C	D4	24h	37	CCFA	5/19/16	*Cj*	TK	**8567; 21**	ND	ND	ND	ND	ND	ND	ND	ND	ND	ND	ND	ND	ND	ND	ND	ND	ND	ND	ND	ND
D4-24h-37-E	D4	24h	37	CCFA	5/19/16	*Cj*	PS	ND	ND	ND	ND	ND	ND	ND	ND	ND	ND	ND	ND	ND	ND	ND	ND	ND	ND	ND	ND	ND
D4-32h-37-A	D4	32h	37	CCFA	5/20/16	*Cj*	PS	ND	ND	ND	ND	ND	ND	ND	ND	ND	ND	ND	ND	ND	ND	ND	ND	ND	ND	ND	ND	ND
D4-32h-37-B	D4	32h	37	CCFA	5/20/16	*Cj*	TK	ND	ND	ND	ND	ND	ND	ND	ND	ND	ND	ND	ND	ND	ND	ND	ND	ND	ND	ND	ND	ND
D4-32h-37-C	D4	32h	37	CCFA	5/20/16	*Cj*	TK	ND	ND	ND	ND	ND	ND	ND	ND	ND	ND	ND	ND	ND	ND	ND	ND	ND	ND	ND	ND	ND
D4-32h-37-D	D4	32h	37	CCFA	5/20/16	*Cj*	TK	ND	ND	ND	ND	ND	ND	ND	ND	ND	ND	ND	ND	ND	ND	ND	ND	ND	ND	ND	ND	ND
D4-48h-37-A	D4	48h	37	CCFA	5/20/16	*Cj*	TK	ND	ND	ND	ND	ND	ND	ND	ND	ND	ND	ND	ND	ND	ND	ND	ND	ND	ND	ND	ND	ND
D4-48h-37-B	D4	48h	37	CCFA	5/20/16	*Cj*	TK	ND	ND	ND	ND	ND	ND	ND	ND	ND	ND	ND	ND	ND	ND	ND	ND	ND	ND	ND	ND	ND
D4-48h-37-C	D4	48h	37	CCFA	5/20/16	*Cj*	TK	ND	ND	ND	ND	ND	ND	ND	ND	ND	ND	ND	ND	ND	ND	ND	ND	ND	ND	ND	ND	ND
D4-48h-37-D	D4	48h	37	CCFA	5/20/16	*Cj*	TK	ND	ND	ND	ND	ND	ND	ND	ND	ND	ND	ND	ND	ND	ND	ND	ND	ND	ND	ND	ND	ND
D4-72h-37-A	D4	72h	37	CCFA	5/21/16	*Cj*	TK	ND	ND	ND	ND	ND	ND	ND	ND	ND	ND	ND	ND	ND	ND	ND	ND	ND	ND	ND	ND	ND
D4-72h-37-B	D4	72h	37	CCFA	5/21/16	*Cj*	TK	ND	ND	ND	ND	ND	ND	ND	ND	ND	ND	ND	ND	ND	ND	ND	ND	ND	ND	ND	ND	ND
D4-72h-37-C	D4	72h	37	CCFA	5/21/16	*Cj*	TK	ND	ND	ND	ND	ND	ND	ND	ND	ND	ND	ND	ND	ND	ND	ND	ND	ND	ND	ND	ND	ND
D4-72h-37-D	D4	72h	37	CCFA	5/21/16	*Cj*	TK	ND	ND	ND	ND	ND	ND	ND	ND	ND	ND	ND	ND	ND	ND	ND	ND	ND	ND	ND	ND	ND
D4-96h-37-A	D4	96h	37	CCFA	5/22/16	*Cj*	TK	**8567; 21**	ND	ND	ND	ND	ND	ND	ND	ND	ND	ND	ND	ND	ND	ND	ND	ND	ND	ND	ND	ND
D4-96h-37-B	D4	96h	37	CCFA	5/22/16	*Cj*	PS	ND	ND	ND	ND	ND	ND	ND	ND	ND	ND	ND	ND	ND	ND	ND	ND	ND	ND	ND	ND	ND
D4-96h-37-C	D4	96h	37	CCFA	5/22/16	*Cj*	TK	ND	ND	ND	ND	ND	ND	ND	ND	ND	ND	ND	ND	ND	ND	ND	ND	ND	ND	ND	ND	ND
D4-96h-37-D	D4	96h	37	CCFA	5/22/16	*Cj*	TK	ND	ND	ND	ND	ND	ND	ND	ND	ND	ND	ND	ND	ND	ND	ND	ND	ND	ND	ND	ND	ND
D4-5d-37-A	D4	5d	37	CCFA	5/23/16	*Cj*	PS	ND	ND	ND	ND	ND	ND	ND	ND	ND	ND	ND	ND	ND	ND	ND	ND	ND	ND	ND	ND	ND
D4-5d-37-B	D4	5d	37	CCFA	5/23/16	*Cj*	PS	ND	ND	ND	ND	ND	ND	ND	ND	ND	ND	ND	ND	ND	ND	ND	ND	ND	ND	ND	ND	ND
D4-5d-37-C	D4	5d	37	CCFA	5/23/16	*Cj*	PS	ND	ND	ND	ND	ND	ND	ND	ND	ND	ND	ND	ND	ND	ND	ND	ND	ND	ND	ND	ND	ND
D4-5d-37-D	D4	5d	37	CCFA	5/23/16	*Cj*	PS	ND	ND	ND	ND	ND	ND	ND	ND	ND	ND	ND	ND	ND	ND	ND	ND	ND	ND	ND	ND	ND
D4-6d-37-A	D4	6d	37	CCFA	5/24/16	*Cj*	PS	ND	ND	ND	ND	ND	ND	ND	ND	ND	ND	ND	ND	ND	ND	ND	ND	ND	ND	ND	ND	ND
D4-6d-37-B	D4	6d	37	CCFA	5/24/16	*Cj*	PS	ND	ND	ND	ND	ND	ND	ND	ND	ND	ND	ND	ND	ND	ND	ND	ND	ND	ND	ND	ND	ND
D4-6d-37-C	D4	6d	37	CCFA	5/24/16	*Cj*	PS	ND	ND	ND	ND	ND	ND	ND	ND	ND	ND	ND	ND	ND	ND	ND	ND	ND	ND	ND	ND	ND
D4-6d-37-D	D4	6d	37	CCFA	5/24/16	*Cj*	PS	ND	ND	ND	ND	ND	ND	ND	ND	ND	ND	ND	ND	ND	ND	ND	ND	ND	ND	ND	ND	ND
D4-7d-37-A	D4	7d	37	CCFA	5/25/16	*Cj*	PS	21; 21	ND	ND	ND	ND	ND	ND	ND	ND	ND	ND	ND	ND	ND	ND	ND	ND	ND	ND	ND	ND
D4-7d-37-B	D4	7d	37	CCFA	5/25/16	*Cj*	PS	ND	ND	ND	ND	ND	ND	ND	ND	ND	ND	ND	ND	ND	ND	ND	ND	ND	ND	ND	ND	ND
D4-7d-37-C	D4	7d	37	CCFA	5/25/16	*Cj*	PS	ND	ND	ND	ND	ND	ND	ND	ND	ND	ND	ND	ND	ND	ND	ND	ND	ND	ND	ND	ND	ND
Campylobacter negative	D4	8d	37	CCFA	5/26/16	Cneg	N.A	ND	ND	ND	ND	ND	ND	ND	ND	ND	ND	ND	ND	ND	ND	ND	ND	ND	ND	ND	ND	ND
D4-21d-37-A	D4	21d	37	CCFA	6/8/16	*Cj*	PS	ND	ND	ND	ND	ND	ND	ND	ND	ND	ND	ND	ND	ND	ND	ND	ND	ND	ND	ND	ND	ND
Campylobacter negative	D4	0h	42	CCFA	5/18/16	Cneg	N.A	ND	ND	ND	ND	ND	ND	ND	ND	ND	ND	ND	ND	ND	ND	ND	ND	ND	ND	ND	ND	ND

Table S1 continued

									CEF MIC		TET					AMP					NAL MIC		CIP MIC		ENR MIC		KAN MIC	
									Trial			MIC Trial					MIC Trial				Trial		Trial		Trial		Trial	
Isolate	Steer	Sampling time point	Incubation temp. (°C)	Treatment	Sampling date	Species	AMR	ST; CC	1	2	Level	1	2	3	4	Level	1	2	3	4	1	2	1	2	1	2	1	2
D4-12h-42-A	D4	12h	42	CCFA	5/19/16	*Cj*	PS	21; 21	ND	ND	ND	ND	ND	ND	ND	ND	ND	ND	ND	ND	ND	ND	ND	ND	ND	ND	ND	ND
D4-12h-42-B	D4	12h	42	CCFA	5/19/16	*Cj*	PS	ND	ND	ND	ND	ND	ND	ND	ND	ND	ND	ND	ND	ND	ND	ND	ND	ND	ND	ND	ND	ND
D4-12h-42-C	D4	12h	42	CCFA	5/19/16	*Cj*	PS	ND	ND	ND	ND	ND	ND	ND	ND	ND	ND	ND	ND	ND	ND	ND	ND	ND	ND	ND	ND	ND
D4-12h-42-D	D4	12h	42	CCFA	5/19/16	*Cj*	PS	ND	ND	ND	ND	ND	ND	ND	ND	ND	ND	ND	ND	ND	ND	ND	ND	ND	ND	ND	ND	ND
D4-12h-42-F	D4	12h	42	CCFA	5/19/16	*Cj*	PS	ND	ND	ND	ND	ND	ND	ND	ND	ND	ND	ND	ND	ND	ND	ND	ND	ND	ND	ND	ND	ND
D4-24h-42-A	D4	24h	42	CCFA	5/19/16	*Cj*	PS	ND	ND	ND	ND	ND	ND	ND	ND	ND	ND	ND	ND	ND	ND	ND	ND	ND	ND	ND	ND	ND
D4-24h-42-B	D4	24h	42	CCFA	5/19/16	*Cj*	PS	ND	ND	ND	ND	ND	ND	ND	ND	ND	ND	ND	ND	ND	ND	ND	ND	ND	ND	ND	ND	ND
D4-24h-42-C	D4	24h	42	CCFA	5/19/16	*Cj*	TK	ND	ND	ND	ND	ND	ND	ND	ND	ND	ND	ND	ND	ND	ND	ND	ND	ND	ND	ND	ND	ND
D4-24h-42-D	D4	24h	42	CCFA	5/19/16	*Cj*	PS	ND	ND	ND	ND	ND	ND	ND	ND	ND	ND	ND	ND	ND	ND	ND	ND	ND	ND	ND	ND	ND
D4-24h-42-E	D4	24h	42	CCFA	5/19/16	*Cj*	PS	ND	ND	ND	ND	ND	ND	ND	ND	ND	ND	ND	ND	ND	ND	ND	ND	ND	ND	ND	ND	ND
D4-24h-42-F	D4	24h	42	CCFA	5/19/16	*Cj*	PS	ND	ND	ND	ND	ND	ND	ND	ND	ND	ND	ND	ND	ND	ND	ND	ND	ND	ND	ND	ND	ND
D4-32h-42-A	D4	32h	42	CCFA	5/20/16	*Cj*	PS	ND	ND	ND	ND	ND	ND	ND	ND	ND	ND	ND	ND	ND	ND	ND	ND	ND	ND	ND	ND	ND
D4-32h-42-B	D4	32h	42	CCFA	5/20/16	*Cj*	PS	ND	ND	ND	ND	ND	ND	ND	ND	ND	ND	ND	ND	ND	ND	ND	ND	ND	ND	ND	ND	ND
D4-32h-42-D	D4	32h	42	CCFA	5/20/16	*Cj*	TK	21; 21	ND	ND	ND	ND	ND	ND	ND	ND	ND	ND	ND	ND	ND	ND	ND	ND	ND	ND	ND	ND
D4-48h-42-A	D4	48h	42	CCFA	5/20/16	*Cj*	TK	ND	ND	ND	ND	ND	ND	ND	ND	ND	ND	ND	ND	ND	ND	ND	ND	ND	ND	ND	ND	ND
D4-48h-42-B	D4	48h	42	CCFA	5/20/16	*Cj*	TK	ND	ND	ND	ND	ND	ND	ND	ND	ND	ND	ND	ND	ND	ND	ND	ND	ND	ND	ND	ND	ND
D4-48h-42-C	D4	48h	42	CCFA	5/20/16	*Cj*	TK	ND	ND	ND	ND	ND	ND	ND	ND	ND	ND	ND	ND	ND	ND	ND	ND	ND	ND	ND	ND	ND
D4-48h-42-D	D4	48h	42	CCFA	5/20/16	*Cj*	TK	ND	ND	ND	ND	ND	ND	ND	ND	ND	ND	ND	ND	ND	ND	ND	ND	ND	ND	ND	ND	ND
D4-72h-42-A	D4	72h	42	CCFA	5/21/16	*Cj*	TK	ND	ND	ND	ND	ND	ND	ND	ND	ND	ND	ND	ND	ND	ND	ND	ND	ND	ND	ND	ND	ND
D4-72h-42-B	D4	72h	42	CCFA	5/21/16	*Cj*	TK	ND	ND	ND	ND	ND	ND	ND	ND	ND	ND	ND	ND	ND	ND	ND	ND	ND	ND	ND	ND	ND
D4-72h-42-C	D4	72h	42	CCFA	5/21/16	*Cj*	TK	ND	ND	ND	ND	ND	ND	ND	ND	ND	ND	ND	ND	ND	ND	ND	ND	ND	ND	ND	ND	ND
D4-72h-42-D	D4	72h	42	CCFA	5/21/16	*Cj*	TK	ND	ND	ND	ND	ND	ND	ND	ND	ND	ND	ND	ND	ND	ND	ND	ND	ND	ND	ND	ND	ND
D4-96h-42-A	D4	96h	42	CCFA	5/22/16	*Cj*	TK	ND	ND	ND	ND	ND	ND	ND	ND	ND	ND	ND	ND	ND	ND	ND	ND	ND	ND	ND	ND	ND
D4-96h-42-B	D4	96h	42	CCFA	5/22/16	*Cj*	TK	ND	ND	ND	ND	ND	ND	ND	ND	ND	ND	ND	ND	ND	ND	ND	ND	ND	ND	ND	ND	ND
D4-96h-42-C	D4	96h	42	CCFA	5/22/16	*Cj*	TK	ND	ND	ND	ND	ND	ND	ND	ND	ND	ND	ND	ND	ND	ND	ND	ND	ND	ND	ND	ND	ND
D4-96h-42-D	D4	96h	42	CCFA	5/22/16	*Cj*	TK	ND	ND	ND	ND	ND	ND	ND	ND	ND	ND	ND	ND	ND	ND	ND	ND	ND	ND	ND	ND	ND
D4-5d-42-A	D4	5d	42	CCFA	5/23/16	*Cj*	TK	ND	ND	ND	ND	ND	ND	ND	ND	ND	ND	ND	ND	ND	ND	ND	ND	ND	ND	ND	ND	ND
D4-5d-42-B	D4	5d	42	CCFA	5/23/16	*Cj*	TK	ND	ND	ND	ND	ND	ND	ND	ND	ND	ND	ND	ND	ND	ND	ND	ND	ND	ND	ND	ND	ND
D4-5d-42-C	D4	5d	42	CCFA	5/23/16	*Cj*	TK	ND	ND	ND	ND	ND	ND	ND	ND	ND	ND	ND	ND	ND	ND	ND	ND	ND	ND	ND	ND	ND
D4-6d-42-A	D4	6d	42	CCFA	5/24/16	*Cj*	PS	ND	ND	ND	ND	ND	ND	ND	ND	ND	ND	ND	ND	ND	ND	ND	ND	ND	ND	ND	ND	ND
D4-6d-42-B	D4	6d	42	CCFA	5/24/16	*Cj*	PS	ND	ND	ND	ND	ND	ND	ND	ND	ND	ND	ND	ND	ND	ND	ND	ND	ND	ND	ND	ND	ND
D4-6d-42-C	D4	6d	42	CCFA	5/24/16	*Cj*	PS	ND	ND	ND	ND	ND	ND	ND	ND	ND	ND	ND	ND	ND	ND	ND	ND	ND	ND	ND	ND	ND
D4-6d-42-D	D4	6d	42	CCFA	5/24/16	*Cj*	PS	ND	ND	ND	ND	ND	ND	ND	ND	ND	ND	ND	ND	ND	ND	ND	ND	ND	ND	ND	ND	ND
D4-7d-42-A	D4	7d	42	CCFA	5/25/16	*Cj*	TK	376; 21	ND	ND	ND	ND	ND	ND	ND	ND	ND	ND	ND	ND	ND	ND	ND	ND	ND	ND	ND	ND
D4-7d-42-B	D4	7d	42	CCFA	5/25/16	*Cj*	PS	**8573; 21**	ND	ND	ND	ND	ND	ND	ND	ND	ND	ND	ND	ND	ND	ND	ND	ND	ND	ND	ND	ND
D4-7d-42-C	D4	7d	42	CCFA	5/25/16	*Cj*	PS	ND	ND	ND	ND	ND	ND	ND	ND	ND	ND	ND	ND	ND	ND	ND	ND	ND	ND	ND	ND	ND
D4-7d-42-D	D4	7d	42	CCFA	5/25/16	*Cj*	TK	ND	ND	ND	ND	ND	ND	ND	ND	ND	ND	ND	ND	ND	ND	ND	ND	ND	ND	ND	ND	ND
Campylobacter negative	D4	8d	42	CCFA	5/26/16	Cneg	N.A	ND	ND	ND	ND	ND	ND	ND	ND	ND	ND	ND	ND	ND	ND	ND	ND	ND	ND	ND	ND	ND
Campylobacter negative	D4	14d	42	CCFA	6/1/16	Cneg	N.A	ND	ND	ND	ND	ND	ND	ND	ND	ND	ND	ND	ND	ND	ND	ND	ND	ND	ND	ND	ND	ND
Campylobacter negative	D5	0h	42	CCFA	6/1/16	Cneg	N.A	ND	ND	ND	ND	ND	ND	ND	ND	ND	ND	ND	ND	ND	ND	ND	ND	ND	ND	ND	ND	ND
Campylobacter negative	D5	12h	42	CCFA	6/2/16	Cneg	N.A	ND	ND	ND	ND	ND	ND	ND	ND	ND	ND	ND	ND	ND	ND	ND	ND	ND	ND	ND	ND	ND
D5-24h-42-A	D5	24h	42	CCFA	6/2/16	*Cj*	PS	21; 21	ND	ND	ND	ND	ND	ND	ND	ND	ND	ND	ND	ND	ND	ND	ND	ND	ND	ND	ND	ND
D5-24h-42-B	D5	24h	42	CCFA	6/2/16	*Cj*	PS	ND	ND	ND	ND	ND	ND	ND	ND	ND	ND	ND	ND	ND	ND	ND	ND	ND	ND	ND	ND	ND
D5-32h-42-A	D5	32h	42	CCFA	6/3/16	*Cj*	PS	ND	ND	ND	ND	ND	ND	ND	ND	ND	ND	ND	ND	ND	ND	ND	ND	ND	ND	ND	ND	ND

Table S1 continued

									CEF MIC		TET					AMP					NAL MIC		CIP MIC		ENR MIC		KAN MIC	
									Trial			MIC					MIC				Trial		Trial		Trial		Trial	
												Trial					Trial											
Isolate	Steer	Sampling time point	Incubation temp. (°C)	Treatment	Sampling date	Species	AMR	ST; CC	1	2	Level	1	2	3	4	Level	1	2	3	4	1	2	1	2	1	2	1	2
D5-32h-42-B	D5	32h	42	CCFA	6/3/16	*Cj*	PS	ND	ND	ND	ND	ND	ND	ND	ND	ND	ND	ND	ND	ND	ND	ND	ND	ND	ND	ND	ND	ND
D5-32h-42-C	D5	32h	42	CCFA	6/3/16	*Cj*	PS	ND	ND	ND	ND	ND	ND	ND	ND	ND	ND	ND	ND	ND	ND	ND	ND	ND	ND	ND	ND	ND
D5-32h-42-D	D5	32h	42	CCFA	6/3/16	*Cj*	PS	ND	ND	ND	ND	ND	ND	ND	ND	ND	ND	ND	ND	ND	ND	ND	ND	ND	ND	ND	ND	ND
D5-48h-42-A	D5	48h	42	CCFA	6/3/16	*Cj*	TK	**8221; 61**	ND	ND	ND	ND	ND	ND	ND	ND	ND	ND	ND	ND	ND	ND	ND	ND	ND	ND	ND	ND
D5-48h-42-B	D5	48h	42	CCFA	6/3/16	*Cj*	PS	21; 21	ND	ND	ND	ND	ND	ND	ND	ND	ND	ND	ND	ND	ND	ND	ND	ND	ND	ND	ND	ND
D5-48h-42-C	D5	48h	42	CCFA	6/3/16	*Cj*	PS	ND	ND	ND	ND	ND	ND	ND	ND	ND	ND	ND	ND	ND	ND	ND	ND	ND	ND	ND	ND	ND
D5-48h-42-D	D5	48h	42	CCFA	6/3/16	*Cj*	TK	ND	ND	ND	ND	ND	ND	ND	ND	ND	ND	ND	ND	ND	ND	ND	ND	ND	ND	ND	ND	ND
D5-72h-37-A	D5	72h	37	CCFA	6/4/16	*Cj*	PS	ND	ND	ND	ND	ND	ND	ND	ND	ND	ND	ND	ND	ND	ND	ND	ND	ND	ND	ND	ND	ND
D5-72h-37-B	D5	72h	37	CCFA	6/4/16	*Cj*	PS	ND	ND	ND	ND	ND	ND	ND	ND	ND	ND	ND	ND	ND	ND	ND	ND	ND	ND	ND	ND	ND
D5-72h-37-C	D5	72h	37	CCFA	6/4/16	*Cj*	PS	ND	ND	ND	ND	ND	ND	ND	ND	ND	ND	ND	ND	ND	ND	ND	ND	ND	ND	ND	ND	ND
D5-72h-37-D	D5	72h	37	CCFA	6/4/16	*Cj*	PS	ND	ND	ND	ND	ND	ND	ND	ND	ND	ND	ND	ND	ND	ND	ND	ND	ND	ND	ND	ND	ND
D5-72h-37-E	D5	72h	37	CCFA	6/4/16	*Cj*	PS	ND	ND	ND	ND	ND	ND	ND	ND	ND	ND	ND	ND	ND	ND	ND	ND	ND	ND	ND	ND	ND
D5-72h-37-F	D5	72h	37	CCFA	6/4/16	*Cj*	PS	ND	ND	ND	ND	ND	ND	ND	ND	ND	ND	ND	ND	ND	ND	ND	ND	ND	ND	ND	ND	ND
D5-96h-37-A	D5	96h	37	CCFA	6/5/16	*Cj*	PS	ND	ND	ND	ND	ND	ND	ND	ND	ND	ND	ND	ND	ND	ND	ND	ND	ND	ND	ND	ND	ND
D5-96h-37-B	D5	96h	37	CCFA	6/5/16	*Cj*	PS	ND	ND	ND	ND	ND	ND	ND	ND	ND	ND	ND	ND	ND	ND	ND	ND	ND	ND	ND	ND	ND
D5-96h-37-C	D5	96h	37	CCFA	6/5/16	*Cj*	PS	ND	ND	ND	ND	ND	ND	ND	ND	ND	ND	ND	ND	ND	ND	ND	ND	ND	ND	ND	ND	ND
D5-96h-37-D	D5	96h	37	CCFA	6/5/16	*Cj*	TK	**8221; 61**	ND	ND	ND	ND	ND	ND	ND	ND	ND	ND	ND	ND	ND	ND	ND	ND	ND	ND	ND	ND
D5-96h-37-E	D5	96h	37	CCFA	6/5/16	*Cj*	PS	ND	ND	ND	ND	ND	ND	ND	ND	ND	ND	ND	ND	ND	ND	ND	ND	ND	ND	ND	ND	ND
D5-96h-37-F	D5	96h	37	CCFA	6/5/16	*Cj*	PS	ND	ND	ND	ND	ND	ND	ND	ND	ND	ND	ND	ND	ND	ND	ND	ND	ND	ND	ND	ND	ND
D5-96h-37-I	D5	96h	37	CCFA	6/5/16	*Cj*	PS	ND	ND	ND	ND	ND	ND	ND	ND	ND	ND	ND	ND	ND	ND	ND	ND	ND	ND	ND	ND	ND
D5-96h-37-J	D5	96h	37	CCFA	6/5/16	*Cj*	PS	ND	ND	ND	ND	ND	ND	ND	ND	ND	ND	ND	ND	ND	ND	ND	ND	ND	ND	ND	ND	ND
D5-5d-37-A	D5	5d	37	CCFA	6/6/16	*Cj*	PS	ND	ND	ND	ND	ND	ND	ND	ND	ND	ND	ND	ND	ND	ND	ND	ND	ND	ND	ND	ND	ND
D5-5d-37-B	D5	5d	37	CCFA	6/6/16	*Cj*	PS	ND	ND	ND	ND	ND	ND	ND	ND	ND	ND	ND	ND	ND	ND	ND	ND	ND	ND	ND	ND	ND
Campylobacter negative	D5	6d	37	CCFA	6/7/16	Cneg	N.A	ND	ND	ND	ND	ND	ND	ND	ND	ND	ND	ND	ND	ND	ND	ND	ND	ND	ND	ND	ND	ND
D5-7d-37-A	D5	7d	37	CCFA	6/8/16	*Cj*	PS	21; 21	ND	ND	ND	ND	ND	ND	ND	ND	ND	ND	ND	ND	ND	ND	ND	ND	ND	ND	ND	ND
D5-8d-37-A	D5	8d	37	CCFA	6/9/16	*Cj*	TK	ND	ND	ND	ND	ND	ND	ND	ND	ND	ND	ND	ND	ND	ND	ND	ND	ND	ND	ND	ND	ND
D5-8d-37-B	D5	8d	37	CCFA	6/9/16	*Cj*	TK	ND	ND	ND	ND	ND	ND	ND	ND	ND	ND	ND	ND	ND	ND	ND	ND	ND	ND	ND	ND	ND
D5-12d-37-A	D5	12d	37	CCFA	6/13/16	*Cj*	TK	**8574; 61**	ND	ND	ND	ND	ND	ND	ND	ND	ND	ND	ND	ND	ND	ND	ND	ND	ND	ND	ND	ND
D5-12d-37-B	D5	12d	37	CCFA	6/13/16	*Cj*	TK	ND	ND	ND	ND	ND	ND	ND	ND	ND	ND	ND	ND	ND	ND	ND	ND	ND	ND	ND	ND	ND
D5-12d-37-C	D5	12d	37	CCFA	6/13/16	*Cj*	TK	ND	ND	ND	ND	ND	ND	ND	ND	ND	ND	ND	ND	ND	ND	ND	ND	ND	ND	ND	ND	ND
D5-12d-37-D	D5	12d	37	CCFA	6/13/16	*Cj*	TK	ND	ND	ND	ND	ND	ND	ND	ND	ND	ND	ND	ND	ND	ND	ND	ND	ND	ND	ND	ND	ND
Campylobacter negative	D5	21d	37	CCFA	6/22/16	Cneg	N.A	ND	ND	ND	ND	ND	ND	ND	ND	ND	ND	ND	ND	ND	ND	ND	ND	ND	ND	ND	ND	ND
Campylobacter negative	D5	28d	37	CCFA	6/29/16	Cneg	N.A	ND	ND	ND	ND	ND	ND	ND	ND	ND	ND	ND	ND	ND	ND	ND	ND	ND	ND	ND	ND	ND
Campylobacter negative	D5	35d	37	CCFA	7/6/16	Cneg	N.A	ND	ND	ND	ND	ND	ND	ND	ND	ND	ND	ND	ND	ND	ND	ND	ND	ND	ND	ND	ND	ND
Campylobacter negative	D5	42d	37	CCFA	7/13/16	Cneg	N.A	ND	ND	ND	ND	ND	ND	ND	ND	ND	ND	ND	ND	ND	ND	ND	ND	ND	ND	ND	ND	ND
Campylobacter negative	D6	0h	42	CCFA	6/1/16	Cneg	N.A	ND	ND	ND	ND	ND	ND	ND	ND	ND	ND	ND	ND	ND	ND	ND	ND	ND	ND	ND	ND	ND
Campylobacter negative	D6	12h	42	CCFA	6/2/16	Cneg	N.A	ND	ND	ND	ND	ND	ND	ND	ND	ND	ND	ND	ND	ND	ND	ND	ND	ND	ND	ND	ND	ND
D6-24h-42-A	D6	24h	42	CCFA	6/2/16	*Cj*	TK	**8221; 61**	ND	ND	ND	ND	ND	ND	ND	ND	ND	ND	ND	ND	ND	ND	ND	ND	ND	ND	ND	ND
D6-24h-42-B	D6	24h	42	CCFA	6/2/16	*Cj*	TK	ND	ND	ND	ND	ND	ND	ND	ND	ND	ND	ND	ND	ND	ND	ND	ND	ND	ND	ND	ND	ND
D6-24h-42-C	D6	24h	42	CCFA	6/2/16	*Cj*	TK	ND	ND	ND	ND	ND	ND	ND	ND	ND	ND	ND	ND	ND	ND	ND	ND	ND	ND	ND	ND	ND
D6-24h-42-D	D6	24h	42	CCFA	6/2/16	*Cj*	TK	ND	ND	ND	ND	ND	ND	ND	ND	ND	ND	ND	ND	ND	ND	ND	ND	ND	ND	ND	ND	ND
D6-32h-42-A	D6	32h	42	CCFA	6/3/16	*Cj*	TK	ND	ND	ND	ND	ND	ND	ND	ND	ND	ND	ND	ND	ND	ND	ND	ND	ND	ND	ND	ND	ND
D6-32h-42-B	D6	32h	42	CCFA	6/3/16	*Cj*	TK	ND	ND	ND	ND	ND	ND	ND	ND	ND	ND	ND	ND	ND	ND	ND	ND	ND	ND	ND	ND	ND
D6-32h-42-C	D6	32h	42	CCFA	6/3/16	*Cj*	TK	ND	ND	ND	ND	ND	ND	ND	ND	ND	ND	ND	ND	ND	ND	ND	ND	ND	ND	ND	ND	ND

Table S1 continued

									CEF MIC		TET					AMP					NAL MIC		CIP MIC		ENR MIC		KAN MIC	
									Trial			MIC Trial					MIC Trial				Trial		Trial		Trial		Trial	
Isolate	Steer	Sampling time point	Incubation temp. (°C)	Treatment	Sampling date	Species	AMR	ST; CC	1	2	Level	1	2	3	4	Level	1	2	3	4	1	2	1	2	1	2	1	2
D6-32h-42-D	D6	32h	42	CCFA	6/3/16	*Cj*	TK	ND	ND	ND	ND	ND	ND	ND	ND	ND	ND	ND	ND	ND	ND	ND	ND	ND	ND	ND	ND	ND
D6-48h-42-A	D6	48h	42	CCFA	6/3/16	*Cj*	TK	**8576; 21**	ND	ND	ND	ND	ND	ND	ND	ND	ND	ND	ND	ND	ND	ND	ND	ND	ND	ND	ND	ND
D6-48h-42-B	D6	48h	42	CCFA	6/3/16	*Cj*	TK	ND	ND	ND	ND	ND	ND	ND	ND	ND	ND	ND	ND	ND	ND	ND	ND	ND	ND	ND	ND	ND
D6-48h-42-C	D6	48h	42	CCFA	6/3/16	*Cj*	TK	ND	ND	ND	ND	ND	ND	ND	ND	ND	ND	ND	ND	ND	ND	ND	ND	ND	ND	ND	ND	ND
D6-48h-42-D	D6	48h	42	CCFA	6/3/16	*Cj*	TK	ND	ND	ND	ND	ND	ND	ND	ND	ND	ND	ND	ND	ND	ND	ND	ND	ND	ND	ND	ND	ND
D6-72h-37-A	D6	72h	37	CCFA	6/4/16	*Cj*	TK	**8221; 61**	ND	ND	ND	ND	ND	ND	ND	ND	ND	ND	ND	ND	ND	ND	ND	ND	ND	ND	ND	ND
D6-72h-37-B	D6	72h	37	CCFA	6/4/16	*Cj*	TK	ND	ND	ND	ND	ND	ND	ND	ND	ND	ND	ND	ND	ND	ND	ND	ND	ND	ND	ND	ND	ND
D6-72h-37-E	D6	72h	37	CCFA	6/4/16	*Cj*	TK	ND	ND	ND	ND	ND	ND	ND	ND	ND	ND	ND	ND	ND	ND	ND	ND	ND	ND	ND	ND	ND
D6-72h-37-F	D6	72h	37	CCFA	6/4/16	*Cj*	TK	ND	ND	ND	ND	ND	ND	ND	ND	ND	ND	ND	ND	ND	ND	ND	ND	ND	ND	ND	ND	ND
D6-96h-37-B	D6	96h	37	CCFA	6/5/16	*Cj*	TK	ND	ND	ND	ND	ND	ND	ND	ND	ND	ND	ND	ND	ND	ND	ND	ND	ND	ND	ND	ND	ND
D6-96h-37-E	D6	96h	37	CCFA	6/5/16	*Cj*	TK	ND	ND	ND	ND	ND	ND	ND	ND	ND	ND	ND	ND	ND	ND	ND	ND	ND	ND	ND	ND	ND
D6-96h-37-F	D6	96h	37	CCFA	6/5/16	*Cj*	TK	ND	ND	ND	ND	ND	ND	ND	ND	ND	ND	ND	ND	ND	ND	ND	ND	ND	ND	ND	ND	ND
Campylobacter negative	D6	5d	37	CCFA	6/6/16	Cneg	N.A	ND	ND	ND	ND	ND	ND	ND	ND	ND	ND	ND	ND	ND	ND	ND	ND	ND	ND	ND	ND	ND
D6-6d-37-A	D6	6d	37	CCFA	6/7/16	*Cj*	TK	ND	ND	ND	ND	ND	ND	ND	ND	ND	ND	ND	ND	ND	ND	ND	ND	ND	ND	ND	ND	ND
D6-6d-37-B	D6	6d	37	CCFA	6/7/16	*Cj*	TK	ND	ND	ND	ND	ND	ND	ND	ND	ND	ND	ND	ND	ND	ND	ND	ND	ND	ND	ND	ND	ND
D6-6d-37-F	D6	6d	37	CCFA	6/7/16	*Cj*	TK	ND	ND	ND	ND	ND	ND	ND	ND	ND	ND	ND	ND	ND	ND	ND	ND	ND	ND	ND	ND	ND
Campylobacter negative	D6	7d	37	CCFA	6/8/16	Cneg	N.A	ND	ND	ND	ND	ND	ND	ND	ND	ND	ND	ND	ND	ND	ND	ND	ND	ND	ND	ND	ND	ND
D6-8d-37-A	D6	8d	37	CCFA	6/9/16	*Cj*	TK	ND	ND	ND	ND	ND	ND	ND	ND	ND	ND	ND	ND	ND	ND	ND	ND	ND	ND	ND	ND	ND
D6-8d-37-B	D6	8d	37	CCFA	6/9/16	*Cj*	TK	ND	ND	ND	ND	ND	ND	ND	ND	ND	ND	ND	ND	ND	ND	ND	ND	ND	ND	ND	ND	ND
D6-8d-37-C	D6	8d	37	CCFA	6/9/16	*Cj*	TK	ND	ND	ND	ND	ND	ND	ND	ND	ND	ND	ND	ND	ND	ND	ND	ND	ND	ND	ND	ND	ND
D6-8d-37-D	D6	8d	37	CCFA	6/9/16	*Cj*	TK	ND	ND	ND	ND	ND	ND	ND	ND	ND	ND	ND	ND	ND	ND	ND	ND	ND	ND	ND	ND	ND
D6-8d-37-E	D6	8d	37	CCFA	6/9/16	*Cj*	TK	ND	ND	ND	ND	ND	ND	ND	ND	ND	ND	ND	ND	ND	ND	ND	ND	ND	ND	ND	ND	ND
D6-8d-37-F	D6	8d	37	CCFA	6/9/16	*Cj*	TK	ND	ND	ND	ND	ND	ND	ND	ND	ND	ND	ND	ND	ND	ND	ND	ND	ND	ND	ND	ND	ND
D6-12d-37-A	D6	12d	37	CCFA	6/13/16	*Cj*	TK	ND	ND	ND	ND	ND	ND	ND	ND	ND	ND	ND	ND	ND	ND	ND	ND	ND	ND	ND	ND	ND
D6-12d-37-B	D6	12d	37	CCFA	6/13/16	*Cj*	TK	ND	ND	ND	ND	ND	ND	ND	ND	ND	ND	ND	ND	ND	ND	ND	ND	ND	ND	ND	ND	ND
D6-12d-37-C	D6	12d	37	CCFA	6/13/16	*Cj*	TK	ND	ND	ND	ND	ND	ND	ND	ND	ND	ND	ND	ND	ND	ND	ND	ND	ND	ND	ND	ND	ND
D6-12d-37-D	D6	12d	37	CCFA	6/13/16	*Cj*	TK	ND	ND	ND	ND	ND	ND	ND	ND	ND	ND	ND	ND	ND	ND	ND	ND	ND	ND	ND	ND	ND
D6-12d-37-E	D6	12d	37	CCFA	6/13/16	*Cj*	TK	ND	ND	ND	ND	ND	ND	ND	ND	ND	ND	ND	ND	ND	ND	ND	ND	ND	ND	ND	ND	ND
D6-12d-37-F	D6	12d	37	CCFA	6/13/16	*Cj*	TK	ND	ND	ND	ND	ND	ND	ND	ND	ND	ND	ND	ND	ND	ND	ND	ND	ND	ND	ND	ND	ND
D6-21d-37-A	D6	21d	37	CCFA	6/22/16	*Ch* †	N	ND	ND	ND	ND	ND	ND	ND	ND	ND	ND	ND	ND	ND	ND	ND	ND	ND	ND	ND	ND	ND
D6-21d-37-B	D6	21d	37	CCFA	6/22/16	*Cj*	TK	**8567; 21**	ND	ND	ND	ND	ND	ND	ND	ND	ND	ND	ND	ND	ND	ND	ND	ND	ND	ND	ND	ND
D6-21d-37-C	D6	21d	37	CCFA	6/22/16	*Cj*	TK	ND	ND	ND	ND	ND	ND	ND	ND	ND	ND	ND	ND	ND	ND	ND	ND	ND	ND	ND	ND	ND
D6-21d-37-D	D6	21d	37	CCFA	6/22/16	*Ch*	N	41 (Chy)	ND	ND	ND	ND	ND	ND	ND	ND	ND	ND	ND	ND	ND	ND	ND	ND	ND	ND	ND	ND
D6-28d-37-A	D6	28d	37	CCFA	6/29/16	*Ch*	N	41 (Chy)	ND	ND	ND	ND	ND	ND	ND	ND	ND	ND	ND	ND	ND	ND	ND	ND	ND	ND	ND	ND
Campylobacter negative	D6	35d	37	CCFA	7/6/16	Cneg	N.A	ND	ND	ND	ND	ND	ND	ND	ND	ND	ND	ND	ND	ND	ND	ND	ND	ND	ND	ND	ND	ND
Campylobacter negative	N1	0h	37	CHCL	6/24/16	Cneg	N.A	ND	ND	ND	ND	ND	ND	ND	ND	ND	ND	ND	ND	ND	ND	ND	ND	ND	ND	ND	ND	ND
N1-12h-37-A	N1	12h	37	CHCL	6/24/16	*Cj*	TK	**8567; 21**	ND	ND	ND	ND	ND	ND	ND	ND	ND	ND	ND	ND	ND	ND	ND	ND	ND	ND	ND	ND
N1-12h-37-B	N1	12h	37	CHCL	6/24/16	*Cj*	TK	ND	ND	ND	ND	ND	ND	ND	ND	ND	ND	ND	ND	ND	ND	ND	ND	ND	ND	ND	ND	ND
N1-12h-37-C	N1	12h	37	CHCL	6/24/16	*Cj*	TK	ND	ND	ND	ND	ND	ND	ND	ND	ND	ND	ND	ND	ND	ND	ND	ND	ND	ND	ND	ND	ND
N1-12h-37-D	N1	12h	37	CHCL	6/24/16	*Cj*	TK	ND	ND	ND	ND	ND	ND	ND	ND	ND	ND	ND	ND	ND	ND	ND	ND	ND	ND	ND	ND	ND
N1-12h-37-E	N1	12h	37	CHCL	6/24/16	*Cj*	TK	ND	ND	ND	ND	ND	ND	ND	ND	ND	ND	ND	ND	ND	ND	ND	ND	ND	ND	ND	ND	ND
N1-12h-37-F	N1	12h	37	CHCL	6/24/16	*Cj*	TK	ND	ND	ND	ND	ND	ND	ND	ND	ND	ND	ND	ND	ND	ND	ND	ND	ND	ND	ND	ND	ND
Campylobacter negative	N1	24h	37	CHCL	6/25/16	Cneg	N.A	ND	ND	ND	ND	ND	ND	ND	ND	ND	ND	ND	ND	ND	ND	ND	ND	ND	ND	ND	ND	ND
N1-36h-37-A	N1	36h	37	CHCL	6/25/16	*Cj*	TK	ND	ND	ND	ND	ND	ND	ND	ND	ND	ND	ND	ND	ND	ND	ND	ND	ND	ND	ND	ND	ND

Table S1 continued

									CEF MIC		TET					AMP					NAL MIC		CIP MIC		ENR MIC		KAN MIC	
												MIC					MIC											
									Trial			Trial					Trial				Trial		Trial		Trial		Trial	
Isolate	Steer	Sampling time point	Incubation temp. (°C)	Treatment	Sampling date	Species	AMR	ST; CC	1	2	Level	1	2	3	4	Level	1	2	3	4	1	2	1	2	1	2	1	2
N1-36h-37-B	N1	36h	37	CHCL	6/25/16	*Cj*	TK	ND	ND	ND	ND	ND	ND	ND	ND	ND	ND	ND	ND	ND	ND	ND	ND	ND	ND	ND	ND	ND
N1-36h-37-C	N1	36h	37	CHCL	6/25/16	*Cj*	TK	ND	ND	ND	ND	ND	ND	ND	ND	ND	ND	ND	ND	ND	ND	ND	ND	ND	ND	ND	ND	ND
N1-36h-37-D	N1	36h	37	CHCL	6/25/16	*Cj*	TK	ND	ND	ND	ND	ND	ND	ND	ND	ND	ND	ND	ND	ND	ND	ND	ND	ND	ND	ND	ND	ND
N1-36h-37-E	N1	36h	37	CHCL	6/25/16	*Cj*	TK	ND	ND	ND	ND	ND	ND	ND	ND	ND	ND	ND	ND	ND	ND	ND	ND	ND	ND	ND	ND	ND
N1-48h-37-A	N1	48h	37	CHCL	6/26/16	*Cj*	TK	ND	ND	ND	ND	ND	ND	ND	ND	ND	ND	ND	ND	ND	ND	ND	ND	ND	ND	ND	ND	ND
N1-48h-37-B	N1	48h	37	CHCL	6/26/16	*Cj*	TK	ND	ND	ND	ND	ND	ND	ND	ND	ND	ND	ND	ND	ND	ND	ND	ND	ND	ND	ND	ND	ND
N1-48h-37-C	N1	48h	37	CHCL	6/26/16	*Cj*	TK	ND	ND	ND	ND	ND	ND	ND	ND	ND	ND	ND	ND	ND	ND	ND	ND	ND	ND	ND	ND	ND
N1-48h-37-D	N1	48h	37	CHCL	6/26/16	*Cj*	TK	ND	ND	ND	ND	ND	ND	ND	ND	ND	ND	ND	ND	ND	ND	ND	ND	ND	ND	ND	ND	ND
N1-48h-37-E	N1	48h	37	CHCL	6/26/16	*Cj*	TK	ND	ND	ND	ND	ND	ND	ND	ND	ND	ND	ND	ND	ND	ND	ND	ND	ND	ND	ND	ND	ND
Campylobacter negative	N1	60h	37	CHCL	6/26/16	Cneg	N.A	ND	ND	ND	ND	ND	ND	ND	ND	ND	ND	ND	ND	ND	ND	ND	ND	ND	ND	ND	ND	ND
N1-72h-37-A	N1	72h	37	CHCL	6/27/16	*Cj*	TK	ND	ND	ND	ND	ND	ND	ND	ND	ND	ND	ND	ND	ND	ND	ND	ND	ND	ND	ND	ND	ND
N1-72h-37-B	N1	72h	37	CHCL	6/27/16	*Cj*	TK	ND	ND	ND	ND	ND	ND	ND	ND	ND	ND	ND	ND	ND	ND	ND	ND	ND	ND	ND	ND	ND
N1-96h-37-A	N1	96h	37	CHCL	6/28/16	*Cj*	TK	**8221; 61**	ND	ND	ND	ND	ND	ND	ND	ND	ND	ND	ND	ND	ND	ND	ND	ND	ND	ND	ND	ND
N1-96h-37-B	N1	96h	37	CHCL	6/28/16	*Cj*	TK	ND	ND	ND	ND	ND	ND	ND	ND	ND	ND	ND	ND	ND	ND	ND	ND	ND	ND	ND	ND	ND
N1-96h-37-C	N1	96h	37	CHCL	6/28/16	*Cj*	TK	ND	ND	ND	ND	ND	ND	ND	ND	ND	ND	ND	ND	ND	ND	ND	ND	ND	ND	ND	ND	ND
N1-96h-37-E	N1	96h	37	CHCL	6/28/16	*Cj*	TK	ND	ND	ND	ND	ND	ND	ND	ND	ND	ND	ND	ND	ND	ND	ND	ND	ND	ND	ND	ND	ND
Campylobacter negative	N1	5d	37	CHCL	6/29/16	Cneg	N.A	ND	ND	ND	ND	ND	ND	ND	ND	ND	ND	ND	ND	ND	ND	ND	ND	ND	ND	ND	ND	ND
Campylobacter negative	N1	6d	37	CHCL	6/30/16	Cneg	N.A	ND	ND	ND	ND	ND	ND	ND	ND	ND	ND	ND	ND	ND	ND	ND	ND	ND	ND	ND	ND	ND
Campylobacter negative	N1	7d	37	CHCL	7/1/16	Cneg	N.A	ND	ND	ND	ND	ND	ND	ND	ND	ND	ND	ND	ND	ND	ND	ND	ND	ND	ND	ND	ND	ND
Campylobacter negative	N1	8d	37	CHCL	7/2/16	Cneg	N.A	ND	ND	ND	ND	ND	ND	ND	ND	ND	ND	ND	ND	ND	ND	ND	ND	ND	ND	ND	ND	ND
Campylobacter negative	N1	9d	37	CHCL	7/3/16	Cneg	N.A	ND	ND	ND	ND	ND	ND	ND	ND	ND	ND	ND	ND	ND	ND	ND	ND	ND	ND	ND	ND	ND
Campylobacter negative	N1	10d	37	CHCL	7/4/16	Cneg	N.A	ND	ND	ND	ND	ND	ND	ND	ND	ND	ND	ND	ND	ND	ND	ND	ND	ND	ND	ND	ND	ND
Campylobacter negative	N1	18d	37	CHCL	7/12/16	Cneg	N.A	ND	ND	ND	ND	ND	ND	ND	ND	ND	ND	ND	ND	ND	ND	ND	ND	ND	ND	ND	ND	ND
Campylobacter negative	N2	0h	37	CHCL	6/24/16	Cneg	N.A	ND	ND	ND	ND	ND	ND	ND	ND	ND	ND	ND	ND	ND	ND	ND	ND	ND	ND	ND	ND	ND
Campylobacter negative	N2	12h	37	CHCL	6/24/16	Cneg	N.A	ND	ND	ND	ND	ND	ND	ND	ND	ND	ND	ND	ND	ND	ND	ND	ND	ND	ND	ND	ND	ND
Campylobacter negative	N2	24h	37	CHCL	6/25/16	Cneg	N.A	ND	ND	ND	ND	ND	ND	ND	ND	ND	ND	ND	ND	ND	ND	ND	ND	ND	ND	ND	ND	ND
N2-36h-37-A	N2	36h	37	CHCL	6/25/16	*Cj*	PS	ND	ND	ND	ND	ND	ND	ND	ND	ND	ND	ND	ND	ND	ND	ND	ND	ND	ND	ND	ND	ND
N2-36h-37-B	N2	36h	37	CHCL	6/25/16	*Cj*	TK	**8221; 61**	ND	ND	ND	ND	ND	ND	ND	ND	ND	ND	ND	ND	ND	ND	ND	ND	ND	ND	ND	ND
N2-36h-37-C	N2	36h	37	CHCL	6/25/16	*Cj*	PS	21; 21	ND	ND	ND	ND	ND	ND	ND	ND	ND	ND	ND	ND	ND	ND	ND	ND	ND	ND	ND	ND
N2-48h-37-A	N2	48h	37	CHCL	6/26/16	*Cj*	TK	ND	ND	ND	ND	ND	ND	ND	ND	ND	ND	ND	ND	ND	ND	ND	ND	ND	ND	ND	ND	ND
N2-48h-37-B	N2	48h	37	CHCL	6/26/16	*Cj*	PS	ND	ND	ND	ND	ND	ND	ND	ND	ND	ND	ND	ND	ND	ND	ND	ND	ND	ND	ND	ND	ND
N2-48h-37-C	N2	48h	37	CHCL	6/26/16	*Cj*	TK	ND	ND	ND	ND	ND	ND	ND	ND	ND	ND	ND	ND	ND	ND	ND	ND	ND	ND	ND	ND	ND
Campylobacter negative	N2	60h	37	CHCL	6/26/16	Cneg	N.A	ND	ND	ND	ND	ND	ND	ND	ND	ND	ND	ND	ND	ND	ND	ND	ND	ND	ND	ND	ND	ND
N2-72h-37-A	N2	72h	37	CHCL	6/27/16	*Cj*	TK	ND	ND	ND	ND	ND	ND	ND	ND	ND	ND	ND	ND	ND	ND	ND	ND	ND	ND	ND	ND	ND
N2-72h-37-B	N2	72h	37	CHCL	6/27/16	*Cj*	PS	21; 21	ND	ND	ND	ND	ND	ND	ND	ND	ND	ND	ND	ND	ND	ND	ND	ND	ND	ND	ND	ND
N2-72h-37-C	N2	72h	37	CHCL	6/27/16	*Cj*	TK	ND	ND	ND	ND	ND	ND	ND	ND	ND	ND	ND	ND	ND	ND	ND	ND	ND	ND	ND	ND	ND
Campylobacter negative	N2	96h	37	CHCL	6/28/16	Cneg	N.A	ND	ND	ND	ND	ND	ND	ND	ND	ND	ND	ND	ND	ND	ND	ND	ND	ND	ND	ND	ND	ND
N2-5d-37-A	N2	5d	37	CHCL	6/29/16	*Cj*	TK	ND	ND	ND	ND	ND	ND	ND	ND	ND	ND	ND	ND	ND	ND	ND	ND	ND	ND	ND	ND	ND
N2-6d-37-A	N2	6d	37	CHCL	6/30/16	*Cj*	TK	ND	ND	ND	ND	ND	ND	ND	ND	ND	ND	ND	ND	ND	ND	ND	ND	ND	ND	ND	ND	ND
N2-7d-37-A	N2	7d	37	CHCL	7/1/16	*Cj*	TK	**8221; 61**	ND	ND	ND	ND	ND	ND	ND	ND	ND	ND	ND	ND	ND	ND	ND	ND	ND	ND	ND	ND
N2-7d-37-B	N2	7d	37	CHCL	7/1/16	*Cj*	TK	ND	ND	ND	ND	ND	ND	ND	ND	ND	ND	ND	ND	ND	ND	ND	ND	ND	ND	ND	ND	ND
N2-7d-37-C	N2	7d	37	CHCL	7/1/16	*Cj*	TK	ND	ND	ND	ND	ND	ND	ND	ND	ND	ND	ND	ND	ND	ND	ND	ND	ND	ND	ND	ND	ND
N2-7d-37-E	N2	7d	37	CHCL	7/1/16	*Cj*	TK	ND	ND	ND	ND	ND	ND	ND	ND	ND	ND	ND	ND	ND	ND	ND	ND	ND	ND	ND	ND	ND
Campylobacter negative	N2	8d	37	CHCL	7/2/16	Cneg	N.A	ND	ND	ND	ND	ND	ND	ND	ND	ND	ND	ND	ND	ND	ND	ND	ND	ND	ND	ND	ND	ND

Table S1 continued

									CEF MIC		TET					AMP					NAL MIC		CIP MIC		ENR MIC		KAN MIC	
									Trial			MIC Trial					MIC Trial				Trial		Trial		Trial		Trial	
Isolate	Steer	Sampling time point	Incubation temp. (°C)	Treatment	Sampling date	Species	AMR	ST; CC	1	2	Level	1	2	3	4	Level	1	2	3	4	1	2	1	2	1	2	1	2
Campylobacter negative	N2	9d	37	CHCL	7/3/16	Cneg	N.A	ND	ND	ND	ND	ND	ND	ND	ND	ND	ND	ND	ND	ND	ND	ND	ND	ND	ND	ND	ND	ND
Campylobacter negative	N2	10d	37	CHCL	7/4/16	Cneg	N.A	ND	ND	ND	ND	ND	ND	ND	ND	ND	ND	ND	ND	ND	ND	ND	ND	ND	ND	ND	ND	ND
Campylobacter negative	N2	18d	37	CHCL	7/12/16	Cneg	N.A	ND	ND	ND	ND	ND	ND	ND	ND	ND	ND	ND	ND	ND	ND	ND	ND	ND	ND	ND	ND	ND
Campylobacter negative	N3	0h	37	CHCL	6/24/16	Cneg	N.A	ND	ND	ND	ND	ND	ND	ND	ND	ND	ND	ND	ND	ND	ND	ND	ND	ND	ND	ND	ND	ND
N3-12h-37-A	N3	12h	37	CHCL	6/24/16	*Cj*	TK	**8221; 61**	ND	ND	ND	ND	ND	ND	ND	ND	ND	ND	ND	ND	ND	ND	ND	ND	ND	ND	ND	ND
N3-12h-37-B	N3	12h	37	CHCL	6/24/16	*Cj*	TK	ND	ND	ND	ND	ND	ND	ND	ND	ND	ND	ND	ND	ND	ND	ND	ND	ND	ND	ND	ND	ND
N3-12h-37-C	N3	12h	37	CHCL	6/24/16	*Cj*	TK	ND	ND	ND	ND	ND	ND	ND	ND	ND	ND	ND	ND	ND	ND	ND	ND	ND	ND	ND	ND	ND
N3-12h-37-D	N3	12h	37	CHCL	6/24/16	*Cj*	TK	ND	ND	ND	ND	ND	ND	ND	ND	ND	ND	ND	ND	ND	ND	ND	ND	ND	ND	ND	ND	ND
N3-12h-37-E	N3	12h	37	CHCL	6/24/16	*Cj*	TK	ND	ND	ND	ND	ND	ND	ND	ND	ND	ND	ND	ND	ND	ND	ND	ND	ND	ND	ND	ND	ND
N3-12h-37-F	N3	12h	37	CHCL	6/24/16	*Cj*	TK	ND	ND	ND	ND	ND	ND	ND	ND	ND	ND	ND	ND	ND	ND	ND	ND	ND	ND	ND	ND	ND
Campylobacter negative	N3	24h	37	CHCL	6/25/16	Cneg	N.A	ND	ND	ND	ND	ND	ND	ND	ND	ND	ND	ND	ND	ND	ND	ND	ND	ND	ND	ND	ND	ND
Campylobacter negative	N3	36h	37	CHCL	6/25/16	Cneg	N.A	ND	ND	ND	ND	ND	ND	ND	ND	ND	ND	ND	ND	ND	ND	ND	ND	ND	ND	ND	ND	ND
Campylobacter negative	N3	48h	37	CHCL	6/26/16	Cneg	N.A	ND	ND	ND	ND	ND	ND	ND	ND	ND	ND	ND	ND	ND	ND	ND	ND	ND	ND	ND	ND	ND
N3-60h-37-A	N3	60h	37	CHCL	6/26/16	*Cj*	TK	ND	ND	ND	ND	ND	ND	ND	ND	ND	ND	ND	ND	ND	ND	ND	ND	ND	ND	ND	ND	ND
N3-60h-37-B	N3	60h	37	CHCL	6/26/16	*Cj*	PS	21; 21	ND	ND	ND	ND	ND	ND	ND	ND	ND	ND	ND	ND	ND	ND	ND	ND	ND	ND	ND	ND
N3-72h-37-A	N3	72h	37	CHCL	6/27/16	*Cj*	TK	ND	ND	ND	ND	ND	ND	ND	ND	ND	ND	ND	ND	ND	ND	ND	ND	ND	ND	ND	ND	ND
N3-72h-37-B	N3	72h	37	CHCL	6/27/16	*Cj*	TK	ND	ND	ND	ND	ND	ND	ND	ND	ND	ND	ND	ND	ND	ND	ND	ND	ND	ND	ND	ND	ND
N3-72h-37-D	N3	72h	37	CHCL	6/27/16	*Cj*	TK	ND	ND	ND	ND	ND	ND	ND	ND	ND	ND	ND	ND	ND	ND	ND	ND	ND	ND	ND	ND	ND
N3-72h-37-E	N3	72h	37	CHCL	6/27/16	*Cj*	TK	ND	ND	ND	ND	ND	ND	ND	ND	ND	ND	ND	ND	ND	ND	ND	ND	ND	ND	ND	ND	ND
Campylobacter negative	N3	6d	37	CHCL	6/30/16	Cneg	N.A	ND	ND	ND	ND	ND	ND	ND	ND	ND	ND	ND	ND	ND	ND	ND	ND	ND	ND	ND	ND	ND
Campylobacter negative	N3	7d	37	CHCL	7/1/16	Cneg	N.A	ND	ND	ND	ND	ND	ND	ND	ND	ND	ND	ND	ND	ND	ND	ND	ND	ND	ND	ND	ND	ND
Campylobacter negative	N3	8d	37	CHCL	7/2/16	Cneg	N.A	ND	ND	ND	ND	ND	ND	ND	ND	ND	ND	ND	ND	ND	ND	ND	ND	ND	ND	ND	ND	ND
Campylobacter negative	N3	9d	37	CHCL	7/3/16	Cneg	N.A	ND	ND	ND	ND	ND	ND	ND	ND	ND	ND	ND	ND	ND	ND	ND	ND	ND	ND	ND	ND	ND
N3-10d-37-A	N3	10d	37	CHCL	7/4/16	*Cj*	TK	**8221; 61**	ND	ND	ND	ND	ND	ND	ND	ND	ND	ND	ND	ND	ND	ND	ND	ND	ND	ND	ND	ND
Campylobacter negative	N3	18d	37	CHCL	7/12/16	Cneg	N.A	ND	ND	ND	ND	ND	ND	ND	ND	ND	ND	ND	ND	ND	ND	ND	ND	ND	ND	ND	ND	ND
N4-0h-37-A	N4	0h	37	CHCL	6/24/16	*Cj*	PS	21; 21	20	20	ND	1	1	1	1	ND	32	32	>32	>32	4	4	1	1	1	1	1	16
Campylobacter negative	N4	12h	37	CHCL	6/24/16	Cneg	N.A	ND	ND	ND	ND	ND	ND	ND	ND	ND	ND	ND	ND	ND	ND	ND	ND	ND	ND	ND	ND	ND
Campylobacter negative	N4	24h	37	CHCL	6/25/16	Cneg	N.A	ND	ND	ND	ND	ND	ND	ND	ND	ND	ND	ND	ND	ND	ND	ND	ND	ND	ND	ND	ND	ND
N4-36h-37-A	N4	36h	37	CHCL	6/25/16	*Cj*	TK	**8221; 61**	ND	ND	ND	ND	ND	ND	ND	ND	ND	ND	ND	ND	ND	ND	ND	ND	ND	ND	ND	ND
N4-36h-37-B	N4	36h	37	CHCL	6/25/16	*Cj*	TK	ND	ND	ND	ND	ND	ND	ND	ND	ND	ND	ND	ND	ND	ND	ND	ND	ND	ND	ND	ND	ND
N4-36h-37-C	N4	36h	37	CHCL	6/25/16	*Cj*	TK	ND	ND	ND	ND	ND	ND	ND	ND	ND	ND	ND	ND	ND	ND	ND	ND	ND	ND	ND	ND	ND
N4-36h-37-D	N4	36h	37	CHCL	6/25/16	*Cj*	TK	ND	ND	ND	ND	ND	ND	ND	ND	ND	ND	ND	ND	ND	ND	ND	ND	ND	ND	ND	ND	ND
N4-36h-37-E	N4	36h	37	CHCL	6/25/16	*Cj*	TK	ND	ND	ND	ND	ND	ND	ND	ND	ND	ND	ND	ND	ND	ND	ND	ND	ND	ND	ND	ND	ND
N4-36h-37-F	N4	36h	37	CHCL	6/25/16	*Cj*	TK	ND	ND	ND	ND	ND	ND	ND	ND	ND	ND	ND	ND	ND	ND	ND	ND	ND	ND	ND	ND	ND
N4-48h-37-A	N4	48h	37	CHCL	6/26/16	*Cj*	TK	ND	ND	ND	ND	ND	ND	ND	ND	ND	ND	ND	ND	ND	ND	ND	ND	ND	ND	ND	ND	ND
N4-48h-37-B	N4	48h	37	CHCL	6/26/16	*Cj*	TK	ND	ND	ND	ND	ND	ND	ND	ND	ND	ND	ND	ND	ND	ND	ND	ND	ND	ND	ND	ND	ND
N4-48h-37-C	N4	48h	37	CHCL	6/26/16	*Cj*	PS	21; 21	20	20	ND	>128	>128	64	64	ND	32	32	>32	>32	8	8	1	1	1	1	>256	>256
N4-48h-37-D	N4	48h	37	CHCL	6/26/16	*Cj*	TK	ND	ND	ND	ND	ND	ND	ND	ND	ND	ND	ND	ND	ND	ND	ND	ND	ND	ND	ND	ND	ND
N4-48h-37-E	N4	48h	37	CHCL	6/26/16	*Cj*	TK	ND	ND	ND	ND	ND	ND	ND	ND	ND	ND	ND	ND	ND	ND	ND	ND	ND	ND	ND	ND	ND
N4-48h-37-F	N4	48h	37	CHCL	6/26/16	*Cj*	TK	ND	ND	ND	ND	ND	ND	ND	ND	ND	ND	ND	ND	ND	ND	ND	ND	ND	ND	ND	ND	ND
Campylobacter negative	N4	60h	37	CHCL	6/26/16	Cneg	N.A	ND	ND	ND	ND	ND	ND	ND	ND	ND	ND	ND	ND	ND	ND	ND	ND	ND	ND	ND	ND	ND
N4-72h-37-A	N4	72h	37	CHCL	6/27/16	*Cj*	TK	ND	ND	ND	ND	ND	ND	ND	ND	ND	ND	ND	ND	ND	ND	ND	ND	ND	ND	ND	ND	ND
N4-72h-37-B	N4	72h	37	CHCL	6/27/16	*Cj*	TK	ND	ND	ND	ND	ND	ND	ND	ND	ND	ND	ND	ND	ND	ND	ND	ND	ND	ND	ND	ND	ND
N4-72h-37-C	N4	72h	37	CHCL	6/27/16	*Cj*	TK	ND	ND	ND	ND	ND	ND	ND	ND	ND	ND	ND	ND	ND	ND	ND	ND	ND	ND	ND	ND	ND

Table S1 continued

									CEF MIC		TET					AMP					NAL MIC		CIP MIC		ENR MIC		KAN MIC	
												MIC					MIC											
									Trial			Trial					Trial				Trial		Trial		Trial		Trial	
Isolate	Steer	Sampling time point	Incubation temp. (°C)	Treatment	Sampling date	Species	AMR	ST; CC	1	2	Level	1	2	3	4	Level	1	2	3	4	1	2	1	2	1	2	1	2
N4-72h-37-D	N4	72h	37	CHCL	6/27/16	*Cj*	TK	ND	ND	ND	ND	ND	ND	ND	ND	ND	ND	ND	ND	ND	ND	ND	ND	ND	ND	ND	ND	ND
N4-72h-37-F	N4	72h	37	CHCL	6/27/16	*Cj*	TK	ND	ND	ND	ND	ND	ND	ND	ND	ND	ND	ND	ND	ND	ND	ND	ND	ND	ND	ND	ND	ND
N4-96h-37-A	N4	96h	37	CHCL	6/28/16	*Cj*	TK	ND	ND	ND	ND	ND	ND	ND	ND	ND	ND	ND	ND	ND	ND	ND	ND	ND	ND	ND	ND	ND
N4-5d-37-A	N4	5d	37	CHCL	6/29/16	*Ch* †	TN	ND	ND	ND	ND	ND	ND	ND	ND	ND	ND	ND	ND	ND	ND	ND	ND	ND	ND	ND	ND	ND
N4-6d-37-A	N4	6d	37	CHCL	6/30/16	*Cj*	TK	ND	ND	ND	ND	ND	ND	ND	ND	ND	ND	ND	ND	ND	ND	ND	ND	ND	ND	ND	ND	ND
N4-6d-37-B	N4	6d	37	CHCL	6/30/16	*Cj*	TK	ND	ND	ND	ND	ND	ND	ND	ND	ND	ND	ND	ND	ND	ND	ND	ND	ND	ND	ND	ND	ND
N4-7d-37-A	N4	7d	37	CHCL	7/1/16	*Cj*	TK	ND	ND	ND	ND	ND	ND	ND	ND	ND	ND	ND	ND	ND	ND	ND	ND	ND	ND	ND	ND	ND
N4-7d-37-B	N4	7d	37	CHCL	7/1/16	*Cj*	TK	ND	ND	ND	ND	ND	ND	ND	ND	ND	ND	ND	ND	ND	ND	ND	ND	ND	ND	ND	ND	ND
N4-7d-37-C	N4	7d	37	CHCL	7/1/16	*Cj*	TK	ND	ND	ND	ND	ND	ND	ND	ND	ND	ND	ND	ND	ND	ND	ND	ND	ND	ND	ND	ND	ND
N4-7d-37-D	N4	7d	37	CHCL	7/1/16	*Cj*	TK	ND	ND	ND	ND	ND	ND	ND	ND	ND	ND	ND	ND	ND	ND	ND	ND	ND	ND	ND	ND	ND
N4-7d-37-E	N4	7d	37	CHCL	7/1/16	*Cj*	TK	ND	ND	ND	ND	ND	ND	ND	ND	ND	ND	ND	ND	ND	ND	ND	ND	ND	ND	ND	ND	ND
N4-7d-37-F	N4	7d	37	CHCL	7/1/16	*Cj*	TK	ND	ND	ND	ND	ND	ND	ND	ND	ND	ND	ND	ND	ND	ND	ND	ND	ND	ND	ND	ND	ND
N4-8d-37-A	N4	8d	37	CHCL	7/2/16	*Cj*	TK	ND	ND	ND	ND	ND	ND	ND	ND	ND	ND	ND	ND	ND	ND	ND	ND	ND	ND	ND	ND	ND
N4-8d-37-B	N4	8d	37	CHCL	7/2/16	*Cj*	TK	ND	ND	ND	ND	ND	ND	ND	ND	ND	ND	ND	ND	ND	ND	ND	ND	ND	ND	ND	ND	ND
N4-8d-37-C	N4	8d	37	CHCL	7/2/16	*Cj*	TK	ND	ND	ND	ND	ND	ND	ND	ND	ND	ND	ND	ND	ND	ND	ND	ND	ND	ND	ND	ND	ND
N4-8d-37-D	N4	8d	37	CHCL	7/2/16	*Cj*	TK	ND	ND	ND	ND	ND	ND	ND	ND	ND	ND	ND	ND	ND	ND	ND	ND	ND	ND	ND	ND	ND
N4-8d-37-E	N4	8d	37	CHCL	7/2/16	*Cj*	TK	ND	ND	ND	ND	ND	ND	ND	ND	ND	ND	ND	ND	ND	ND	ND	ND	ND	ND	ND	ND	ND
N4-8d-37-F	N4	8d	37	CHCL	7/2/16	*Cj*	TK	ND	ND	ND	ND	ND	ND	ND	ND	ND	ND	ND	ND	ND	ND	ND	ND	ND	ND	ND	ND	ND
N4-9d-37-A	N4	9d	37	CHCL	7/3/16	*Cj*	TK	**8567; 21**	ND	ND	ND	ND	ND	ND	ND	ND	ND	ND	ND	ND	ND	ND	ND	ND	ND	ND	ND	ND
N4-9d-37-B	N4	9d	37	CHCL	7/3/16	*Cj*	TK	ND	ND	ND	ND	ND	ND	ND	ND	ND	ND	ND	ND	ND	ND	ND	ND	ND	ND	ND	ND	ND
N4-9d-37-C	N4	9d	37	CHCL	7/3/16	*Cj*	TK	ND	ND	ND	ND	ND	ND	ND	ND	ND	ND	ND	ND	ND	ND	ND	ND	ND	ND	ND	ND	ND
N4-9d-37-D	N4	9d	37	CHCL	7/3/16	*Cj*	TK	ND	ND	ND	ND	ND	ND	ND	ND	ND	ND	ND	ND	ND	ND	ND	ND	ND	ND	ND	ND	ND
N4-9d-37-E	N4	9d	37	CHCL	7/3/16	*Cj*	TK	ND	ND	ND	ND	ND	ND	ND	ND	ND	ND	ND	ND	ND	ND	ND	ND	ND	ND	ND	ND	ND
N4-9d-37-F	N4	9d	37	CHCL	7/3/16	*Cj*	TK	ND	ND	ND	ND	ND	ND	ND	ND	ND	ND	ND	ND	ND	ND	ND	ND	ND	ND	ND	ND	ND
Campylobacter negative	N4	10d	37	CHCL	7/4/16	Cneg	N.A	ND	ND	ND	ND	ND	ND	ND	ND	ND	ND	ND	ND	ND	ND	ND	ND	ND	ND	ND	ND	ND
Campylobacter negative	N4	18d	37	CHCL	7/12/16	Cneg	N.A	ND	ND	ND	ND	ND	ND	ND	ND	ND	ND	ND	ND	ND	ND	ND	ND	ND	ND	ND	ND	ND
N5-0h-37-A	N5	0h	37	CHCL	6/29/16	*Cj*	TK	**8221; 61**	20	20	A	>128	>128	128	128	A	8	8	>32	>32	8	8	1	1	1	1	>256	>256
N5-0h-37-B	N5	0h	37	CHCL	6/29/16	*Cj*	TK	ND	ND	ND	ND	ND	ND	ND	ND	ND	ND	ND	ND	ND	ND	ND	ND	ND	ND	ND	ND	ND
N5-0h-37-C	N5	0h	37	CHCL	6/29/16	*Cj*	TK	ND	ND	ND	ND	ND	ND	ND	ND	ND	ND	ND	ND	ND	ND	ND	ND	ND	ND	ND	ND	ND
N5-0h-37-E	N5	0h	37	CHCL	6/29/16	*Cj*	TK	ND	ND	ND	ND	ND	ND	ND	ND	ND	ND	ND	ND	ND	ND	ND	ND	ND	ND	ND	ND	ND
N5-0h-37-F	N5	0h	37	CHCL	6/29/16	*Cj*	TK	ND	ND	ND	ND	ND	ND	ND	ND	ND	ND	ND	ND	ND	ND	ND	ND	ND	ND	ND	ND	ND
Campylobacter negative	N5	12h	37	CHCL	6/29/16	Cneg	N.A	ND	ND	ND	ND	ND	ND	ND	ND	ND	ND	ND	ND	ND	ND	ND	ND	ND	ND	ND	ND	ND
Campylobacter negative	N5	24h	37	CHCL	6/30/16	Cneg	N.A	ND	ND	ND	ND	ND	ND	ND	ND	ND	ND	ND	ND	ND	ND	ND	ND	ND	ND	ND	ND	ND
Campylobacter negative	N5	36h	37	CHCL	6/30/16	Cneg	N.A	ND	ND	ND	ND	ND	ND	ND	ND	ND	ND	ND	ND	ND	ND	ND	ND	ND	ND	ND	ND	ND
Campylobacter negative	N5	48h	37	CHCL	7/1/16	Cneg	N.A	ND	ND	ND	ND	ND	ND	ND	ND	ND	ND	ND	ND	ND	ND	ND	ND	ND	ND	ND	ND	ND
Campylobacter negative	N5	60h	37	CHCL	7/1/16	Cneg	N.A	ND	ND	ND	ND	ND	ND	ND	ND	ND	ND	ND	ND	ND	ND	ND	ND	ND	ND	ND	ND	ND
Campylobacter negative	N5	72h	37	CHCL	7/2/16	Cneg	N.A	ND	ND	ND	ND	ND	ND	ND	ND	ND	ND	ND	ND	ND	ND	ND	ND	ND	ND	ND	ND	ND
Campylobacter negative	N5	96h	37	CHCL	7/2/16	Cneg	N.A	ND	ND	ND	ND	ND	ND	ND	ND	ND	ND	ND	ND	ND	ND	ND	ND	ND	ND	ND	ND	ND
N5-5d-37-B	N5	5d	37	CHCL	7/4/16	*Cj*	TK	**8221; 61**	20	20	A	>128	>128	128	128	B	16	16	32	32	8	8	1	1	1	1	>256	>256
N5-5d-37-C	N5	5d	37	CHCL	7/4/16	*Cj*	TK	ND	ND	ND	ND	ND	ND	ND	ND	ND	ND	ND	ND	ND	ND	ND	ND	ND	ND	ND	ND	ND
N5-5d-37-D	N5	5d	37	CHCL	7/4/16	*Cj*	TK	ND	ND	ND	ND	ND	ND	ND	ND	ND	ND	ND	ND	ND	ND	ND	ND	ND	ND	ND	ND	ND
N5-5d-37-E	N5	5d	37	CHCL	7/4/16	*Cj*	TK	ND	ND	ND	ND	ND	ND	ND	ND	ND	ND	ND	ND	ND	ND	ND	ND	ND	ND	ND	ND	ND
N5-5d-37-F	N5	5d	37	CHCL	7/4/16	*Cj*	TK	ND	ND	ND	ND	ND	ND	ND	ND	ND	ND	ND	ND	ND	ND	ND	ND	ND	ND	ND	ND	ND
Campylobacter negative	N5	6d	37	CHCL	7/5/16	Cneg	N.A	ND	ND	ND	ND	ND	ND	ND	ND	ND	ND	ND	ND	ND	ND	ND	ND	ND	ND	ND	ND	ND

Table S1 continued

									CEF MIC		TET					AMP					NAL MIC		CIP MIC		ENR MIC		KAN MIC	
									Trial		Level	MIC Trial				Level	MIC Trial				Trial		Trial		Trial		Trial	
Isolate	Steer	Sampling time point	Incubation temp. (°C)	Treatment	Sampling date	Species	AMR	ST; CC	1	2		1	2	3	4		1	2	3	4	1	2	1	2	1	2	1	2
Campylobacter negative	N5	7d	37	CHCL	7/6/16	Cneg	N.A	ND	ND	ND	ND	ND	ND	ND	ND	ND	ND	ND	ND	ND	ND	ND	ND	ND	ND	ND	ND	ND
Campylobacter negative	N5	8d	37	CHCL	7/7/16	Cneg	N.A	ND	ND	ND	ND	ND	ND	ND	ND	ND	ND	ND	ND	ND	ND	ND	ND	ND	ND	ND	ND	ND
Campylobacter negative	N5	9d	37	CHCL	7/8/16	Cneg	N.A	ND	ND	ND	ND	ND	ND	ND	ND	ND	ND	ND	ND	ND	ND	ND	ND	ND	ND	ND	ND	ND
Campylobacter negative	N5	10d	37	CHCL	7/9/16	Cneg	N.A	ND	ND	ND	ND	ND	ND	ND	ND	ND	ND	ND	ND	ND	ND	ND	ND	ND	ND	ND	ND	ND
Campylobacter negative	N5	13d	37	CHCL	7/12/16	Cneg	N.A	ND	ND	ND	ND	ND	ND	ND	ND	ND	ND	ND	ND	ND	ND	ND	ND	ND	ND	ND	ND	ND
N6-0h-37-A	N6	0h	37	CHCL	6/29/16	*Cj*	PS	21; 21	20	20	B	1	1	1	1	A	32	32	>32	>32	4	4	1	1	1	1	1	1
N6-0h-37-B	N6	0h	37	CHCL	6/29/16	*Cj*	PS	ND	20	20	ND	1	1	ND	ND	ND	32	32	ND	ND	4	4	1	1	1	1	1	1
N6-0h-37-C	N6	0h	37	CHCL	6/29/16	*Cj*	PS	ND	ND	ND	ND	ND	ND	ND	ND	ND	ND	ND	ND	ND	ND	ND	ND	ND	ND	ND	ND	ND
N6-0h-37-D	N6	0h	37	CHCL	6/29/16	*Cj*	PS	ND	ND	ND	ND	ND	ND	ND	ND	ND	ND	ND	ND	ND	ND	ND	ND	ND	ND	ND	ND	ND
N6-0h-37-E	N6	0h	37	CHCL	6/29/16	*Cj*	PS	ND	ND	ND	ND	ND	ND	ND	ND	ND	ND	ND	ND	ND	ND	ND	ND	ND	ND	ND	ND	ND
N6-0h-37-F	N6	0h	37	CHCL	6/29/16	*Cj*	PS	ND	ND	ND	ND	ND	ND	ND	ND	ND	ND	ND	ND	ND	ND	ND	ND	ND	ND	ND	ND	ND
Campylobacter negative	N6	12h	37	CHCL	6/29/16	Cneg	N.A	ND	ND	ND	ND	ND	ND	ND	ND	ND	ND	ND	ND	ND	ND	ND	ND	ND	ND	ND	ND	ND
Campylobacter negative	N6	24h	37	CHCL	6/30/16	Cneg	N.A	ND	ND	ND	ND	ND	ND	ND	ND	ND	ND	ND	ND	ND	ND	ND	ND	ND	ND	ND	ND	ND
Campylobacter negative	N6	36h	37	CHCL	6/30/16	Cneg	N.A	ND	ND	ND	ND	ND	ND	ND	ND	ND	ND	ND	ND	ND	ND	ND	ND	ND	ND	ND	ND	ND
Campylobacter negative	N6	48h	37	CHCL	7/1/16	Cneg	N.A	ND	ND	ND	ND	ND	ND	ND	ND	ND	ND	ND	ND	ND	ND	ND	ND	ND	ND	ND	ND	ND
Campylobacter negative	N6	60h	37	CHCL	7/1/16	Cneg	N.A	ND	ND	ND	ND	ND	ND	ND	ND	ND	ND	ND	ND	ND	ND	ND	ND	ND	ND	ND	ND	ND
Campylobacter negative	N6	72h	37	CHCL	7/2/16	Cneg	N.A	ND	ND	ND	ND	ND	ND	ND	ND	ND	ND	ND	ND	ND	ND	ND	ND	ND	ND	ND	ND	ND
Campylobacter negative	N6	96h	37	CHCL	7/2/16	Cneg	N.A	ND	ND	ND	ND	ND	ND	ND	ND	ND	ND	ND	ND	ND	ND	ND	ND	ND	ND	ND	ND	ND
Campylobacter negative	N6	5d	37	CHCL	7/4/16	Cneg	N.A	ND	ND	ND	ND	ND	ND	ND	ND	ND	ND	ND	ND	ND	ND	ND	ND	ND	ND	ND	ND	ND
Campylobacter negative	N6	6d	37	CHCL	7/5/16	Cneg	N.A	ND	ND	ND	ND	ND	ND	ND	ND	ND	ND	ND	ND	ND	ND	ND	ND	ND	ND	ND	ND	ND
Campylobacter negative	N6	7d	37	CHCL	7/6/16	Cneg	N.A	ND	ND	ND	ND	ND	ND	ND	ND	ND	ND	ND	ND	ND	ND	ND	ND	ND	ND	ND	ND	ND
Campylobacter negative	N6	8d	37	CHCL	7/7/16	Cneg	N.A	ND	ND	ND	ND	ND	ND	ND	ND	ND	ND	ND	ND	ND	ND	ND	ND	ND	ND	ND	ND	ND
Campylobacter negative	N6	9d	37	CHCL	7/8/16	Cneg	N.A	ND	ND	ND	ND	ND	ND	ND	ND	ND	ND	ND	ND	ND	ND	ND	ND	ND	ND	ND	ND	ND
Campylobacter negative	N6	10d	37	CHCL	7/9/16	Cneg	N.A	ND	ND	ND	ND	ND	ND	ND	ND	ND	ND	ND	ND	ND	ND	ND	ND	ND	ND	ND	ND	ND
N6-13d-37-A	N6	13d	37	CHCL	7/12/16	*Cj*	PS	797*; 21	20	20	B	1	1	1	1	A	32	32	>32	>32	4	4	1	1	1	1	1	1
N6-13d-37-B	N6	13d	37	CHCL	7/12/16	*Cj*	PS	ND	20	20	ND	1	1	ND	ND	ND	32	32	ND	ND	4	4	1	1	1	1	1	1
N6-13d-37-C	N6	13d	37	CHCL	7/12/16	*Cj*	TK	**8221; 61**	ND	ND	ND	ND	ND	ND	ND	ND	ND	ND	ND	ND	ND	ND	ND	ND	ND	ND	ND	ND
N6-13d-37-E	N6	13d	37	CHCL	7/12/16	*Cj*	TK	ND	ND	ND	ND	ND	ND	ND	ND	ND	ND	ND	ND	ND	ND	ND	ND	ND	ND	ND	ND	ND
N6-13d-37-F	N6	13d	37	CHCL	7/12/16	*Cj*	TK	ND	ND	ND	ND	ND	ND	ND	ND	ND	ND	ND	ND	ND	ND	ND	ND	ND	ND	ND	ND	ND

N.A: Not applicable; ND: Not determined; †: putative; Cneg: *Campylobacter* negative; *Cj* : *C. jejuni* ; *Cf* : *C. fetus* ; *Ch* : *C. hyointestinalis*

MIC values in μg/ml; CEF: ceftiofur, TET: tetracycline, AMP: ampicillin, NAL: nalidixic acid, CIP: ciprofloxaxin, ENR: enrofloxacin, KAN: kanamycin

Bold: novel STs. <u>Underlined</u>: CC-ST were determined *in silico* upon analysis of WGS data.

*Isolate D2-7d-42-A is CC21-376, which only has one allele difference with that of CC21-ST8567. Isolate N6-13d-37-A is ST797, which only has one allele difference with that of CC21-ST21.

These two isolates were selected is because no other isolates share the same ST, so the similar STs were selected instead.

Strain N4-48h-37-C was PS (confirmed by AMR test and absence of *tet* (O) and *aph* (3')-III by WGS) but became resistant to tetracycline and kanamycin in MIC test.

Due to the changed antimicrobial resistance and potential mixed culture, strain N4-48h-37-C was skipped for further discussion.
