# Supplementary material for: Impact of Ceftiofur Administration in Steers on the Prevalence and Antimicrobial Resistance of Campylobacter spp
Source: Microorganisms. 2021 Feb 4;9(2):318. doi: 10.3390/microorganisms9020318 (PMC7913856; doi:10.3390/microorganisms9020318)
Supplement: Supplementary file 1 [file microorganisms-09-00318-s001.zip › microorganisms-1058745-S/Supplementary tables.microorganisms-1058745/Table S2.pdf]

Table S2: *Campylobacter* spp. analyzed by multilocus sequence typing in this study

#	Isolate	Steer	Treatment	Sampling point	Incubation temp. (°C)	AM	Species	ST; CC
1	N4-0h-37-A	N4	CHCL	0h	37	PS	*Cj*	21; 21
2	N6-0h-37-A	N6	CHCL	0h	37	PS	*Cj*	21; 21
3	D3-12h-37-A	D3	CCFA	12h	37	PS	*Cj*	21; 21
4	D4-12h-37-A	D4	CCFA	12h	42	PS	*Cj*	21; 21
5	D3-24h-37-B	D3	CCFA	24h	37	PS	*Cj*	21; 21
6	D4-24h-37-B	D4	CCFA	24h	37	PS	*Cj*	21; 21
7	D5-24h-42-A	D5	CCFA	24h	42	PS	*Cj*	21; 21
8	D4-32h-42-D	D4	CCFA	32h	42	TK	*Cj*	21; 21
9	N2-36h-37-C	N2	CHCL	36h	37	PS	*Cj*	21; 21
10	D5-48h-42-B	D5	CCFA	48h	42	PS	*Cj*	21; 21
11	N4-48h-37-C	N4	CHCL	48h	37	PS	*Cj*	21; 21
12	N3-60h-37-B	N3	CHCL	60h	37	PS	*Cj*	21; 21
13	N2-72h-37-B	N2	CHCL	72h	37	PS	*Cj*	21; 21
14	D3-5d-42-A	D3	CCFA	5d	42	PS	*Cj*	21; 21**
15	D3-6d-42-A	D3	CCFA	6d	42	PS	*Cj*	21; 21
16	D3-7d-42-A	D3	CCFA	7d	42	PS	*Cj*	21; 21
17	D4-7d-37-A	D4	CCFA	7d	37	PS	*Cj*	21; 21
18	D5-7d-37-A	D5	CCFA	7d	37	PS	*Cj*	21; 21
19	D2-7d-42-A	D2	CCFA	7d	42	TK	*Cj*	376; 21
20	D4-7d-42-A	D4	CCFA	7d	42	TK	*Cj*	376; 21
21	N6-13d-37-A	N6	CHCL	13d	37	PS	*Cj*	797; 21
22	D1-21d-37-B	D1	CCFA	21d	37	TK	*Cj*	**8565; 21**
23	D1-21d-37-A	D1	CCFA	21d	37	PS	*Cj*	**8566; 21**
24	D2-0h-37-C	D2	CCFA	0h	37	TK	*Cj*	**8567; 21**
25	D2-0h-37-D	D2	CCFA	0h	37	TK	*Cj*	**8567; 21**
26	D2-0h-42-A	D2	CCFA	0h	42	TK	*Cj*	**8567; 21**
27	N1-12h-37-A	N1	CHCL	12h	37	TK	*Cj*	**8567; 21**
28	D4-24h-37-C	D4	CCFA	24h	37	TK	*Cj*	**8567; 21**
29	D2-72h-37-A	D2	CCFA	72h	37	TK	*Cj*	**8567; 21**
30	D2-72h-42-C	D2	CCFA	72h	42	TK	*Cj*	**8567; 21**
31	D4-96h-37-A	D4	CCFA	96h	37	TK	*Cj*	**8567; 21**
32	D2-5d-42-D	D2	CCFA	5d	42	TK	*Cj*	**8567; 21**
33	D2-7d-37-A	D2	CCFA	7d	37	TK	*Cj*	**8567; 21**
34	N4-9d-37-A	N4	CHCL	9d	37	TK	*Cj*	**8567; 21**
35	D6-21d-37-B	D6	CCFA	21d	37	TK	*Cj*	**8567; 21**
36	D4-7d-42-B	D4	CCFA	7d	42	PS	*Cj*	**8573; 21**
37	D6-48h-42-A	D6	CCFA	48h	42	TK	*Cj*	**8576; 21**
38	D3-0h-37-A	D3	CCFA	0h	37	TK	*Cj*	**8221; 61**
39	D3-0h-42-A	D3	CCFA	0h	42	TK	*Cj*	**8221; 61**
40	D4-0h-37-A	D4	CCFA	0h	37	TK	*Cj*	**8221; 61**
41	N5-0h-37-A	N5	CHCL	0h	37	TK	*Cj*	**8221; 61**
42	N3-12h-37-A	N3	CHCL	12h	37	TK	*Cj*	**8221; 61**
43	D1-24h-42-A	D1	CCFA	24h	42	TK	*Cj*	**8221; 61**
44	D6-24h-42-A	D6	CCFA	24h	42	TK	*Cj*	**8221; 61**
45	D1-32h-37-A	D1	CCFA	32h	37	TK	*Cj*	**8221; 61**
46	N2-36h-37-B	N2	CHCL	36h	37	TK	*Cj*	**8221; 61**
47	N4-36h-37-A	N4	CHCL	36h	37	TK	*Cj*	**8221; 61**
48	D5-48h-42-A	D5	CCFA	48h	42	TK	*Cj*	**8221; 61**

Table S2 continued

#	Isolate	Steer	Treatment	Sampling point	Incubation temp. (°C)	AMR	Species	ST; CC
49	D6-72h-37-A	D6	CCFA	72h	37	TK	*Cj*	**8221; 61**
50	D3-72h-42-A	D3	CCFA	72h	42	TK	*Cj*	**8221; 61**
51	D5-96h-37-D	D5	CCFA	96h	37	TK	*Cj*	**8221; 61**
52	N1-96h-37-A	N1	CHCL	96h	37	TK	*Cj*	**8221; 61**
53	D3-5d-37-A	D3	CCFA	5d	37	TK	*Cj*	**8221; 61**
54	N5-5d-37-B	N5	CHCL	5d	37	TK	*Cj*	**8221; 61**
55	N2-7d-37-A	N2	CHCL	7d	37	TK	*Cj*	**8221; 61**
56	D1-8d-42-A	D1	CCFA	8d	42	TK	*Cj*	**8221; 61**
57	N3-10d-37-A	N3	CHCL	10d	37	TK	*Cj*	**8221; 61**
58	N6-13d-37-C	N6	CHCL	13d	37	TK	*Cj*	**8221; 61**
59	D3-14d-42-A	D3	CCFA	14d	42	TK	*Cj*	**8221; 61**
60	D5-12d-37-A	D5	CCFA	12d	37	TK	*Cj*	**8574; 61**
61	D2-12h-37-C	D2	CCFA	12h	37	TN	*Ch*	ST6 (Chy)
62	D2-24h-37-D	D2	CCFA	24h	37	TN	*Ch*	ST6 (Chy)
63	D3-8d-42-B	D3	CCFA	8d	42	TN	*Ch*	ST6 (Chy)
64	D1-12h-37-A	D1	CCFA	12h	37	N	*Ch*	ST41 (Chy)
65	D6-21d-37-D	D6	CCFA	21d	37	N	*Ch*	ST41 (Chy)
66	D6-28d-37-A	D6	CCFA	28d	37	N	*Ch*	ST41 (Chy)
67	D4-12h-37-A	D4	CCFA	12h	37	N	*Cf*	ST6 (Cfe)

Bold: novel STs. ** indicates that the ST was determined *in silico* upon analysis of WGS data.
Cj: C. jejuni; Cf: C. fetus; Ch: C. hyointestinalis
