# Supplementary material for: Impact of Ceftiofur Administration in Steers on the Prevalence and Antimicrobial Resistance of Campylobacter spp
Source: Microorganisms. 2021 Feb 4;9(2):318. doi: 10.3390/microorganisms9020318 (PMC7913856; doi:10.3390/microorganisms9020318)
Supplement: Supplementary file 1 [file microorganisms-09-00318-s001.zip › microorganisms-1058745-S/Supplementary tables.microorganisms-1058745/Table S3.pdf]

Table S3: Antimicrobial minimum inhibitory concentration (MIC) test of representative isolates

				CEF MIC		TET MIC					AMP MIC					NAL		CIP MIC		ENR MIC		KAN MIC	
				Trial			Trial					Trial				Trial		Trial		Trial		Trial	
#	Isolate	AMR	ST; CC	1	2	Level	1	2	3	4	Level	1	2	3	4	1	2	1	2	1	2	1	2
1	D2-0h-37-D	TK	8567; 21	10	10	B	128	128	64	64	B	10	10	32	32	4	4	1	1	1	1	>256	>256
2	D2-32h-37-A	TK	ND	10	10	ND	128	128	ND	ND	ND	10	10	ND	ND	4	4	1	1	1	1	>256	>256
3	D2-72h-37-A	TK	8567; 21	10	10	B	128	128	64	64	B	10	10	32	32	4	4	1	1	1	1	>256	>256
4	D2-7d-37-A	TK	8567; 21	10	10	B	128	128	64	64	B	10	10	32	32	4	4	1	1	1	1	>256	>256
5	D2-0h-42-A	TK	8567; 21	10	10	B	128	128	64	64	B	16	16	32	32	4	4	1	1	1	1	>256	>256
6	D2-32h-42-A	TK	ND	10	10	ND	128	128	ND	ND	ND	16	16	ND	ND	4	4	1	1	1	1	>256	>256
7	D2-72h-42-C	TK	8567; 21	10	10	B	128	128	64	64	B	16	16	32	32	4	4	1	1	1	1	>256	>256
8	D2-7d-42-A	TK	376; 21*	10	10	B	128	128	64	64	B	8	8	32	32	4	4	1	1	1	1	>256	>256
9	D2-7d-42-B	TK	ND	10	10	ND	128	128	ND	ND	ND	8	8	ND	ND	4	4	1	1	1	1	>256	>256
10	D3-0h-37-A	TK	8221; 61	20	20	A	>128	>128	128	128	A	30	30	>32	>32	8	8	1	1	1	1	>256	>256
11	D3-12h-37-F	TK	ND	20	20	ND	>128	>128	ND	ND	ND	30	30	ND	ND	8	8	1	1	1	1	>256	>256
12	D3-24h-37-A	TK	ND	20	20	ND	>128	>128	ND	ND	ND	30	30	ND	ND	8	8	1	1	1	1	>256	>256
13	D3-5d-37-A	TK	8221; 61	20	20	A	>128	>128	128	128	A	30	30	>32	>32	8	8	1	1	1	1	>256	>256
14	**D3-0h-42-A**	TK	8221; 61	20	20	A	>128	>128	128	128	A	16	16	>32	>32	8	8	1	1	1	1	>256	>256
15	D3-12h-42-A	TK	ND	20	20	ND	>128	>128	ND	ND	ND	16	16	ND	ND	8	8	1	1	1	1	>256	>256
16	D3-24h-42-A	TK	ND	20	20	ND	>128	>128	ND	ND	ND	16	16	ND	ND	8	8	1	1	1	1	>256	>256
17	**D3-72h-42-A**	TK	8221; 61	20	20	B	128	128	128	128	A	16	16	>32	>32	8	8	1	1	1	1	>256	>256
18	D3-5d-42-A	TK	*21; 21*	20	20	A	>128	>128	128	128	B	16	16	32	32	8	8	1	1	1	1	>256	>256
19	**D3-14d-42-A**	TK	8221; 61	20	20	A	>128	>128	128	128	A	16	16	>32	>32	8	8	1	1	1	1	>256	>256
20	**N5-0h-37-A**	TK	8221; 61	20	20	A	>128	>128	128	128	A	8	8	>32	>32	8	8	1	1	1	1	>256	>256
21	**N5-5d-37-B**	TK	8221; 61	20	20	A	>128	>128	128	128	B	16	16	32	32	8	8	1	1	1	1	>256	>256
22	N6-0h-37-A	PS	21; 21	20	20	B	1	1	1	1	A	32	32	>32	>32	4	4	1	1	1	1	1	1
23	N6-0h-37-B	PS	ND	20	20	ND	1	1	ND	ND	ND	32	32	ND	ND	4	4	1	1	1	1	1	1
24	N6-13d-37-A	PS	797; 21**	20	20	B	1	1	1	1	A	32	32	>32	>32	4	4	1	1	1	1	1	1
25	N6-13d-37-B	PS	ND	20	20	ND	1	1	ND	ND	ND	32	32	ND	ND	4	4	1	1	1	1	1	1

ND: not determined; MIC values in μg/ml; CEF: ceftiofur, TET: tetracycline, AMP: ampicillin, NAL: nalidixic acid, CIP: ciprofloxaxin, ENR: enrofloxacin, KAN: kanamycin
Isolate name: take D2-0h-37-D as an example, this means isolate D is originated from fecal sample that was collected at 0 hour from calf D2, then incubated at 37°C.

* Only one allele *(tkt)* difference between ST8567 and ST376; no other isolates share the same ST.

** Only one allele (*uncA*) difference between ST21 and ST797; no other isolates share the same ST.

Bold: isolates that were selected for MIC strip test. Selection criteria: strains that had different MIC levels for TET or AMP but were the same for the other features (steer origin, incubation temperature, AMR, and ST).

Italic: the CC-ST is determined *in silico* for strain D3-5d-42-A upon analysis of whole genome sequencing data.
